# Author Correction: CBX3 antagonizes IFNγ/STAT1/PD-L1 axis to modulate colon inflammation and CRC chemosensitivity

**DOI:** 10.1038/s44321-024-00141-y

**Published:** 2024-09-16

**Authors:** Yao Xiang, Jorge Mata-Garrido, Yuanji Fu, Christophe Desterke, Eric Batsché, Ahmed Hamaï, Christine Sedlik, Youssouf Sereme, David Skurnik, Abdelali Jalil, Rachel Onifarasoaniaina, Eric Frapy, Jean-Christophe Beche, Razack Alao, Eliane Piaggio, Laurence Arbibe, Yunhua Chang

**Affiliations:** 1 Université Paris Cité, INSERM, CNRS, Institut Necker Enfants Malades, F-75015 Paris, France; 2 Université Paris-Saclay, INSERM, Laboratory of Modèles de cellules souches malignes et thérapeutiques, Villejuif, F-94805 France; 3 Sorbonne Université, Institut de Biologie Paris-Seine, CNRS UMR8256 Biological Adaptation and Aging (IBPS), Laboratory of Epigenetics and RNA Metabolism in Human Diseases, 75005 Paris, France; 4 Institut Curie, PSL University, Department of Translational Research, Inserm U932, Laboratory of Immunity and Cancer, F-75005 Paris, France; 5 Service de Bactériologie, virologie, parasitologie et hygiène, AP-HP, Hôpital Necker, F-75015 Paris, France; 6 Université Paris Cité, CNRS, SPPIN - Saints-Pères Paris Institute for the Neurosciences, F-75006 Paris, France; 7 Université de Paris Cité, INSERM, CNRS, Institut Cochin, F-75014 Paris, France; 8 Laboratory of Expérimentation Animale et Transgénèse SFR Necker-Inserm US 24, Paris, France

## Abstract

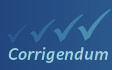

**Correction to:** *EMBO Molecular Medicine* (2024) 16:1404–1426. 10.1038/s44321-024-00066-6 | Published online 29 April 2024

The authors contacted the journal after an error was detected in the Methods section.

**The Methods section is corrected**.

The following text is corrected from:

*Cbx3*KO Mouse model

The Villin-creERT2:*Cbx3*^-/-^ mouse model and Tamoxifen administration were produced as previously described (Mata-Garrido et al, 2022). Briefly, Tamoxifen (0.5 mg/mouse) diluted in 20% clinOleic acid was administrated by oral gavage, at 3 doses every 5 days in Cbx3Flox/Flox;Tg(Villin-CreERT2) mice, noted as *Cbx3* Villin-Cre or *Cbx3* KO mice in the later text. Control Cbx3 Villin-Cre mice received 20% clinOleic acid alone by oral gavage. Additional controls using Cbx3Flox/Flox mice that do not express the Cre recombinase were identically treated with Tamoxifen (Fig. EV5). Animal studies were approved by the ethical committee of Paris Descartes University (authorization number 17-022). The mice were housed in a maximum of 5 ventilated cages in accordance with their social needs, with water and food provided ad libitum. To ensure the best possible housing conditions for the animals, cardboard houses and tunnels, as well as wooden sticks for gnawing, were added to the cage to reduce stress and anxiety for the mice. The mice were monitored daily.

To: (Changes in bold)

*Cbx3*KO Mouse model

The Villin-creERT2:*Cbx3*^-/-^ mouse model and Tamoxifen administration were produced as previously described (Mata-Garrido et al, 2022). Briefly, Tamoxifen **(10** **mg/mouse, calculated based on 0.5** **mg/g of body weight, with each mouse weighing approximately 20** **g)** diluted in 20% clinOleic acid was administrated by oral gavage, at 3 doses every 5 days in Cbx3Flox/Flox;Tg(Villin-CreERT2) mice, noted as *Cbx3* Villin-Cre or *Cbx3* KO mice in the later text. Control Cbx3 Villin-Cre mice received 20% clinOleic acid alone by oral gavage. Additional controls using Cbx3Flox/Flox mice that do not express the Cre recombinase were identically treated with Tamoxifen (Fig. EV5). Animal studies were approved by the ethical committee of Paris Descartes University (authorization number 17-022). The mice were housed in a maximum of 5 ventilated cages in accordance with their social needs, with water and food provided ad libitum. To ensure the best possible housing conditions for the animals, cardboard houses and tunnels, as well as wooden sticks for gnawing, were added to the cage to reduce stress and anxiety for the mice. The mice were monitored daily.

This correction does not affect the conclusions of the manuscript.

