# Supplementary figures and images for: CBX3 antagonizes IFNγ/STAT1/PD-L1 axis to modulate colon inflammation and CRC chemosensitivity (part 1 of 2)
Source: EMBO Mol Med. 2024 Apr 29;16(6):10. doi: 10.1038/s44321-024-00066-6 (PMC11178889; doi:10.1038/s44321-024-00066-6)

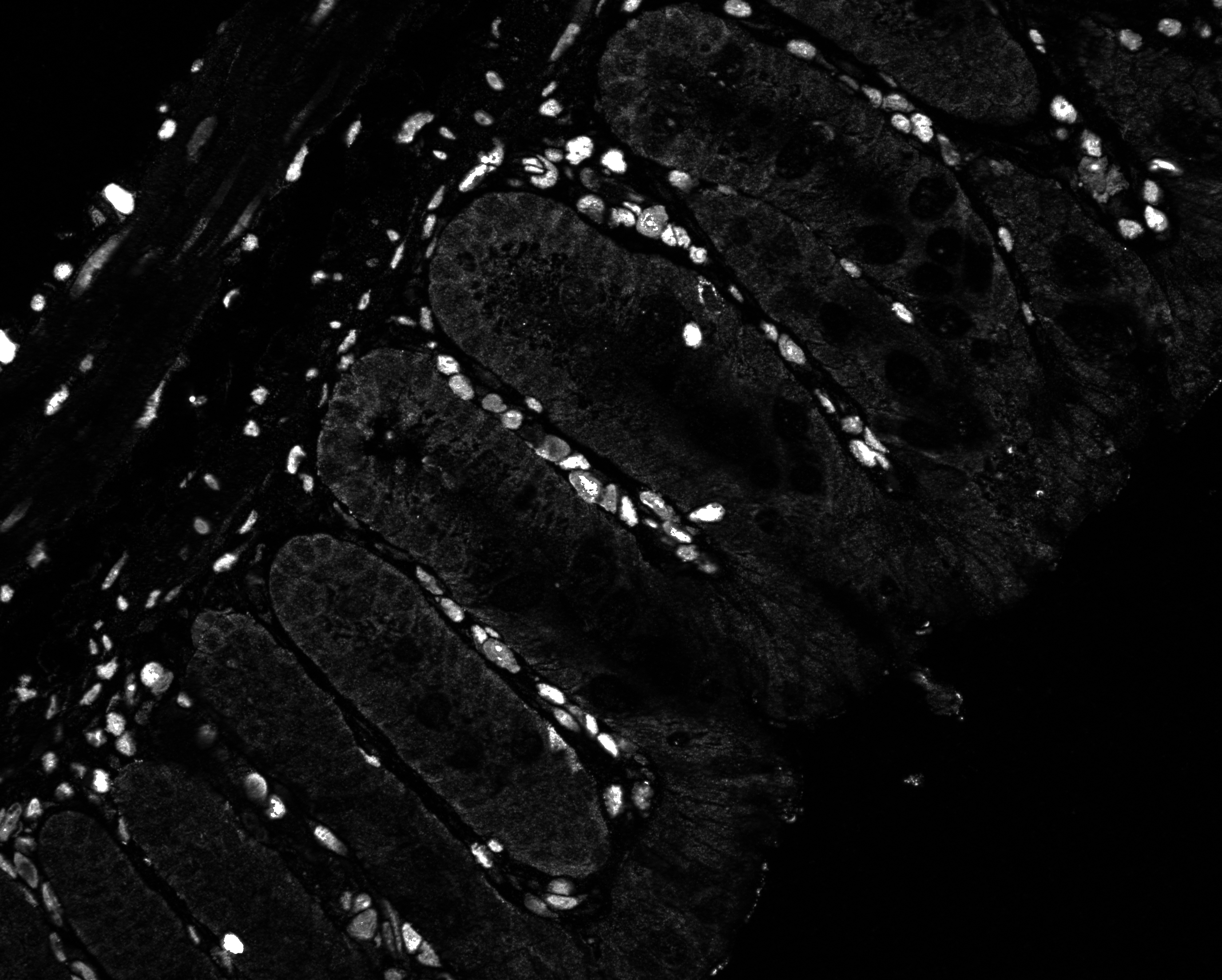

Supplement: Supplementary file 4 — Source data Fig. 1 [file 44321_2024_66_MOESM4_ESM.zip › Figure 1/1B/40x may19026 HP1gamme1.tif_files/40x may19026 HP1gamme 1_h0b0c1x0-2752y0-2208.tif]

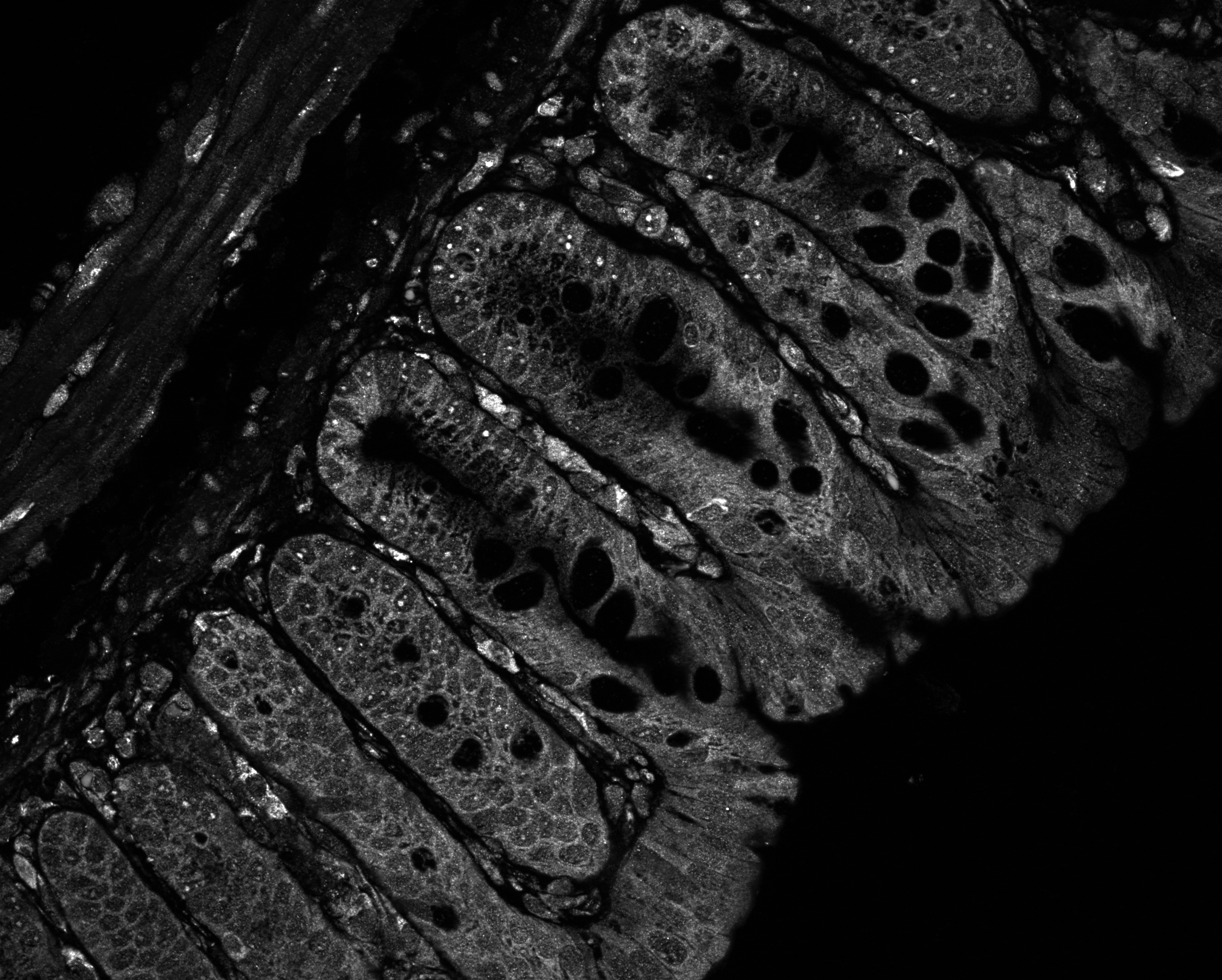

Supplement: Supplementary file 4 — Source data Fig. 1 [file 44321_2024_66_MOESM4_ESM.zip › Figure 1/1B/40x may19026 HP1gamme1.tif_files/40x may19026 HP1gamme 1_h0b0c2x0-2752y0-2208.tif]

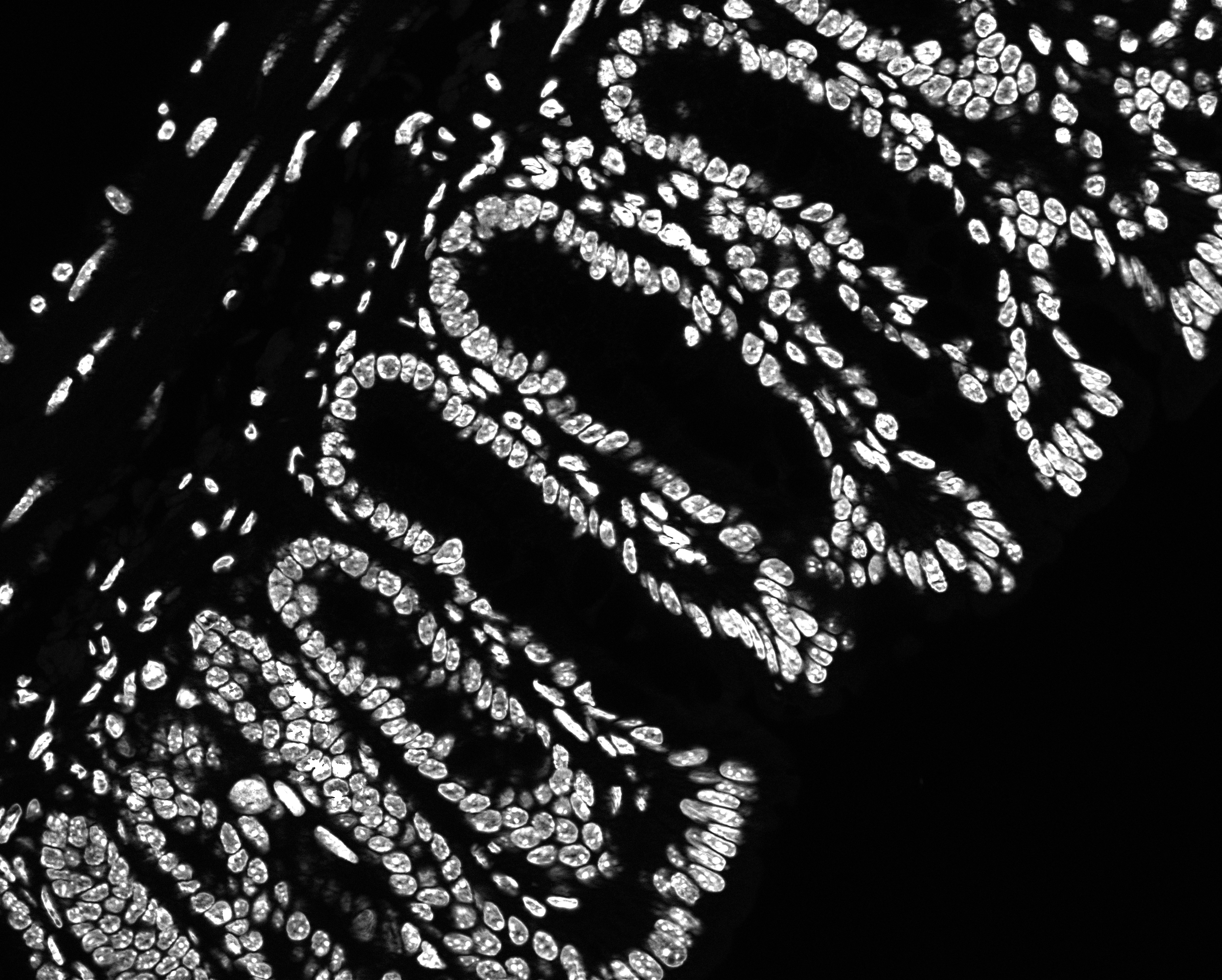

Supplement: Supplementary file 4 — Source data Fig. 1 [file 44321_2024_66_MOESM4_ESM.zip › Figure 1/1B/40x may19026 HP1gamme1.tif_files/40x may19026HP1gamma 1_h0b0c0x0-2752y0-2208.tif]

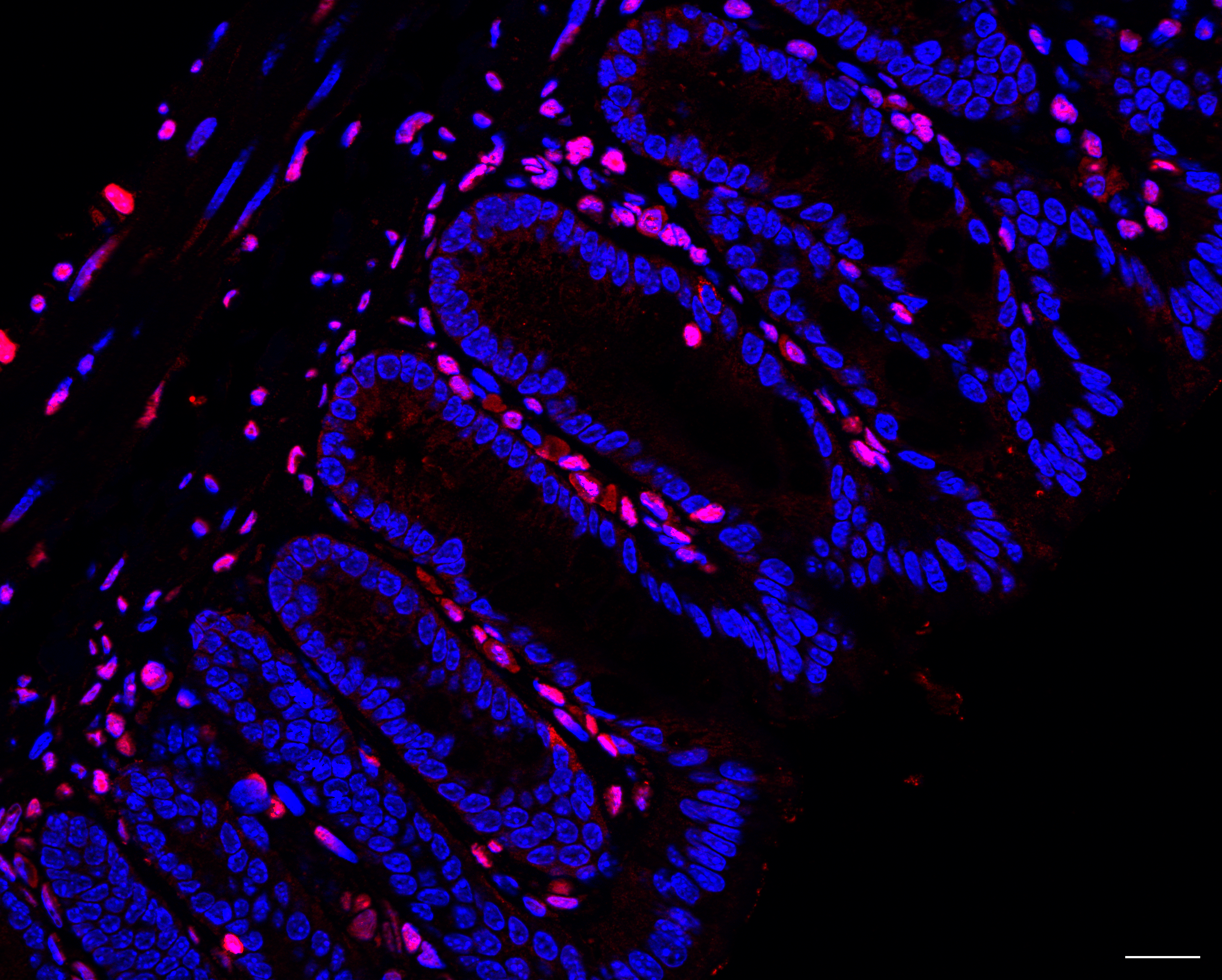

Supplement: Supplementary file 4 — Source data Fig. 1 [file 44321_2024_66_MOESM4_ESM.zip › Figure 1/1B/40x may19026 HP1gamme1.tif_files/40x may19026HP1gamma.tif]

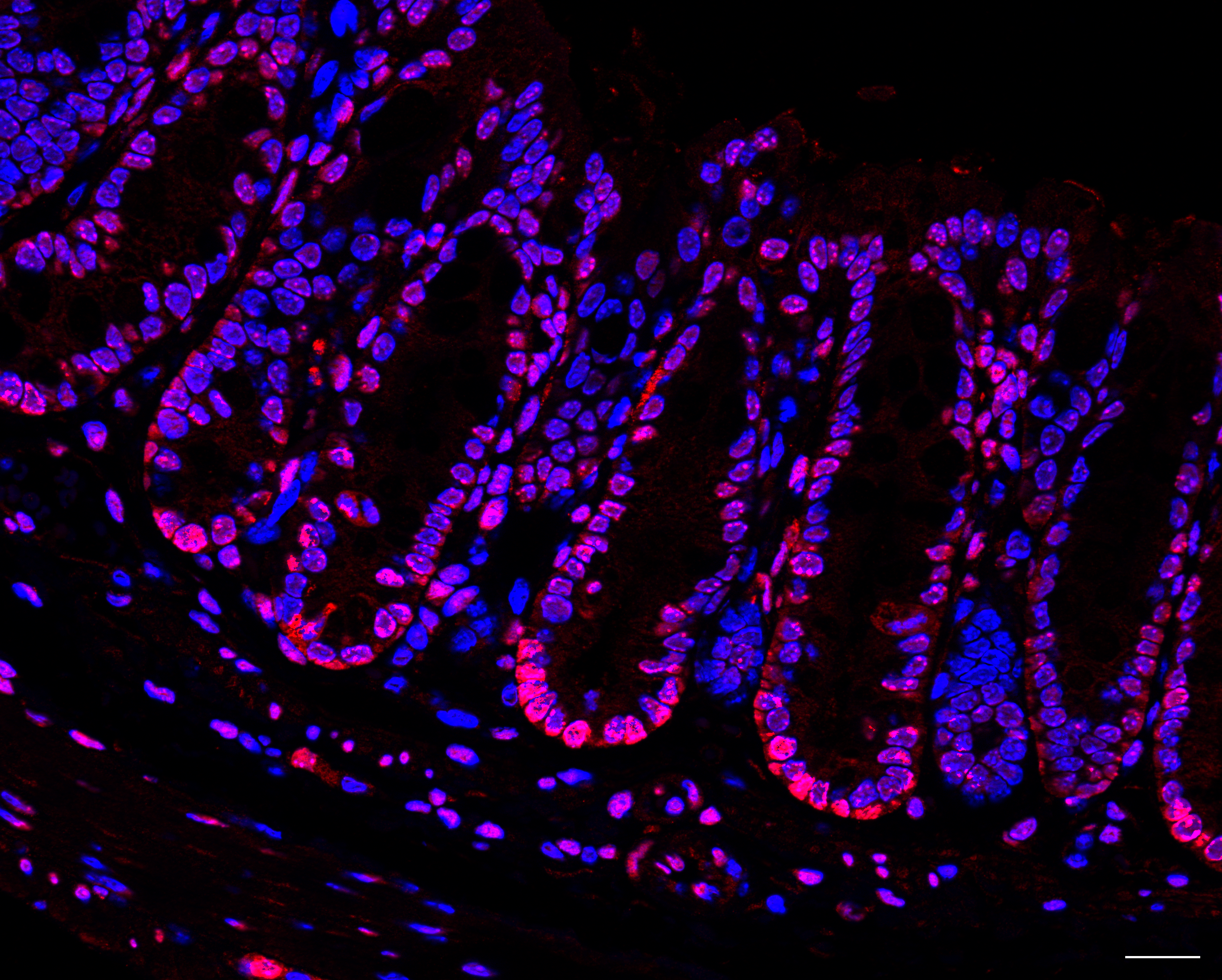

Supplement: Supplementary file 4 — Source data Fig. 1 [file 44321_2024_66_MOESM4_ESM.zip › Figure 1/1B/40x may19032 HP1gamme 2.tif_files/40x may19032 HP1gamme 2 bar.tif]

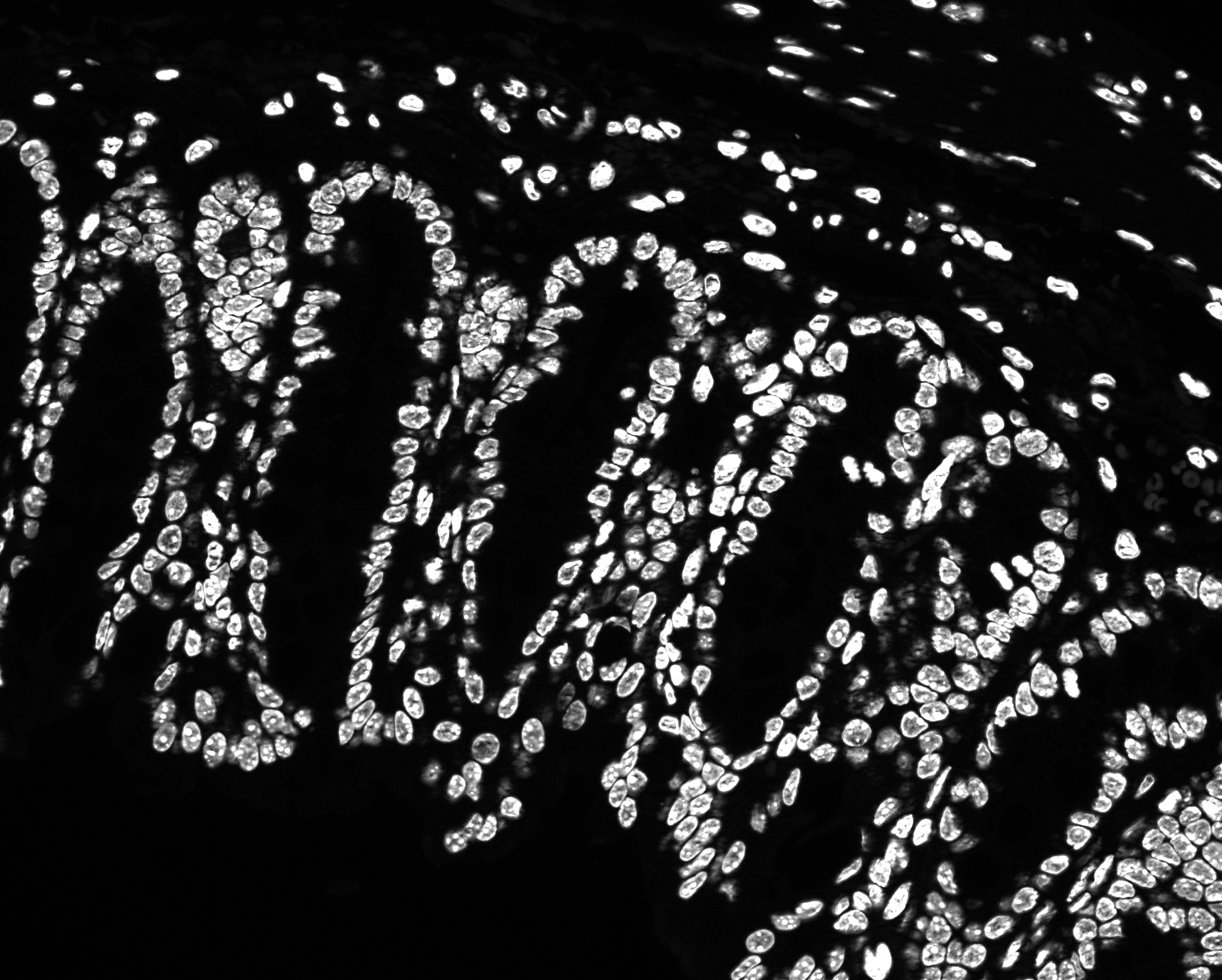

Supplement: Supplementary file 4 — Source data Fig. 1 [file 44321_2024_66_MOESM4_ESM.zip › Figure 1/1B/40x may19032 HP1gamme 2.tif_files/40x may19032 HP1gamme 2_h0b0c0x0-2752y0-2208.tif]

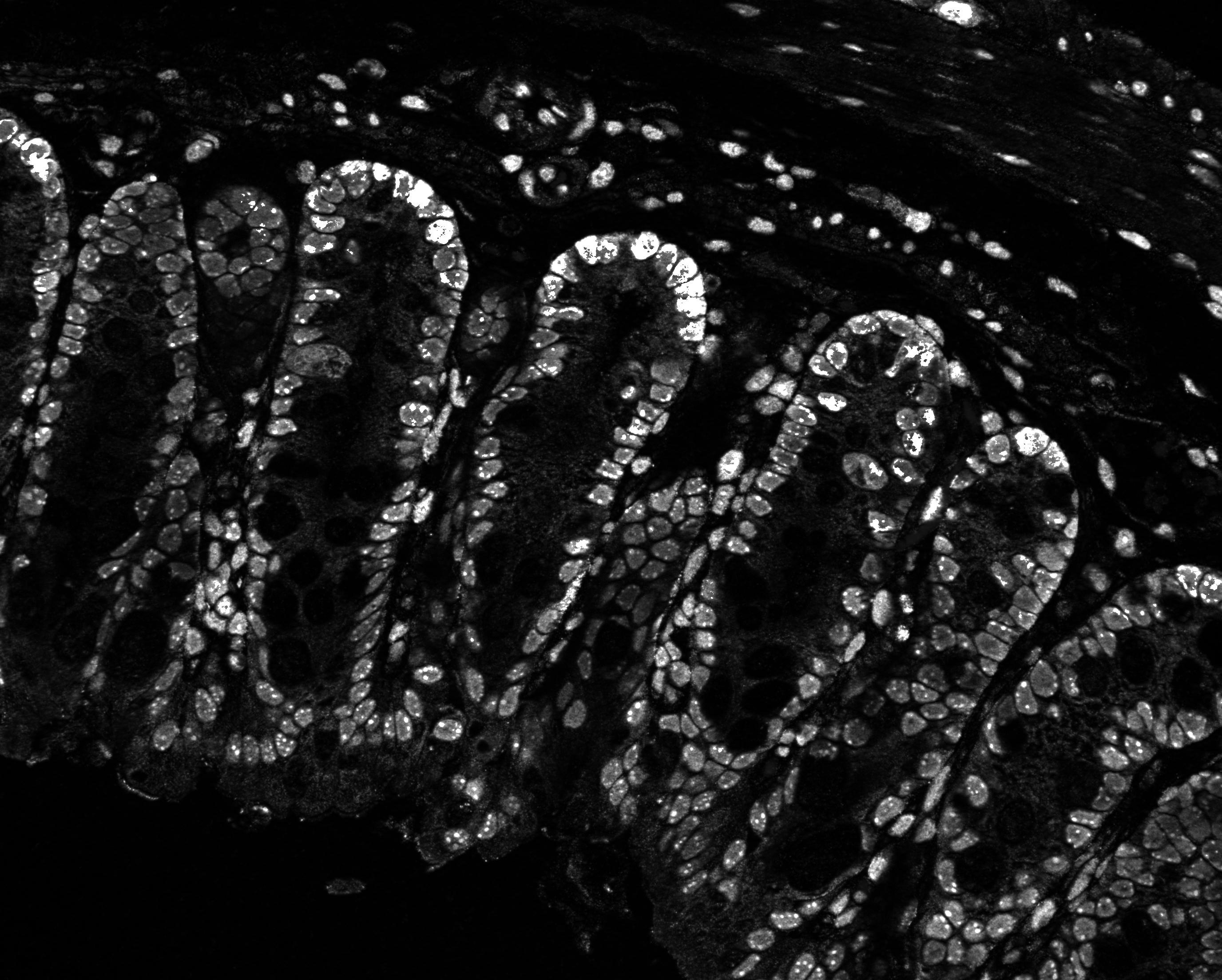

Supplement: Supplementary file 4 — Source data Fig. 1 [file 44321_2024_66_MOESM4_ESM.zip › Figure 1/1B/40x may19032 HP1gamme 2.tif_files/40x may19032 HP1gamme 2_h0b0c1x0-2752y0-2208.tif]

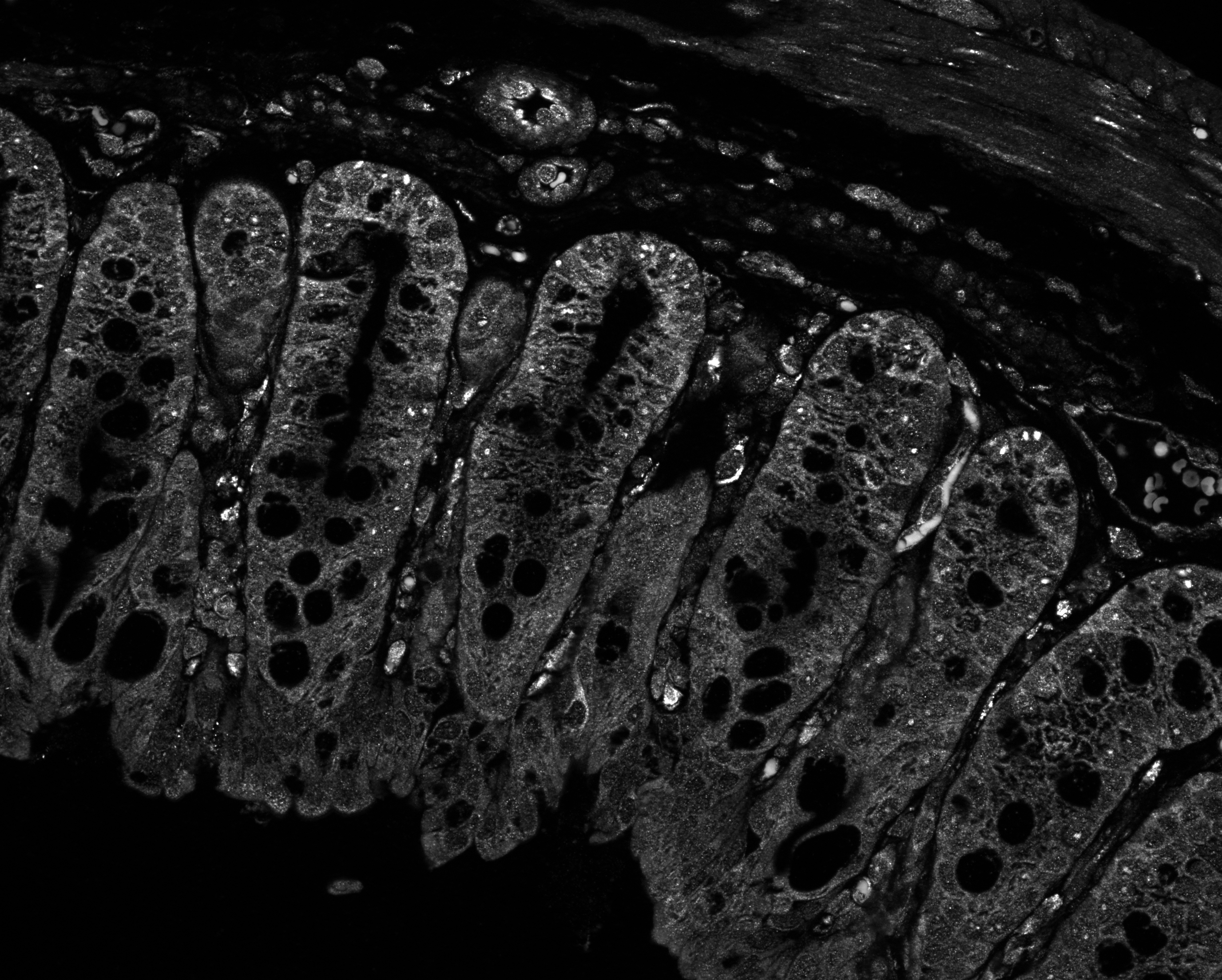

Supplement: Supplementary file 4 — Source data Fig. 1 [file 44321_2024_66_MOESM4_ESM.zip › Figure 1/1B/40x may19032 HP1gamme 2.tif_files/40x may19032 HP1gamme 2_h0b0c2x0-2752y0-2208.tif]

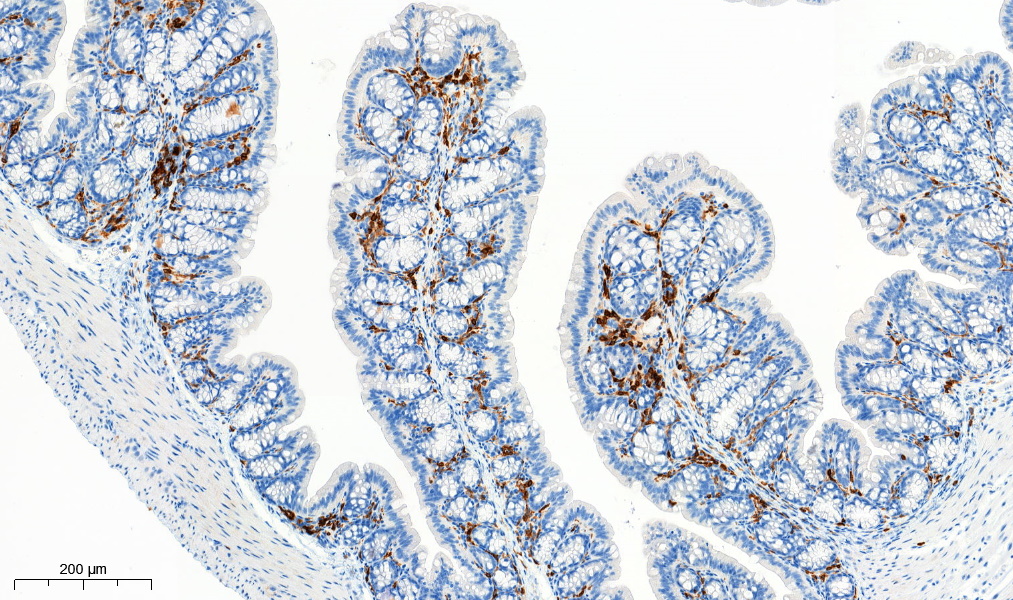

Supplement: Supplementary file 4 — Source data Fig. 1 [file 44321_2024_66_MOESM4_ESM.zip › Figure 1/1C/19026 proxim KO.jpg]

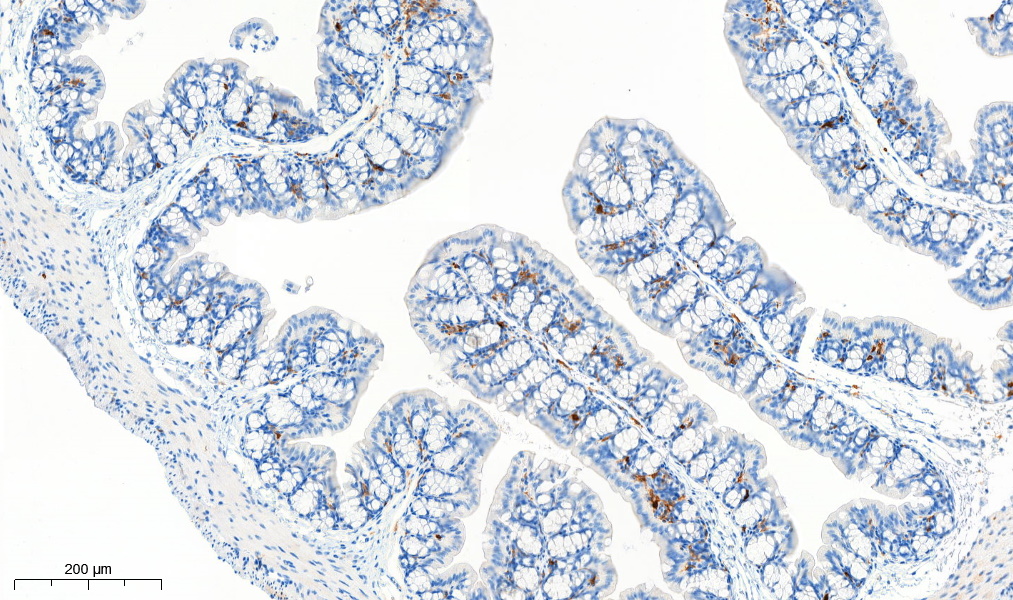

Supplement: Supplementary file 4 — Source data Fig. 1 [file 44321_2024_66_MOESM4_ESM.zip › Figure 1/1C/19030 proxim WT.jpg]

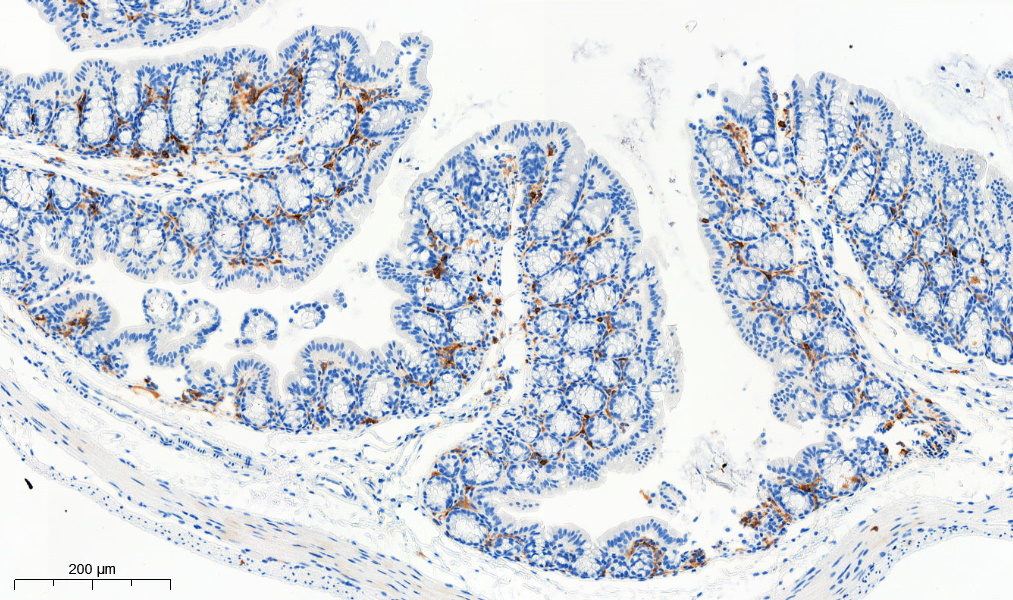

Supplement: Supplementary file 4 — Source data Fig. 1 [file 44321_2024_66_MOESM4_ESM.zip › Figure 1/1C/20007 proxim KO bis.jpg]

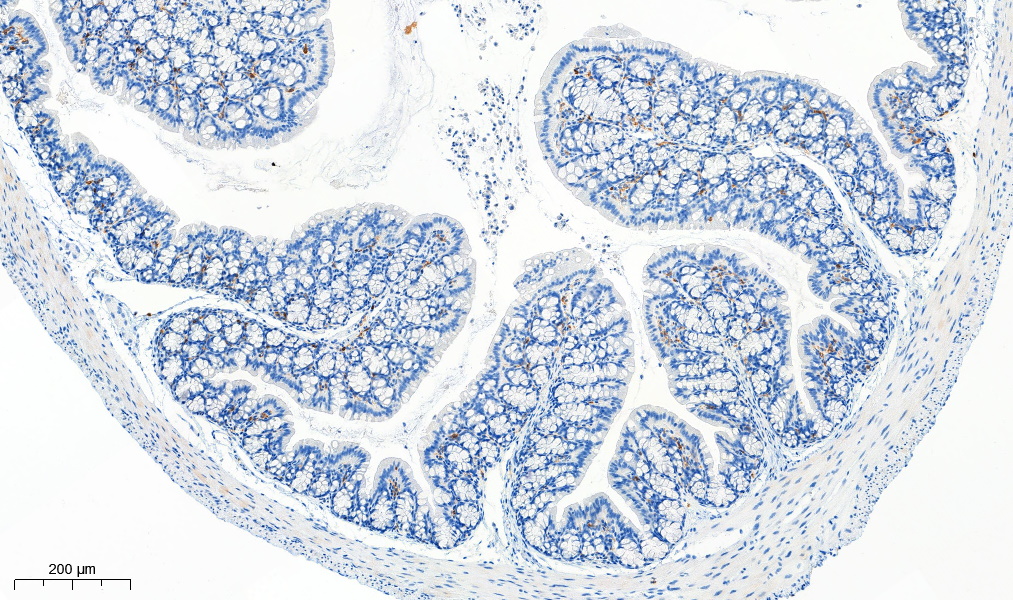

Supplement: Supplementary file 4 — Source data Fig. 1 [file 44321_2024_66_MOESM4_ESM.zip › Figure 1/1C/20011 proxim WT.jpg]

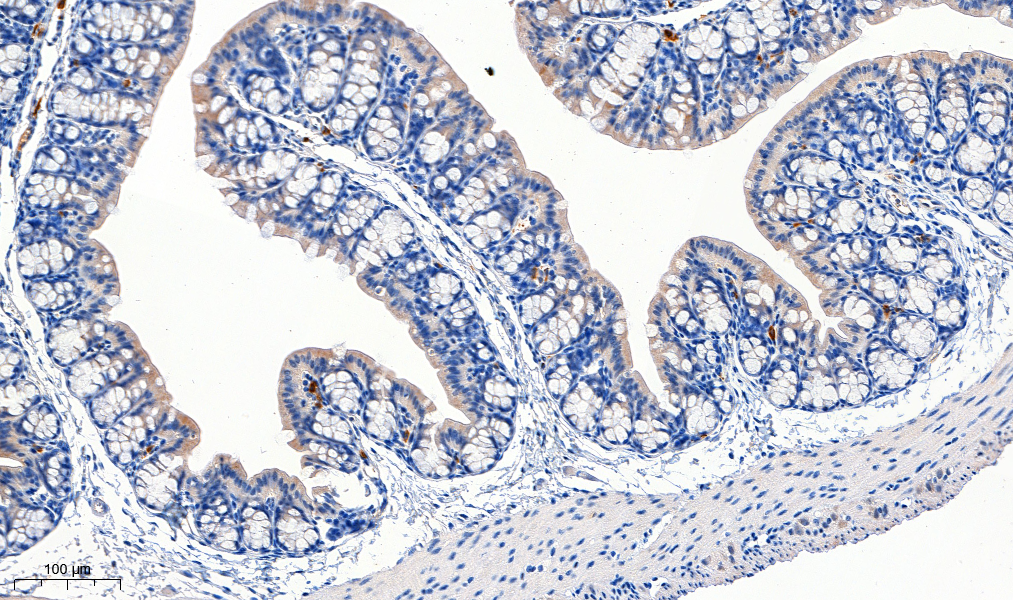

Supplement: Supplementary file 4 — Source data Fig. 1 [file 44321_2024_66_MOESM4_ESM.zip › Figure 1/1E/19030WT_20.0x.tif]

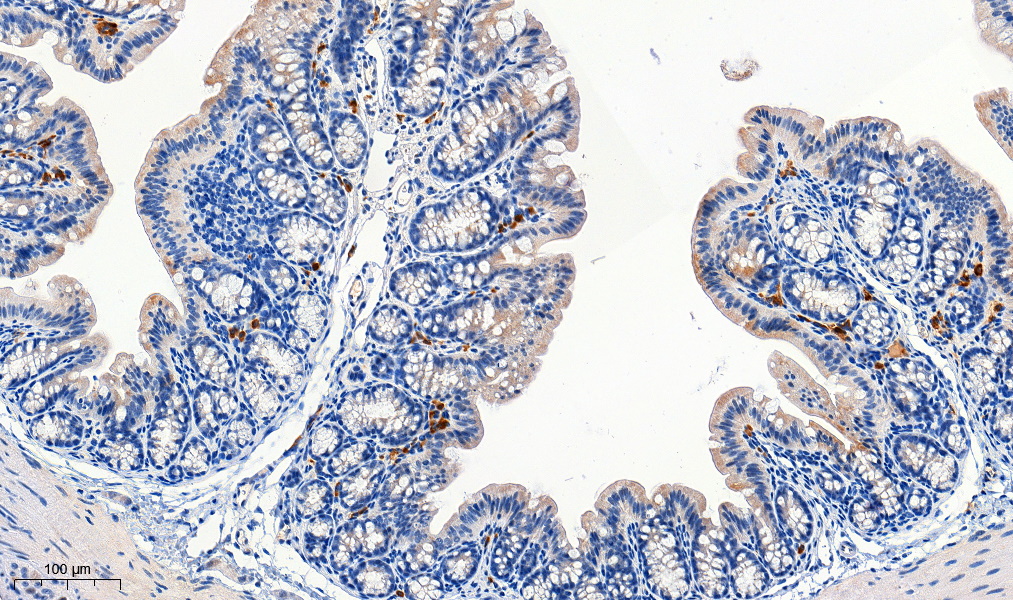

Supplement: Supplementary file 4 — Source data Fig. 1 [file 44321_2024_66_MOESM4_ESM.zip › Figure 1/1E/Ly6G19026KO_20.0x.jpg]

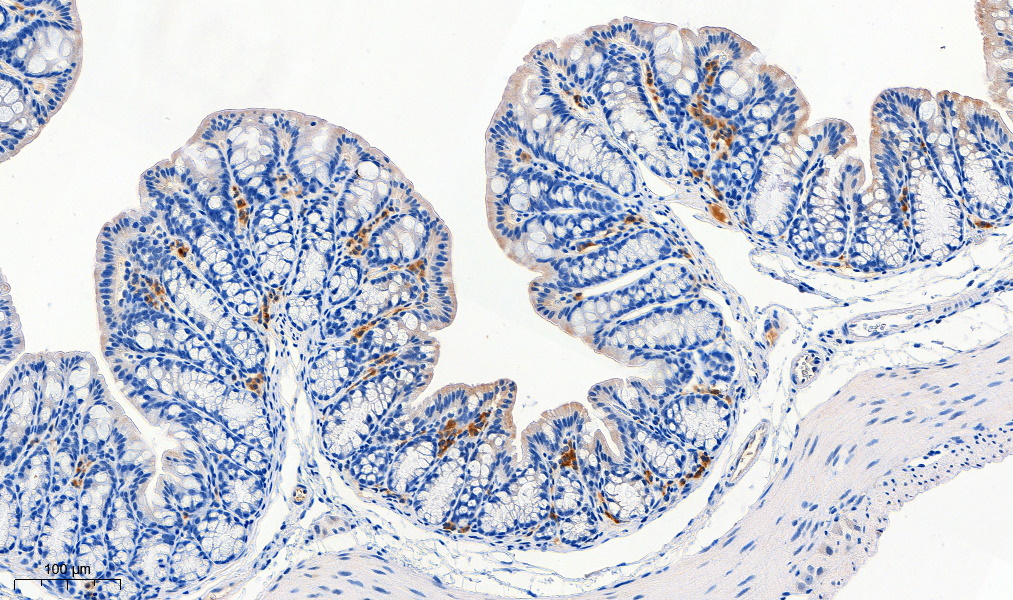

Supplement: Supplementary file 4 — Source data Fig. 1 [file 44321_2024_66_MOESM4_ESM.zip › Figure 1/1E/Ly6G20010KO_20.0xbis.jpg]

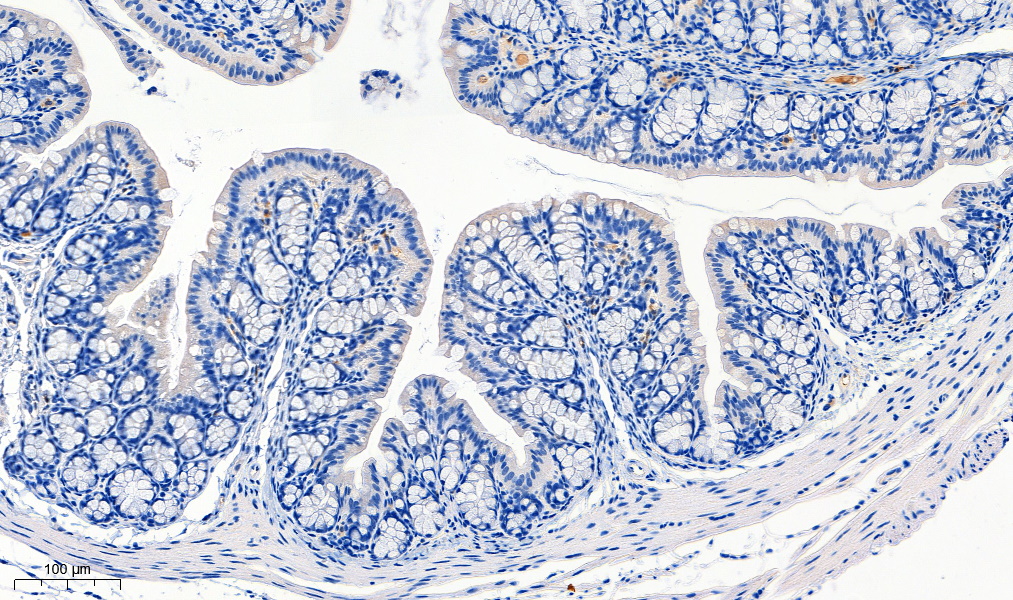

Supplement: Supplementary file 4 — Source data Fig. 1 [file 44321_2024_66_MOESM4_ESM.zip › Figure 1/1E/Ly6G20013WT_20.0x.jpg]

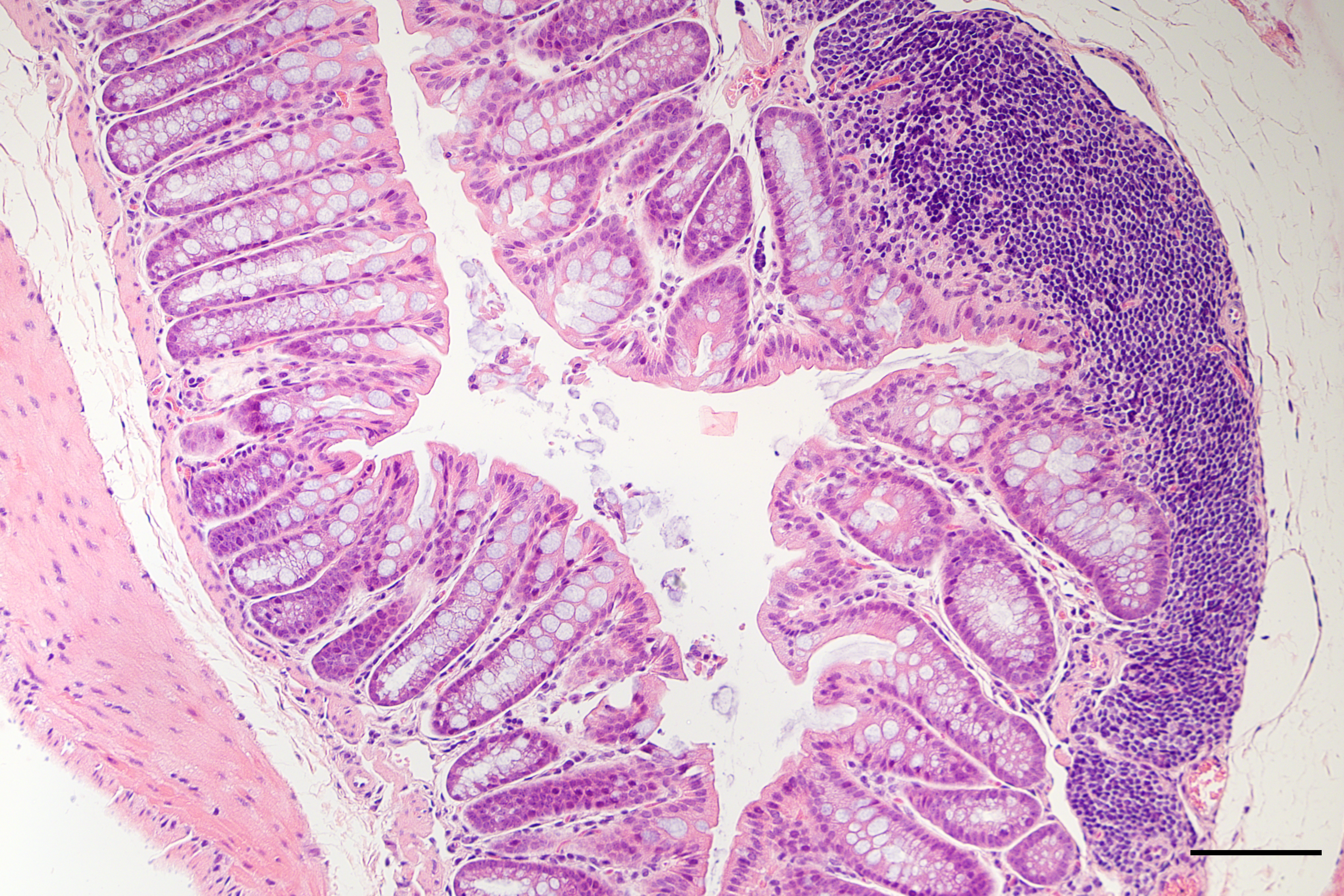

Supplement: Supplementary file 4 — Source data Fig. 1 [file 44321_2024_66_MOESM4_ESM.zip › Figure 1/1G/Nov20007KO.tif (RGB).tif]

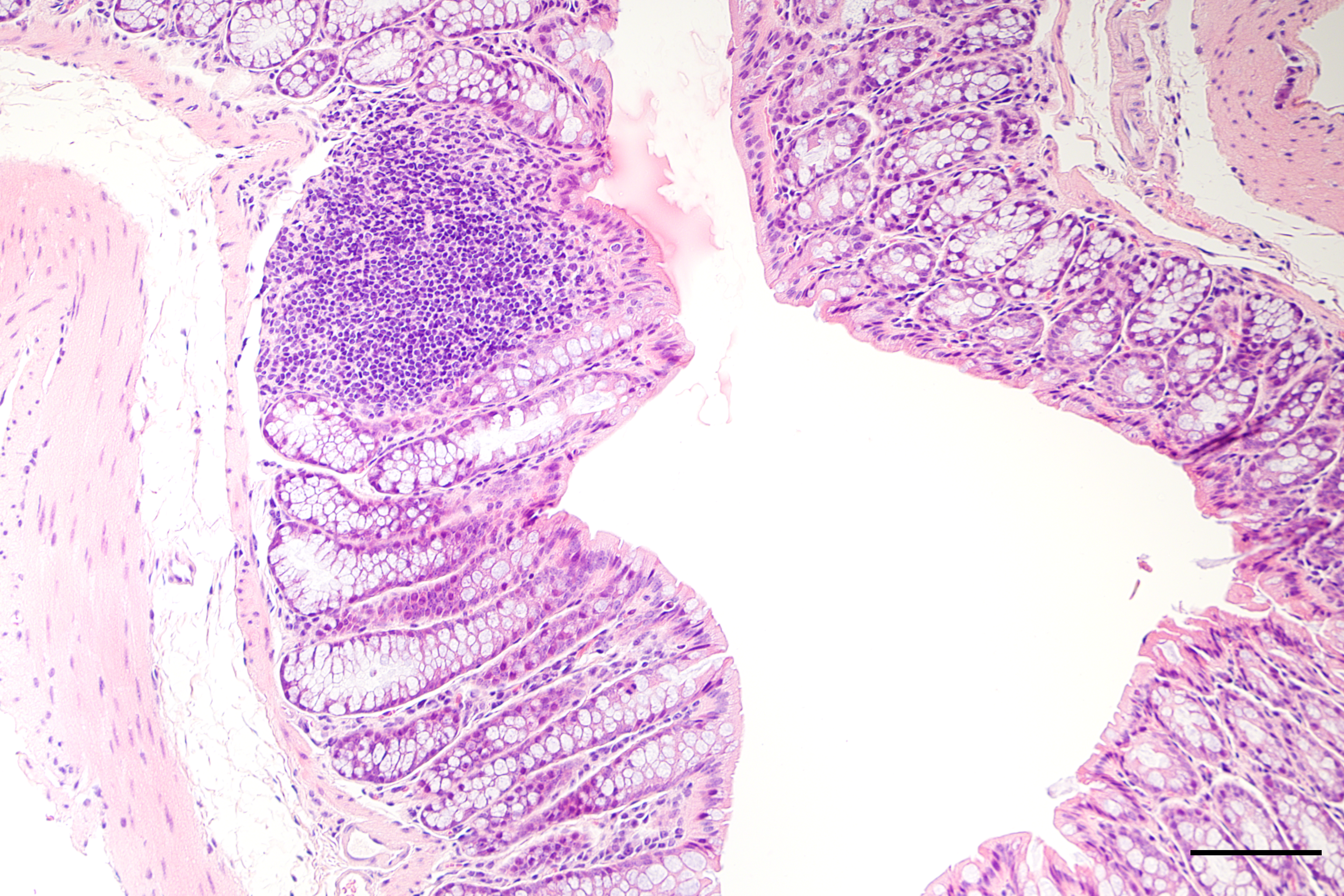

Supplement: Supplementary file 4 — Source data Fig. 1 [file 44321_2024_66_MOESM4_ESM.zip › Figure 1/1G/Nov20013WT.tif (RGB).tif]

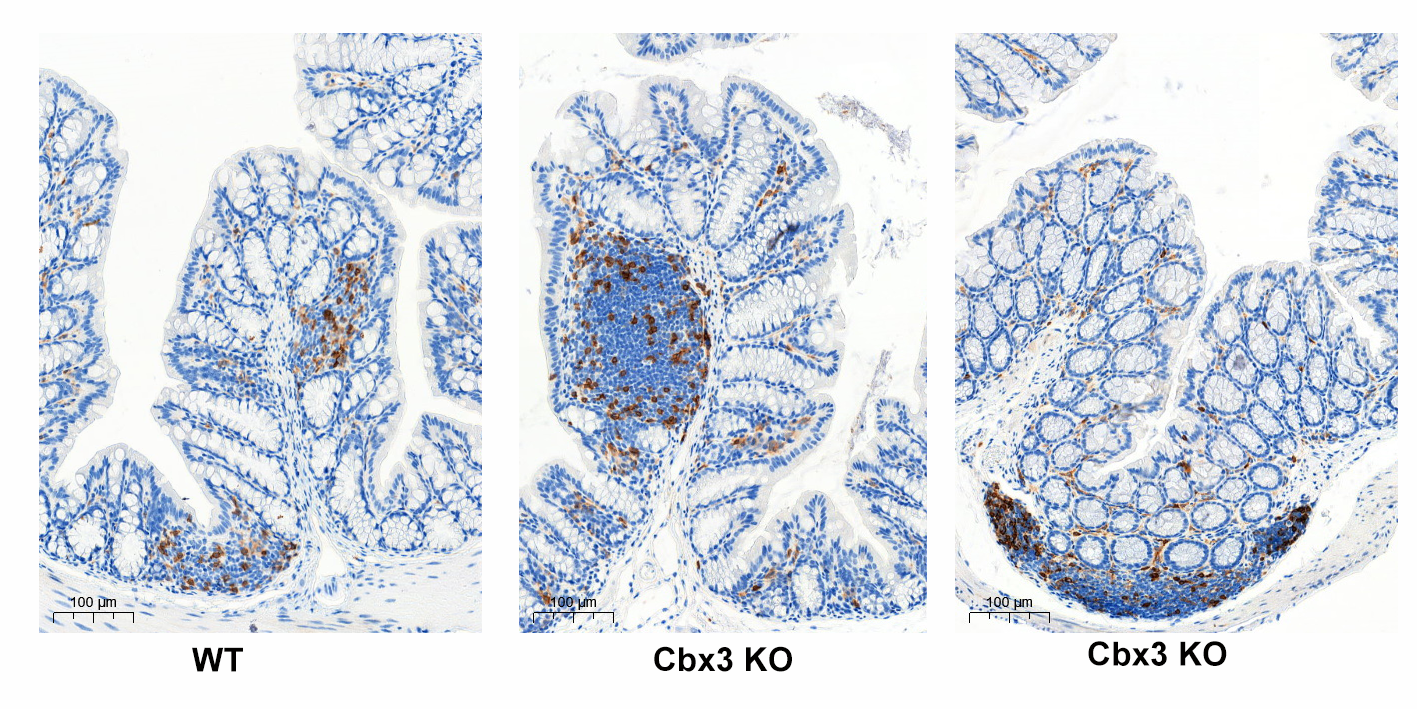

Supplement: Supplementary file 4 — Source data Fig. 1 [file 44321_2024_66_MOESM4_ESM.zip › Figure 1/1H/CD4 lymphocyte nodule.tif]

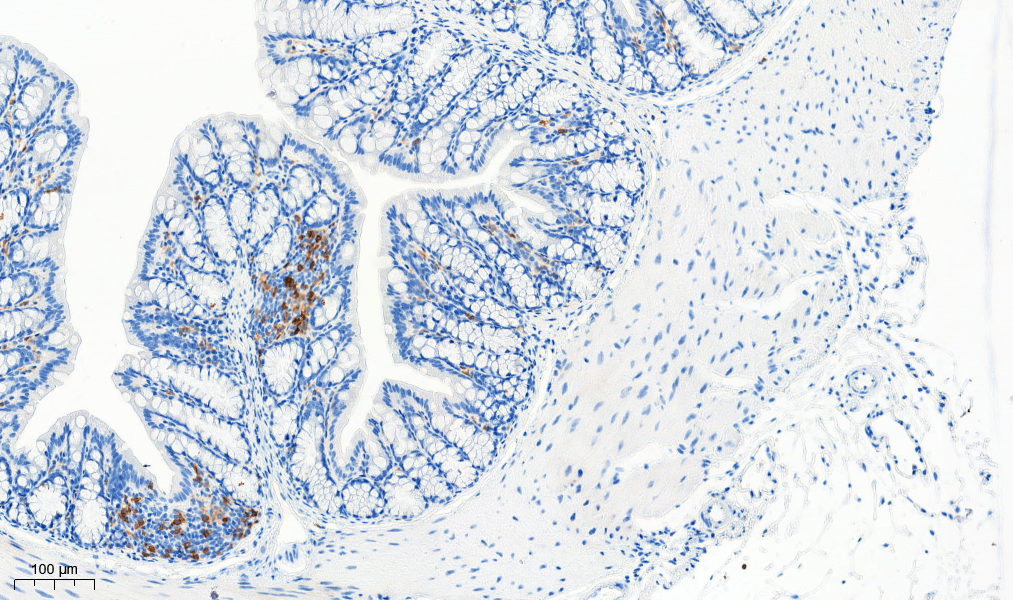

Supplement: Supplementary file 4 — Source data Fig. 1 [file 44321_2024_66_MOESM4_ESM.zip › Figure 1/1H/Nov20012_15.2x.tif]

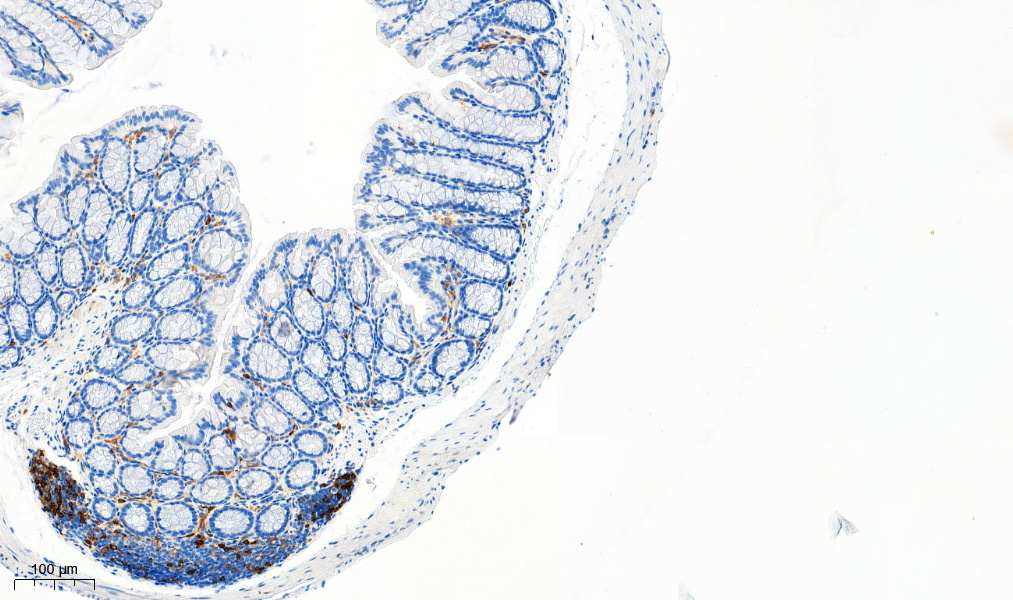

Supplement: Supplementary file 4 — Source data Fig. 1 [file 44321_2024_66_MOESM4_ESM.zip › Figure 1/1H/Nov2008_15.2x.jpg]

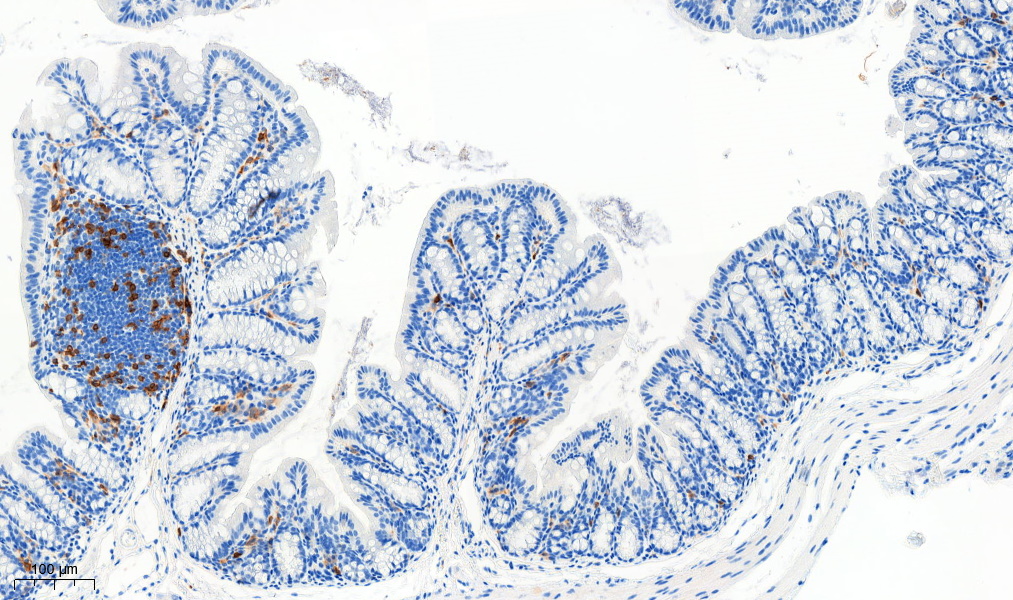

Supplement: Supplementary file 4 — Source data Fig. 1 [file 44321_2024_66_MOESM4_ESM.zip › Figure 1/1H/Nov2009_15.2x.jpg]

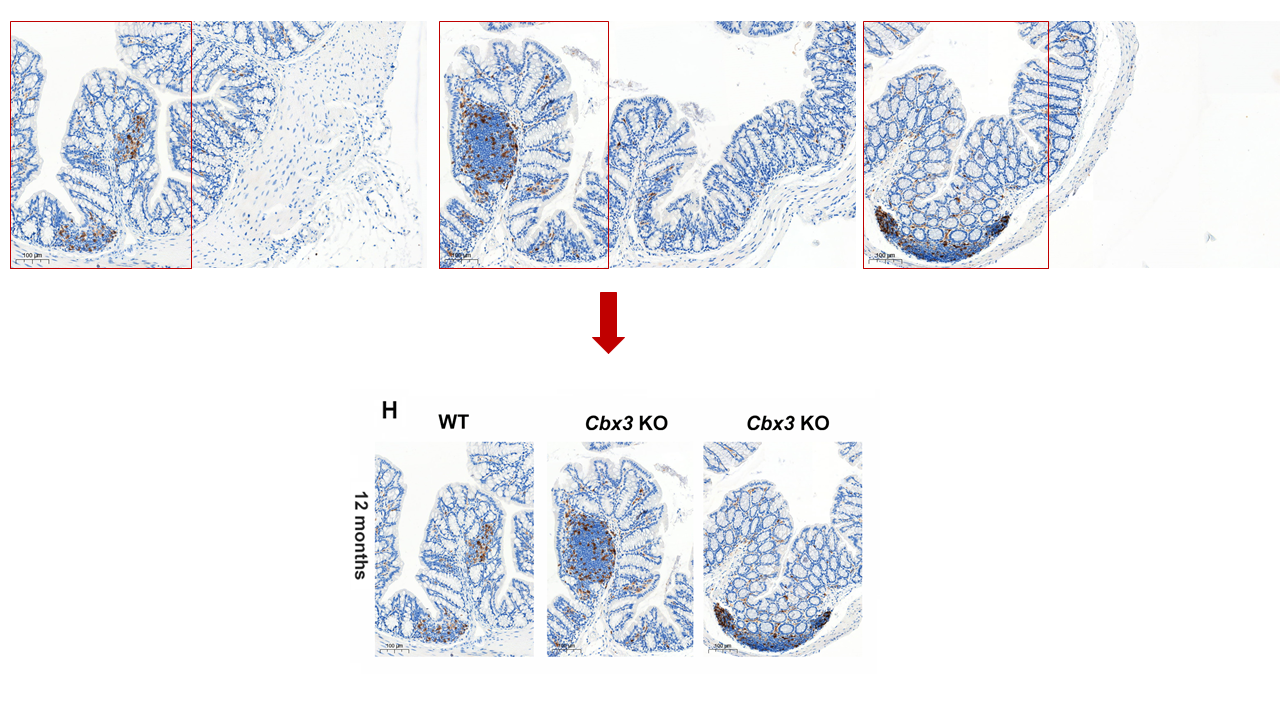

Supplement: Supplementary file 4 — Source data Fig. 1 [file 44321_2024_66_MOESM4_ESM.zip › Figure 1/1H/resume.tif]

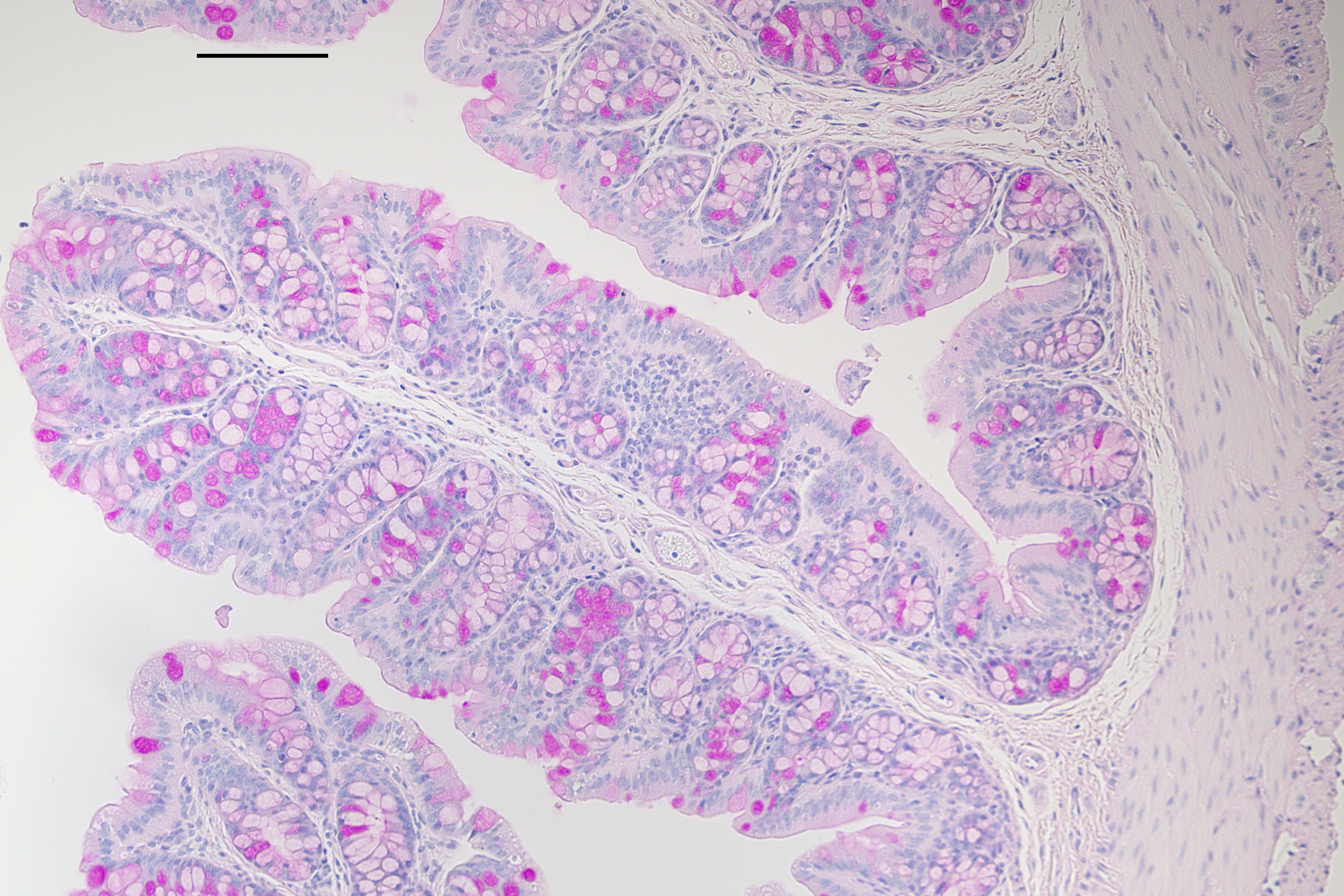

Supplement: Supplementary file 5 — Source data Fig. 2 [file 44321_2024_66_MOESM5_ESM.zip › Figure 2/2A/may19036day7ko 10x 1.tif (RGB).tif]

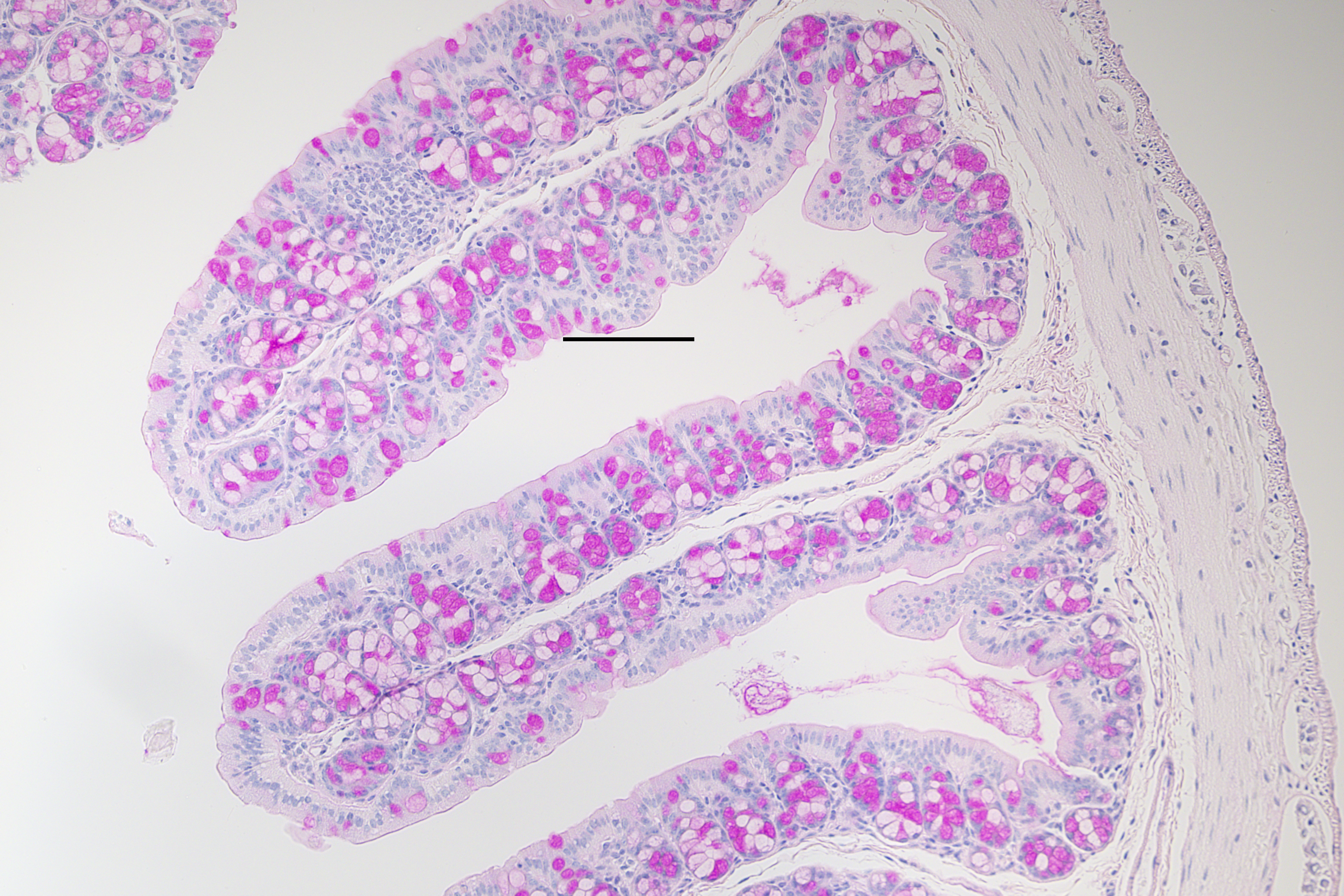

Supplement: Supplementary file 5 — Source data Fig. 2 [file 44321_2024_66_MOESM5_ESM.zip › Figure 2/2A/may19038 wt 10x.tif (RGB).tif]

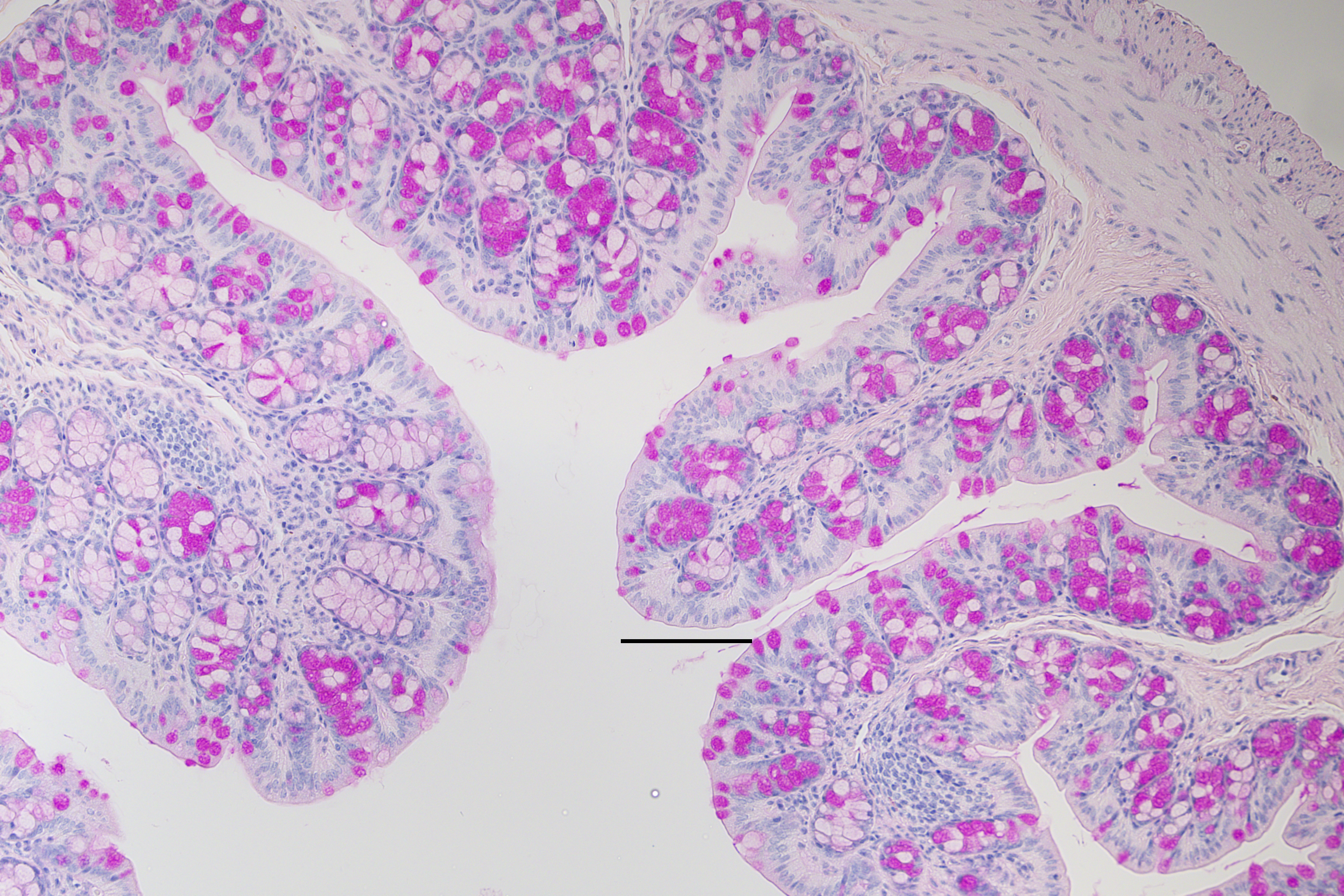

Supplement: Supplementary file 5 — Source data Fig. 2 [file 44321_2024_66_MOESM5_ESM.zip › Figure 2/2A/nov200013 10x 4.tif (RGB).tif]

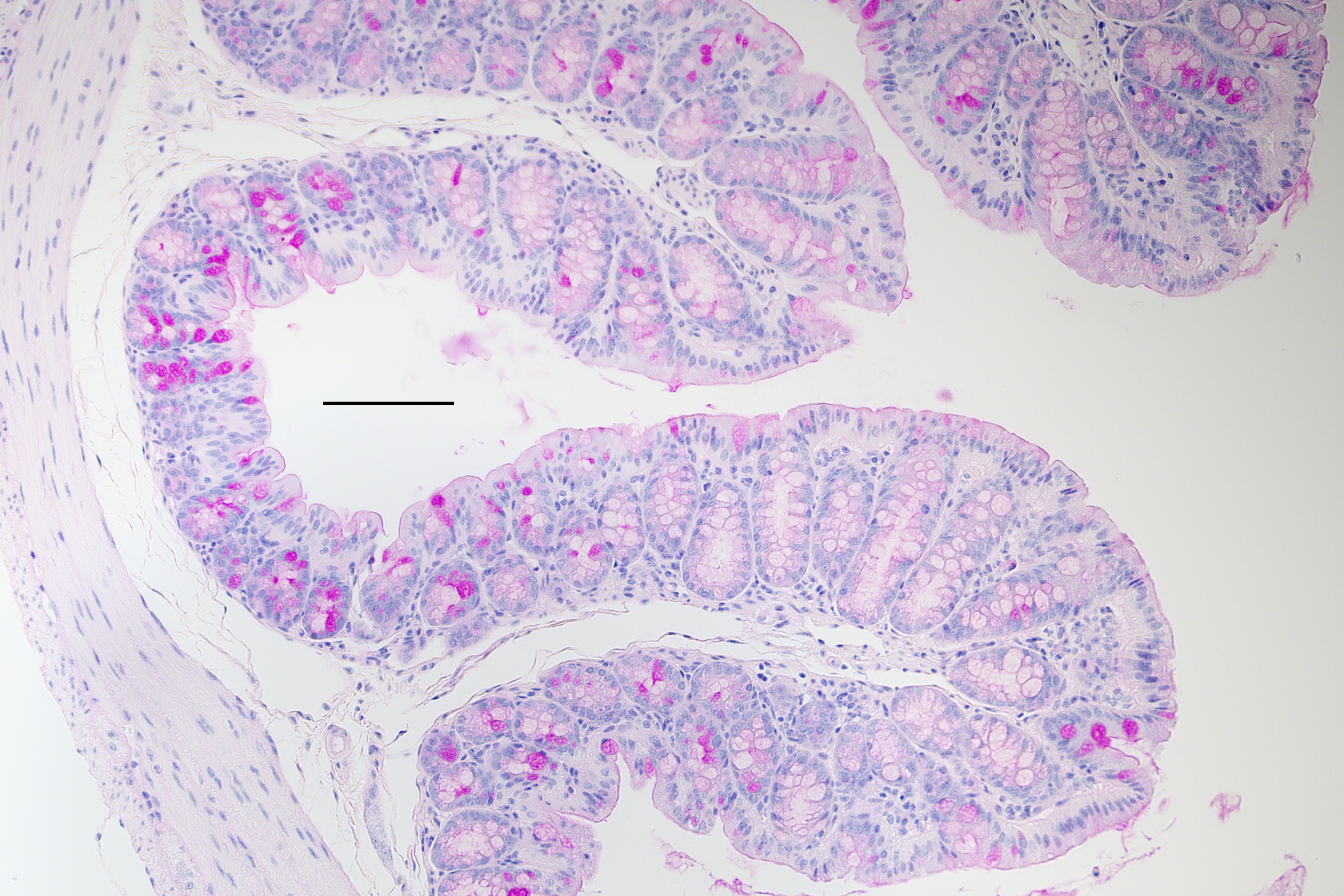

Supplement: Supplementary file 5 — Source data Fig. 2 [file 44321_2024_66_MOESM5_ESM.zip › Figure 2/2A/nov20007 10x 2.tif (RGB).tif]

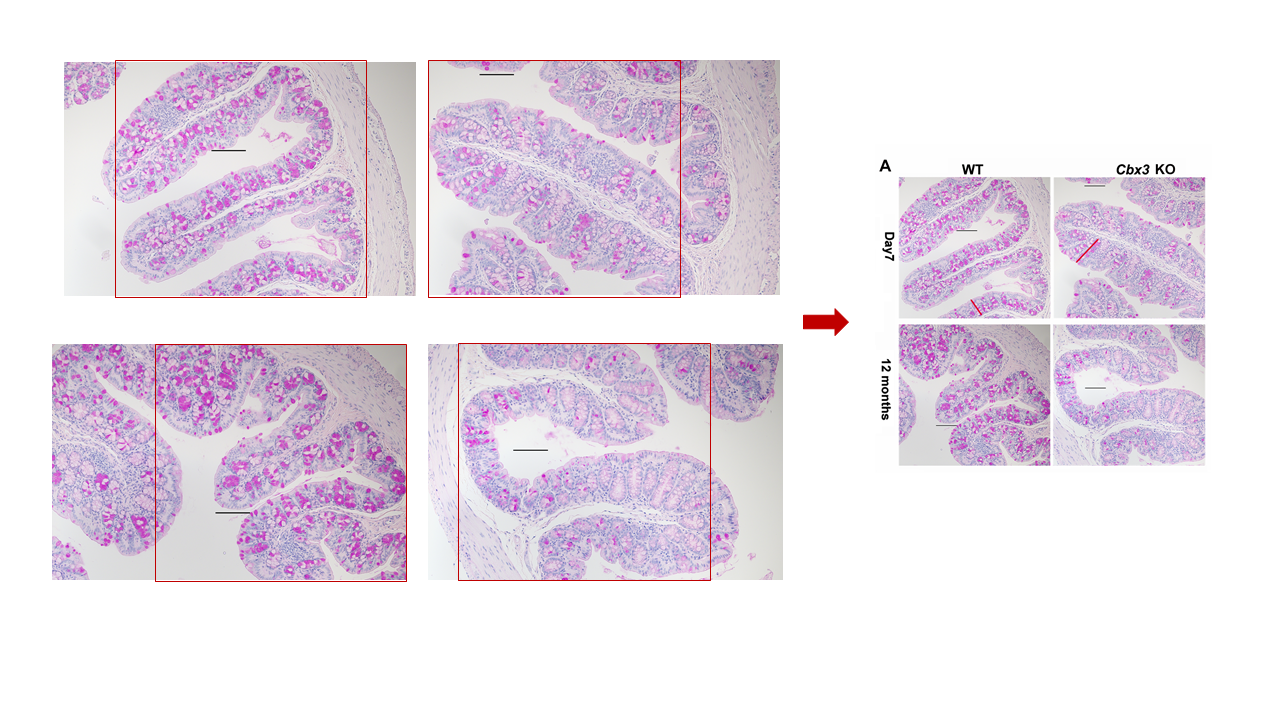

Supplement: Supplementary file 5 — Source data Fig. 2 [file 44321_2024_66_MOESM5_ESM.zip › Figure 2/2A/resume.tif]

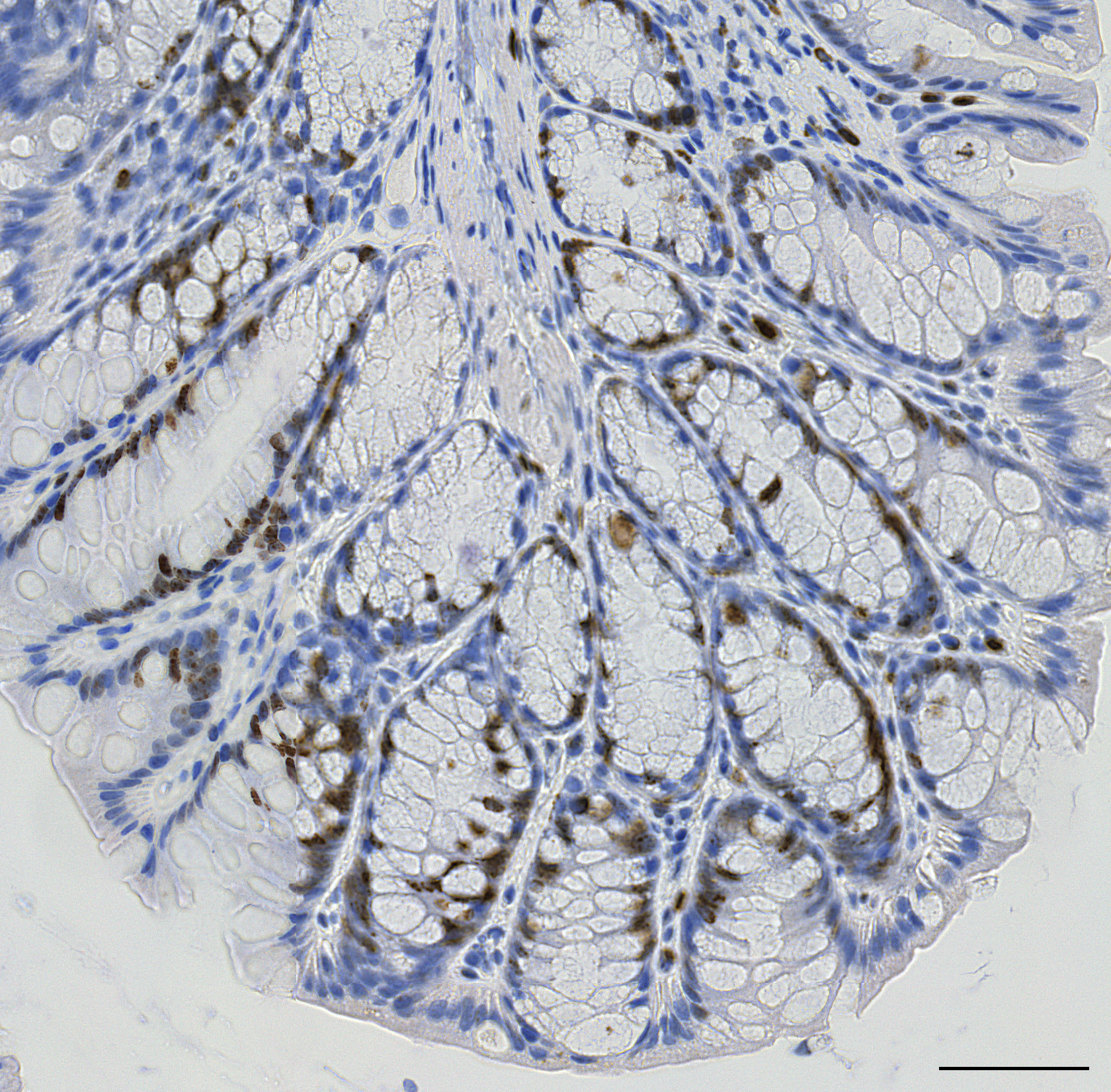

Supplement: Supplementary file 5 — Source data Fig. 2 [file 44321_2024_66_MOESM5_ESM.zip › Figure 2/2C/Yunhua-ki67-KO.tif]

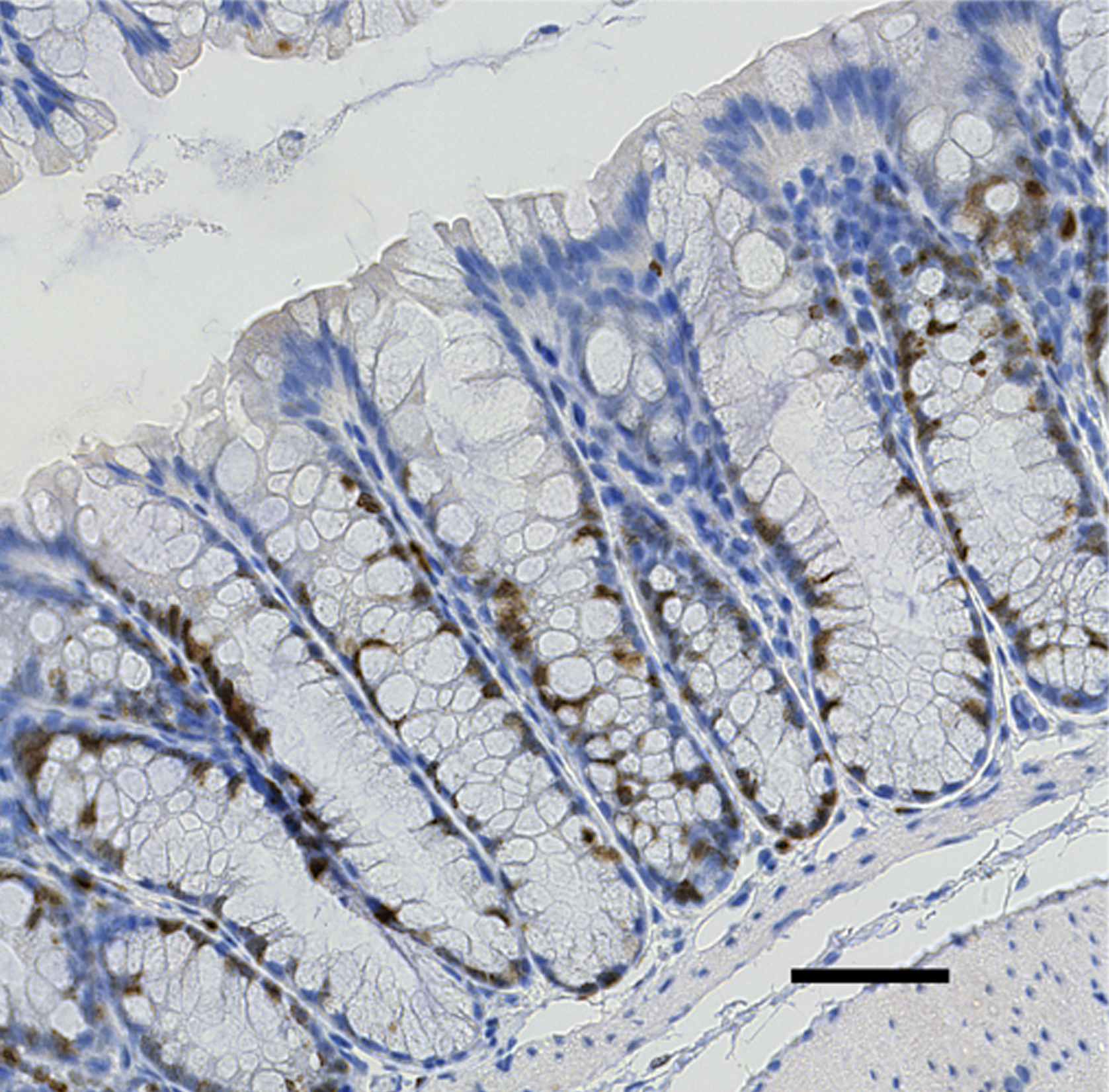

Supplement: Supplementary file 5 — Source data Fig. 2 [file 44321_2024_66_MOESM5_ESM.zip › Figure 2/2C/Yunhua-ki67-KO1.tif]

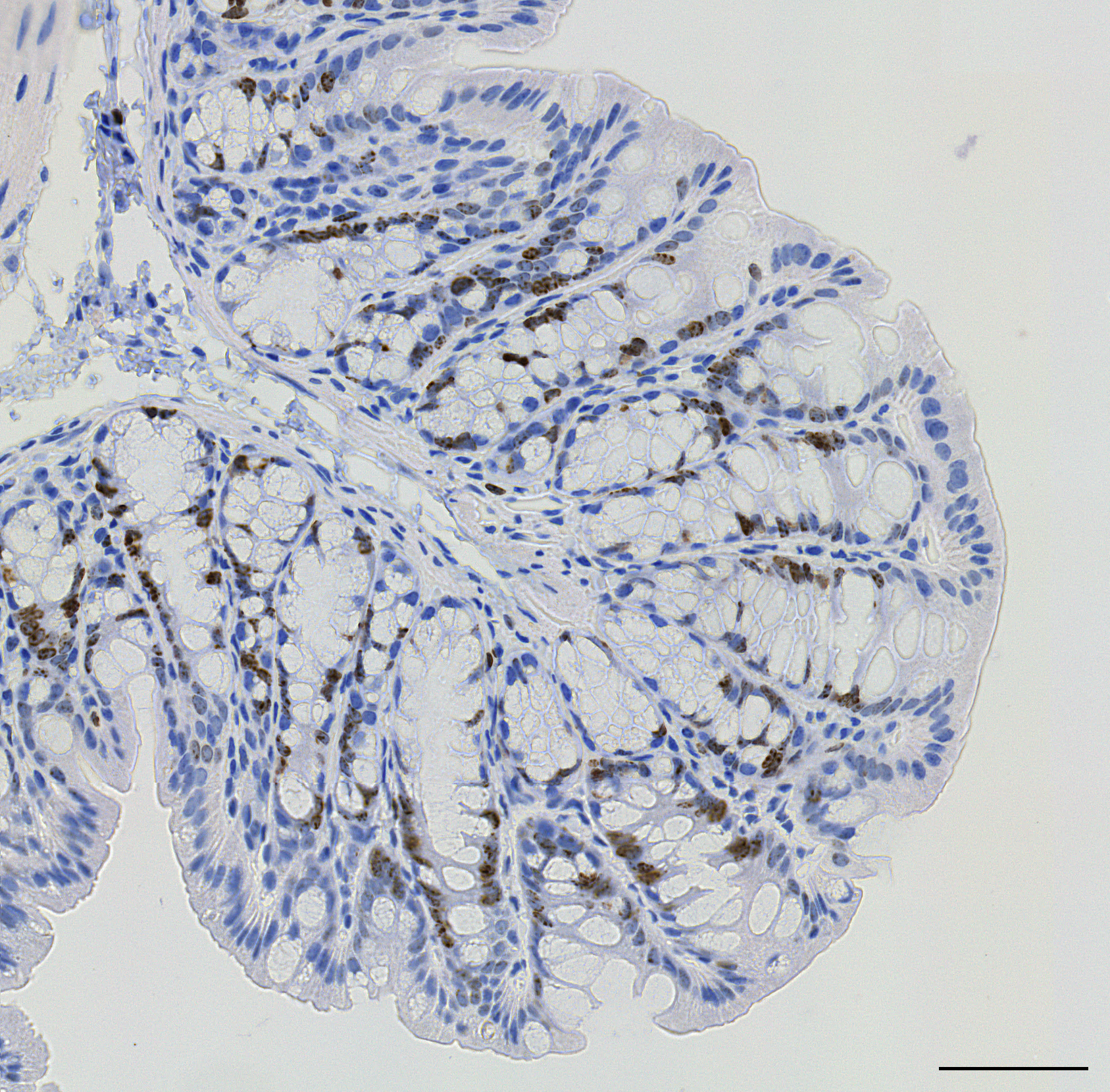

Supplement: Supplementary file 5 — Source data Fig. 2 [file 44321_2024_66_MOESM5_ESM.zip › Figure 2/2C/Yunhua-ki67-WT.tif]

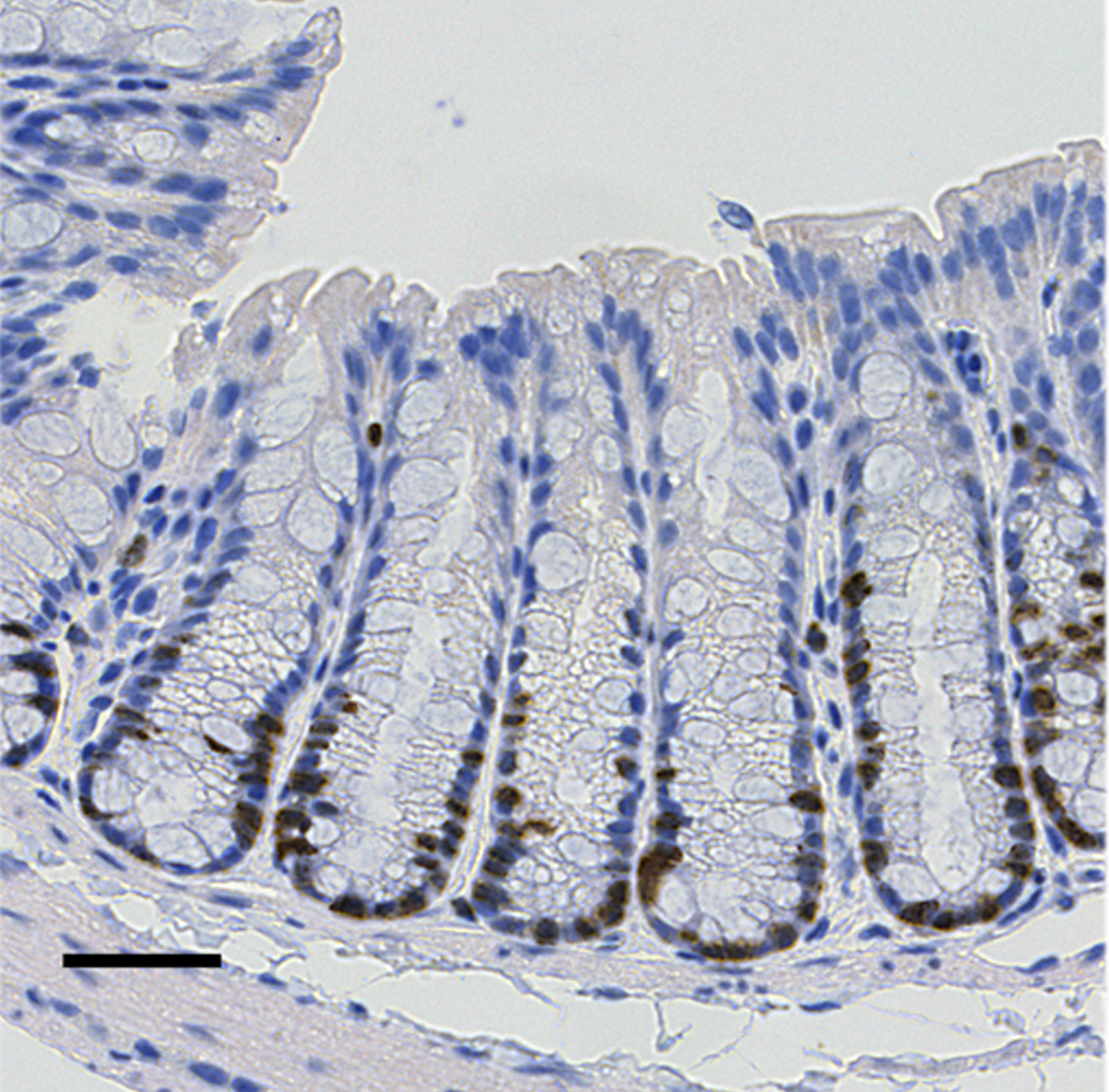

Supplement: Supplementary file 5 — Source data Fig. 2 [file 44321_2024_66_MOESM5_ESM.zip › Figure 2/2C/Yunhua-ki67-WT1.tif]

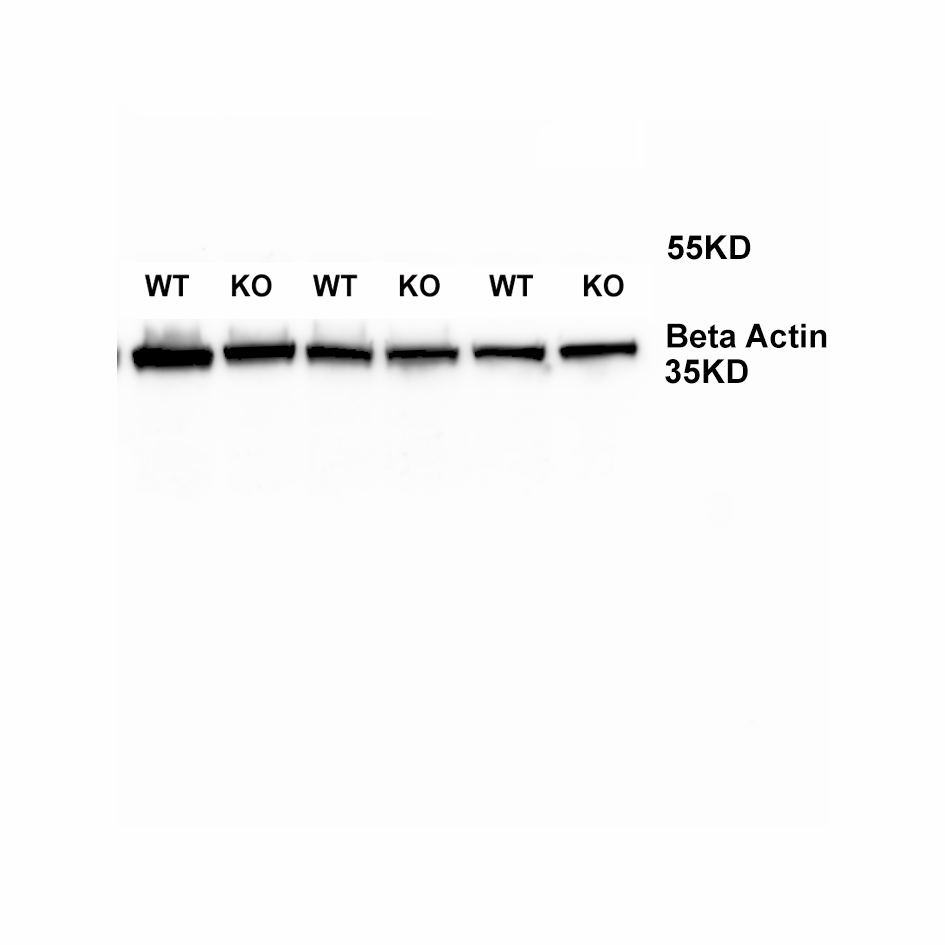

Supplement: Supplementary file 6 — Source data Fig. 4 [file 44321_2024_66_MOESM6_ESM.zip › Figure 4/4A/7days mice beta actin_09(Chemiluminescence).tif]

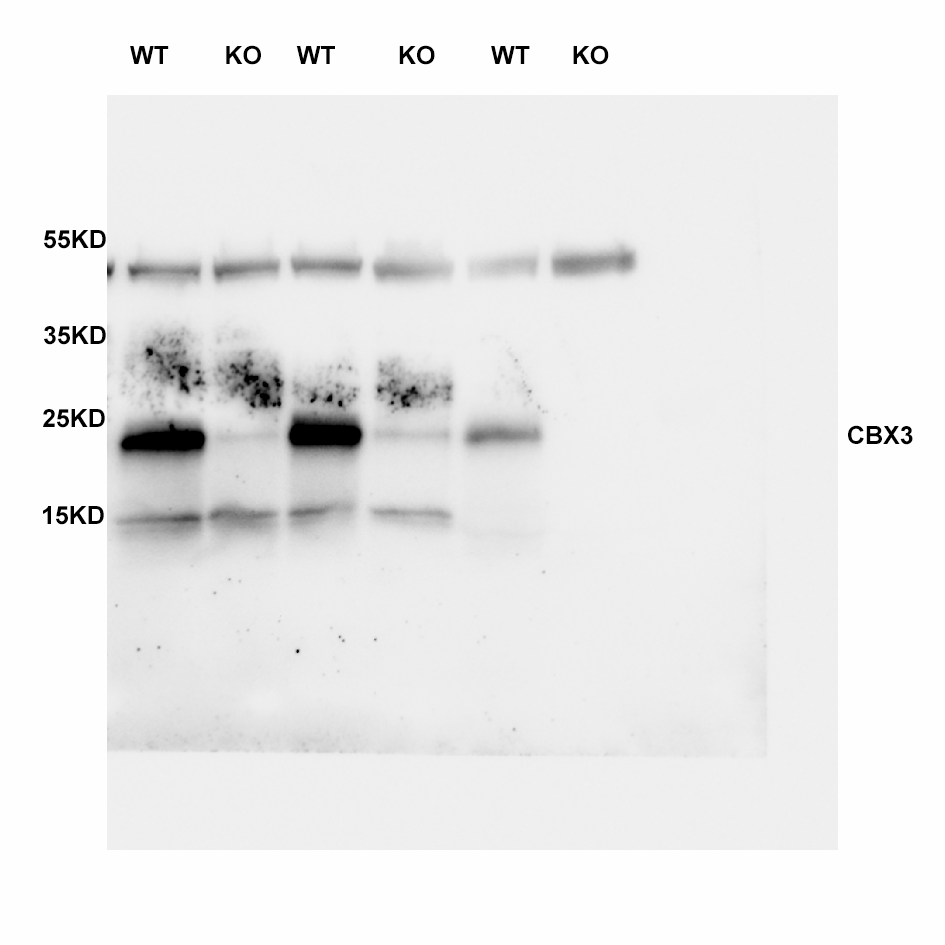

Supplement: Supplementary file 6 — Source data Fig. 4 [file 44321_2024_66_MOESM6_ESM.zip › Figure 4/4A/CBX3 7 days mice sample_07(Chemiluminescence).tif]

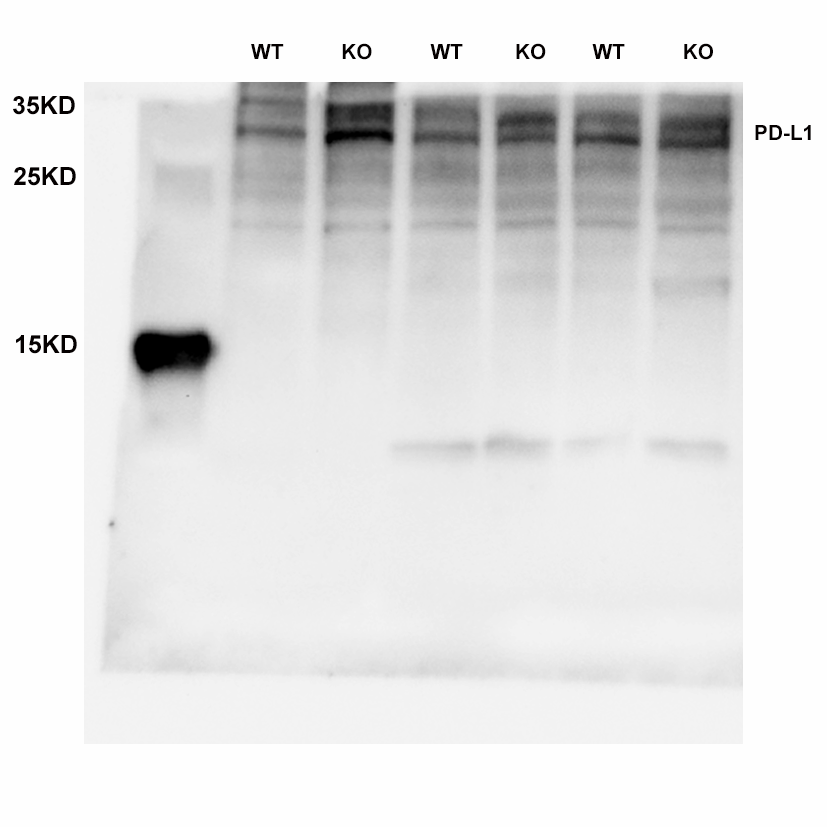

Supplement: Supplementary file 6 — Source data Fig. 4 [file 44321_2024_66_MOESM6_ESM.zip › Figure 4/4A/PD-L1 7 day mice 210305(Chemiluminescence).tif]

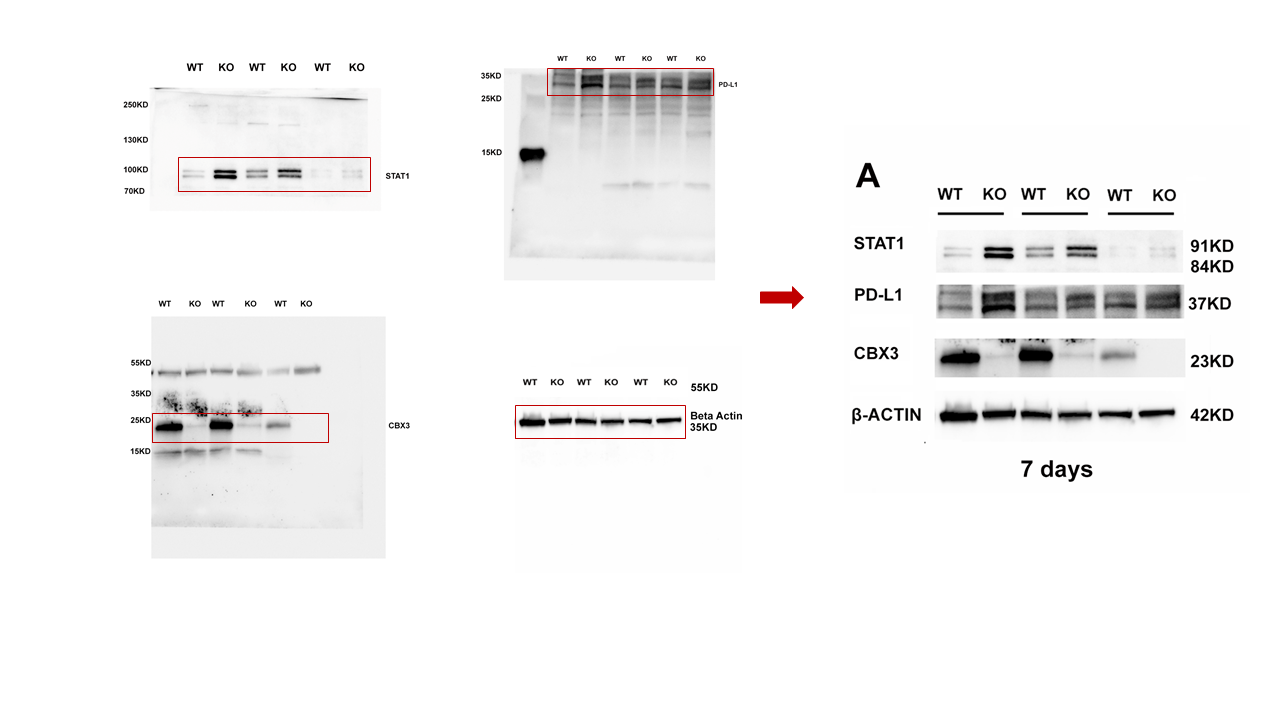

Supplement: Supplementary file 6 — Source data Fig. 4 [file 44321_2024_66_MOESM6_ESM.zip › Figure 4/4A/resume.tif]

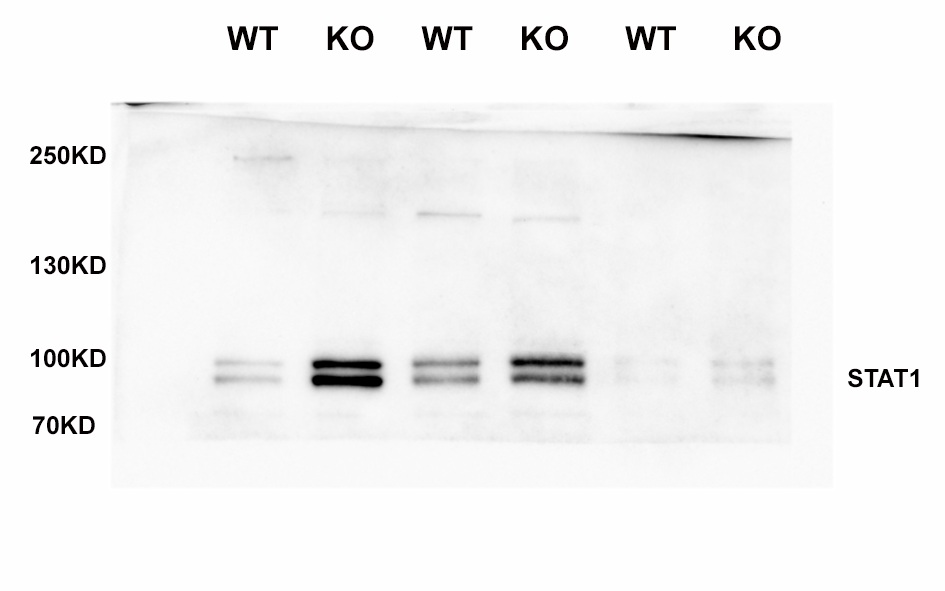

Supplement: Supplementary file 6 — Source data Fig. 4 [file 44321_2024_66_MOESM6_ESM.zip › Figure 4/4A/STAT1 7 day mice 210305(Chemiluminescence).tif]

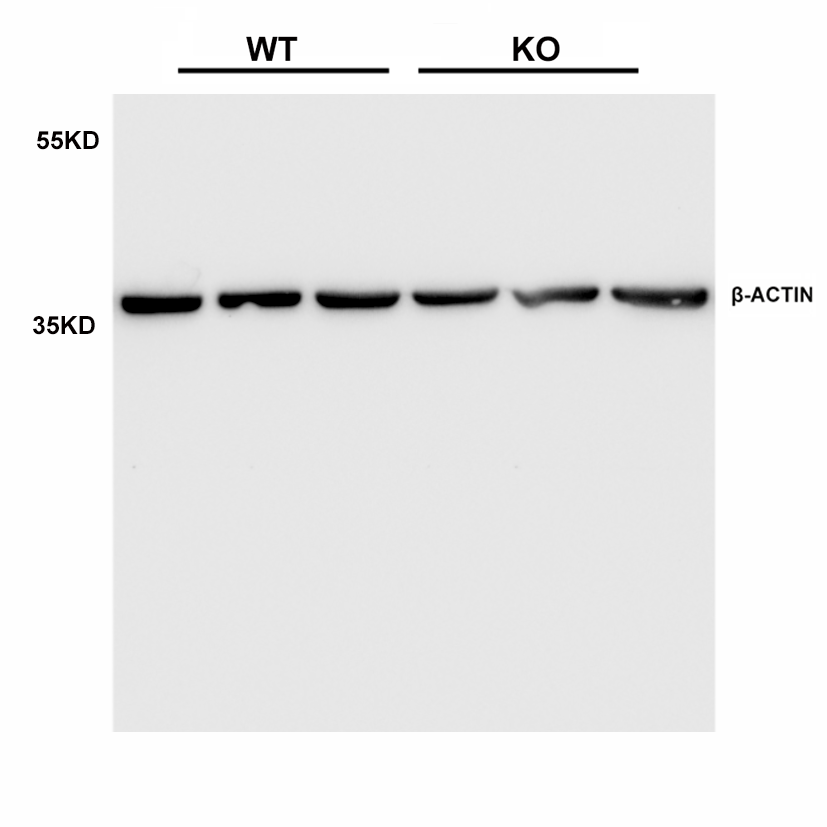

Supplement: Supplementary file 6 — Source data Fig. 4 [file 44321_2024_66_MOESM6_ESM.zip › Figure 4/4B/beta actin12 months mice sample_1(Chemiluminescence).tif]

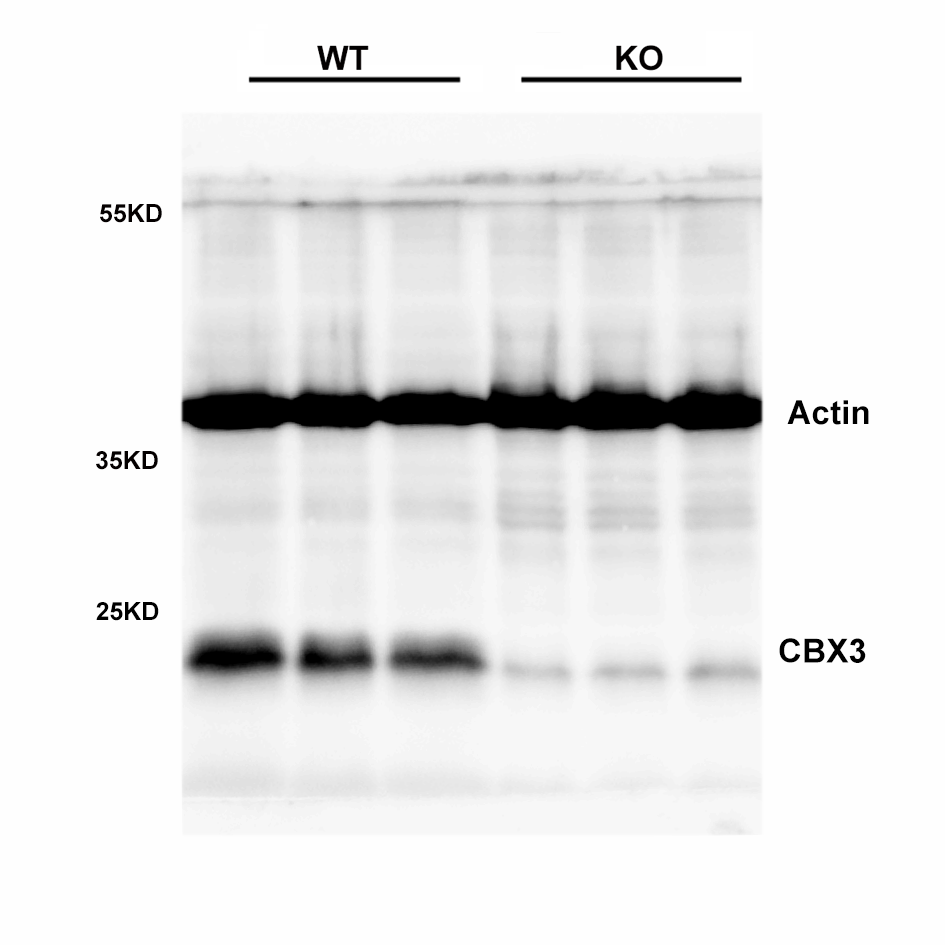

Supplement: Supplementary file 6 — Source data Fig. 4 [file 44321_2024_66_MOESM6_ESM.zip › Figure 4/4B/CBX3 12 months mice (Chemiluminescence).tif]

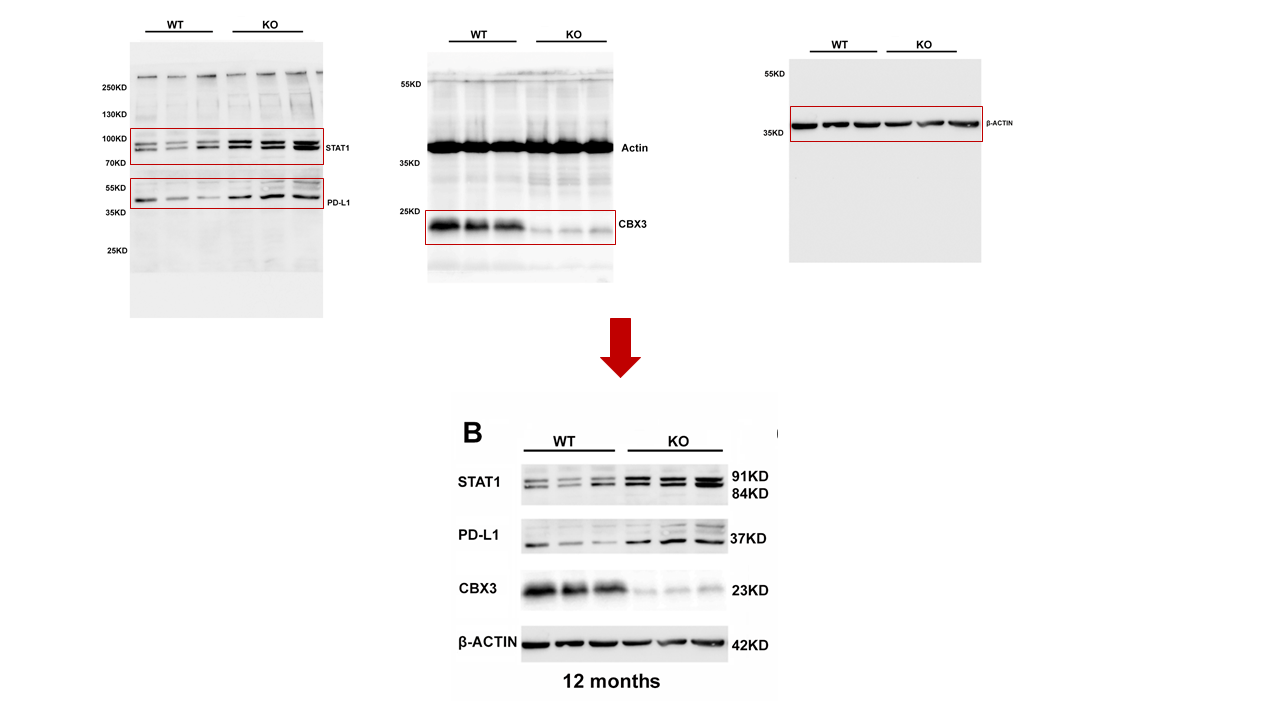

Supplement: Supplementary file 6 — Source data Fig. 4 [file 44321_2024_66_MOESM6_ESM.zip › Figure 4/4B/resume.tif]

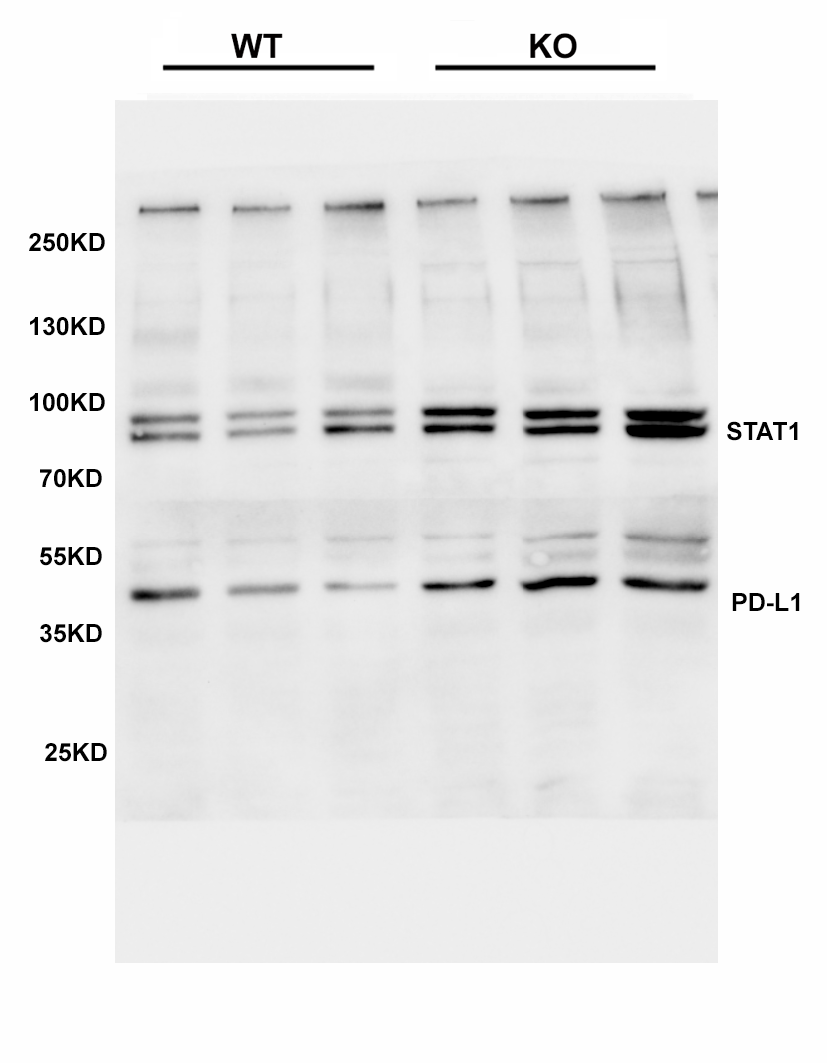

Supplement: Supplementary file 6 — Source data Fig. 4 [file 44321_2024_66_MOESM6_ESM.zip › Figure 4/4B/STAT1 PD-L1 12 months mice 210305(Chemiluminescence).tif]

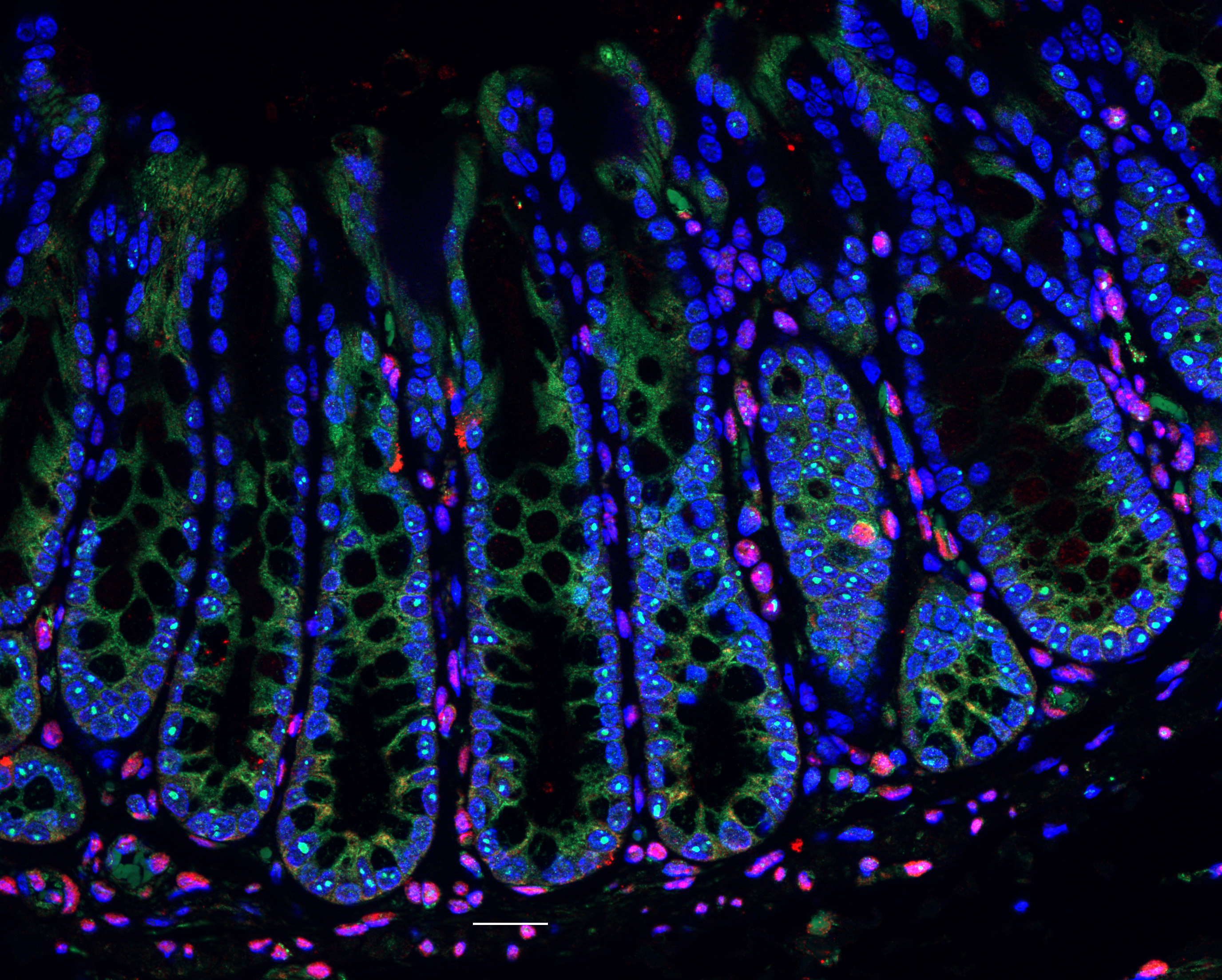

Supplement: Supplementary file 6 — Source data Fig. 4 [file 44321_2024_66_MOESM6_ESM.zip › Figure 4/4D/KO Nov20009colon3.tif_files/Images in paper/Nov20009colon3-Bar.tif]

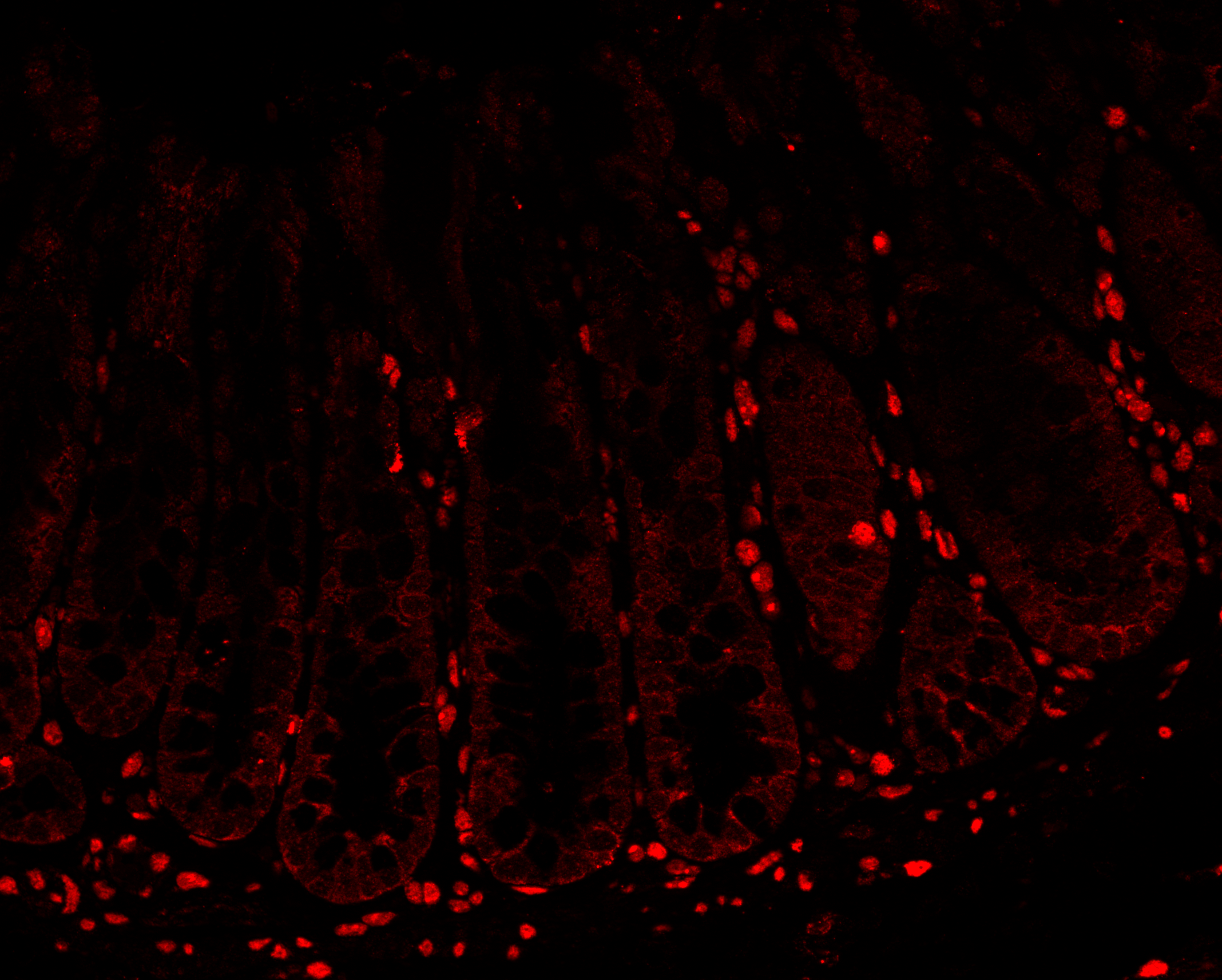

Supplement: Supplementary file 6 — Source data Fig. 4 [file 44321_2024_66_MOESM6_ESM.zip › Figure 4/4D/KO Nov20009colon3.tif_files/Images in paper/redNov20009colon3.tif]

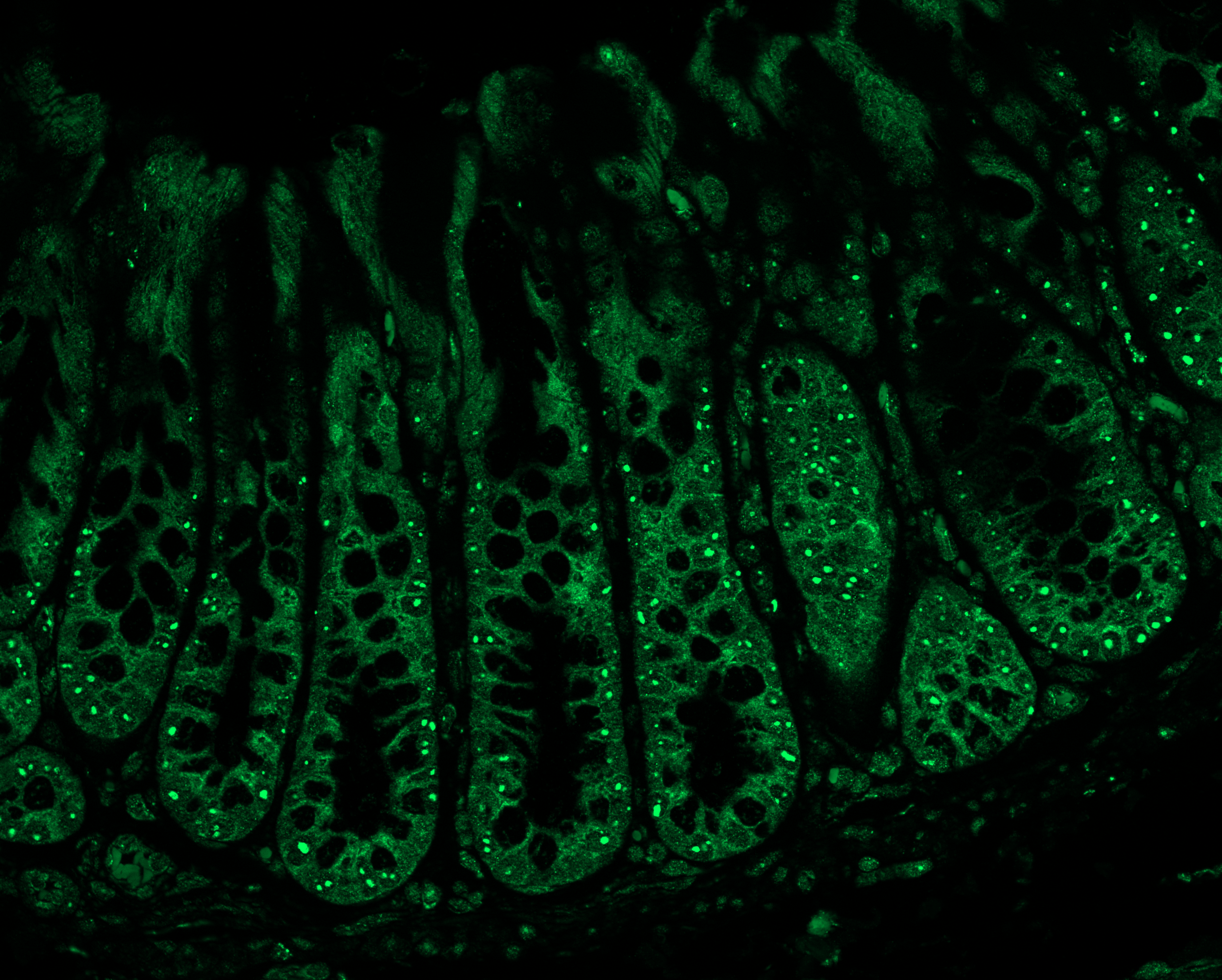

Supplement: Supplementary file 6 — Source data Fig. 4 [file 44321_2024_66_MOESM6_ESM.zip › Figure 4/4D/KO Nov20009colon3.tif_files/Images in paper/vertNov20009colon3.tif]

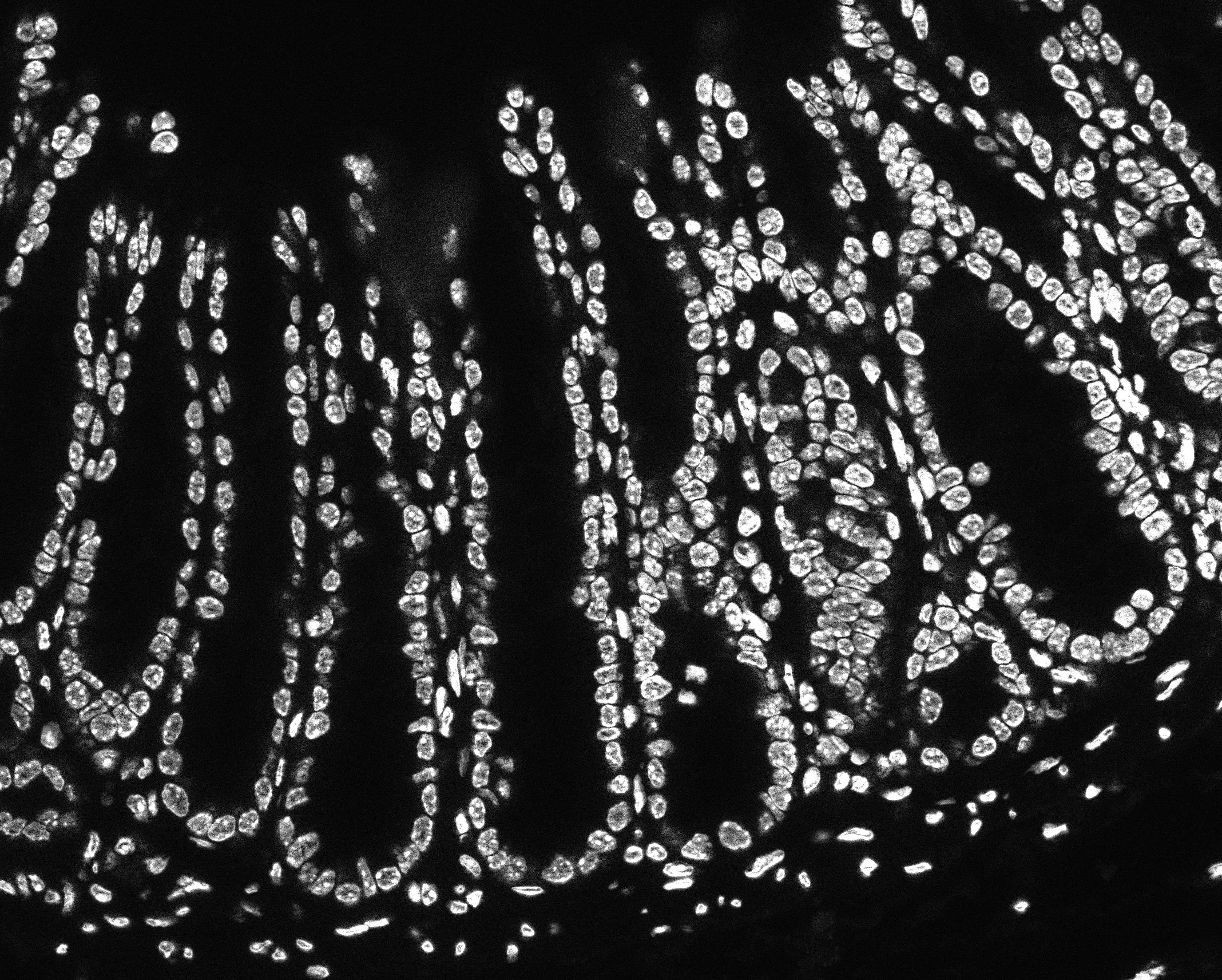

Supplement: Supplementary file 6 — Source data Fig. 4 [file 44321_2024_66_MOESM6_ESM.zip › Figure 4/4D/KO Nov20009colon3.tif_files/Nov20009colon3_h0b0c0x0-2752y0-2208.tif]

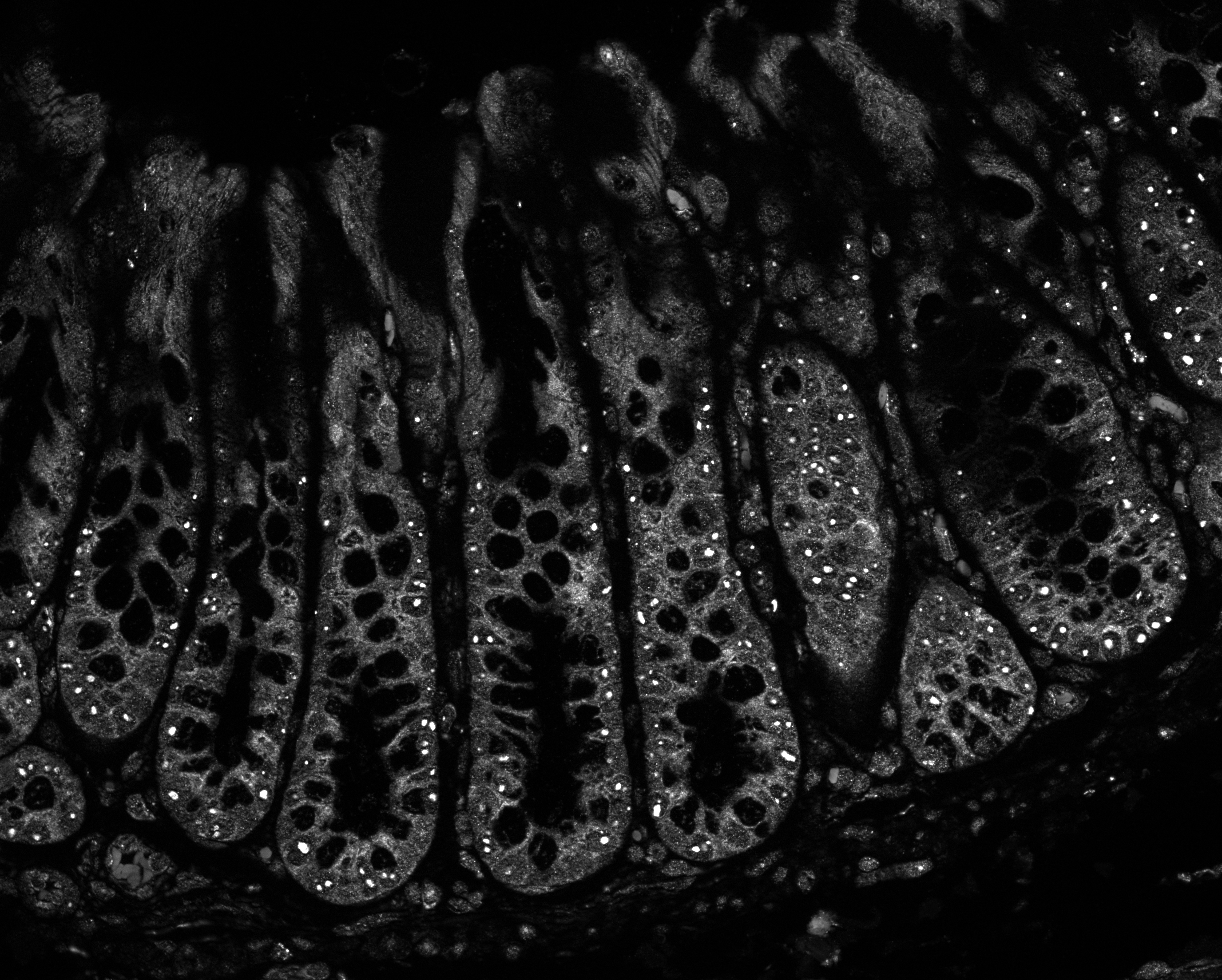

Supplement: Supplementary file 6 — Source data Fig. 4 [file 44321_2024_66_MOESM6_ESM.zip › Figure 4/4D/KO Nov20009colon3.tif_files/Nov20009colon3_h0b0c1x0-2752y0-2208.tif]

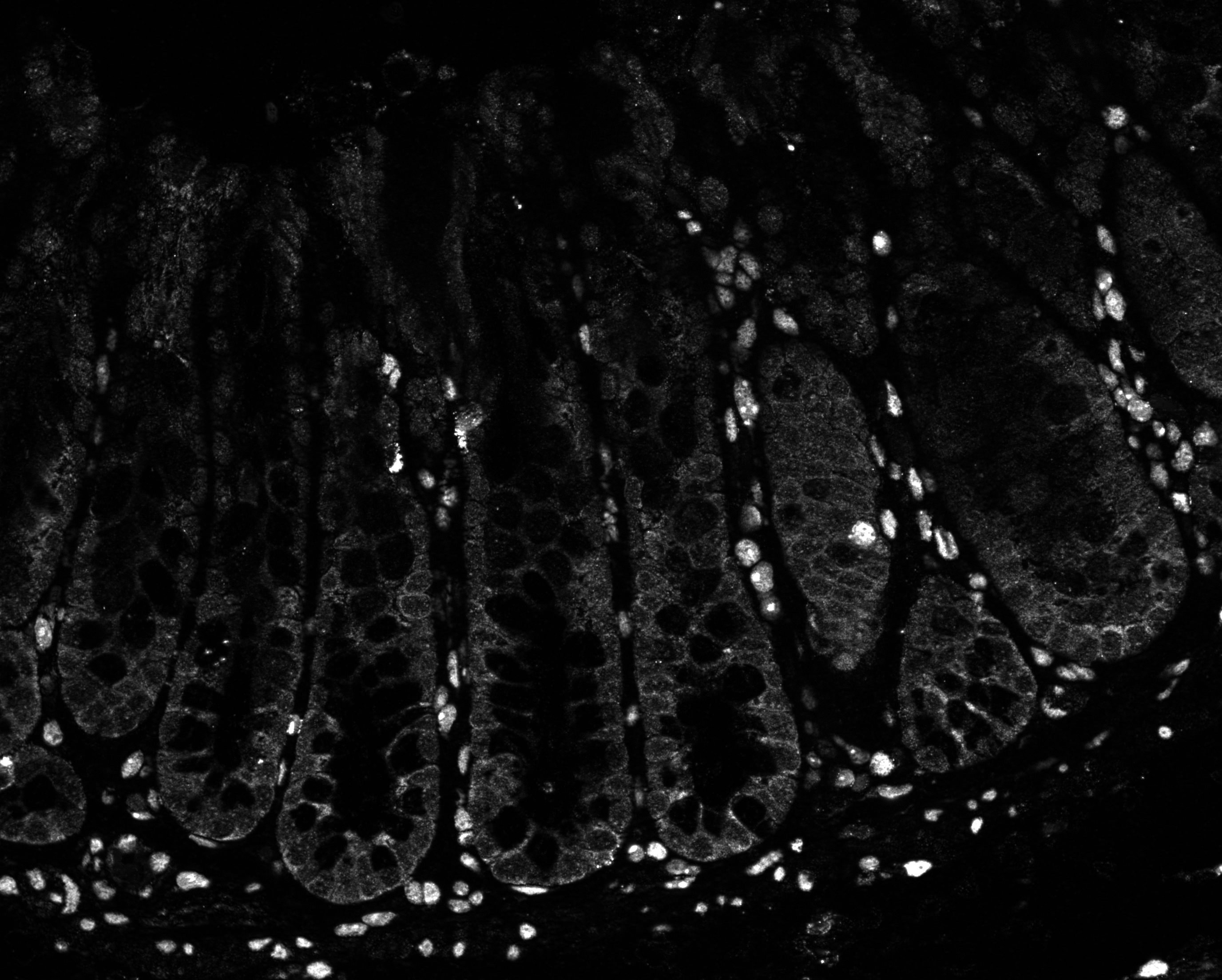

Supplement: Supplementary file 6 — Source data Fig. 4 [file 44321_2024_66_MOESM6_ESM.zip › Figure 4/4D/KO Nov20009colon3.tif_files/Nov20009colon3_h0b0c2x0-2752y0-2208.tif]

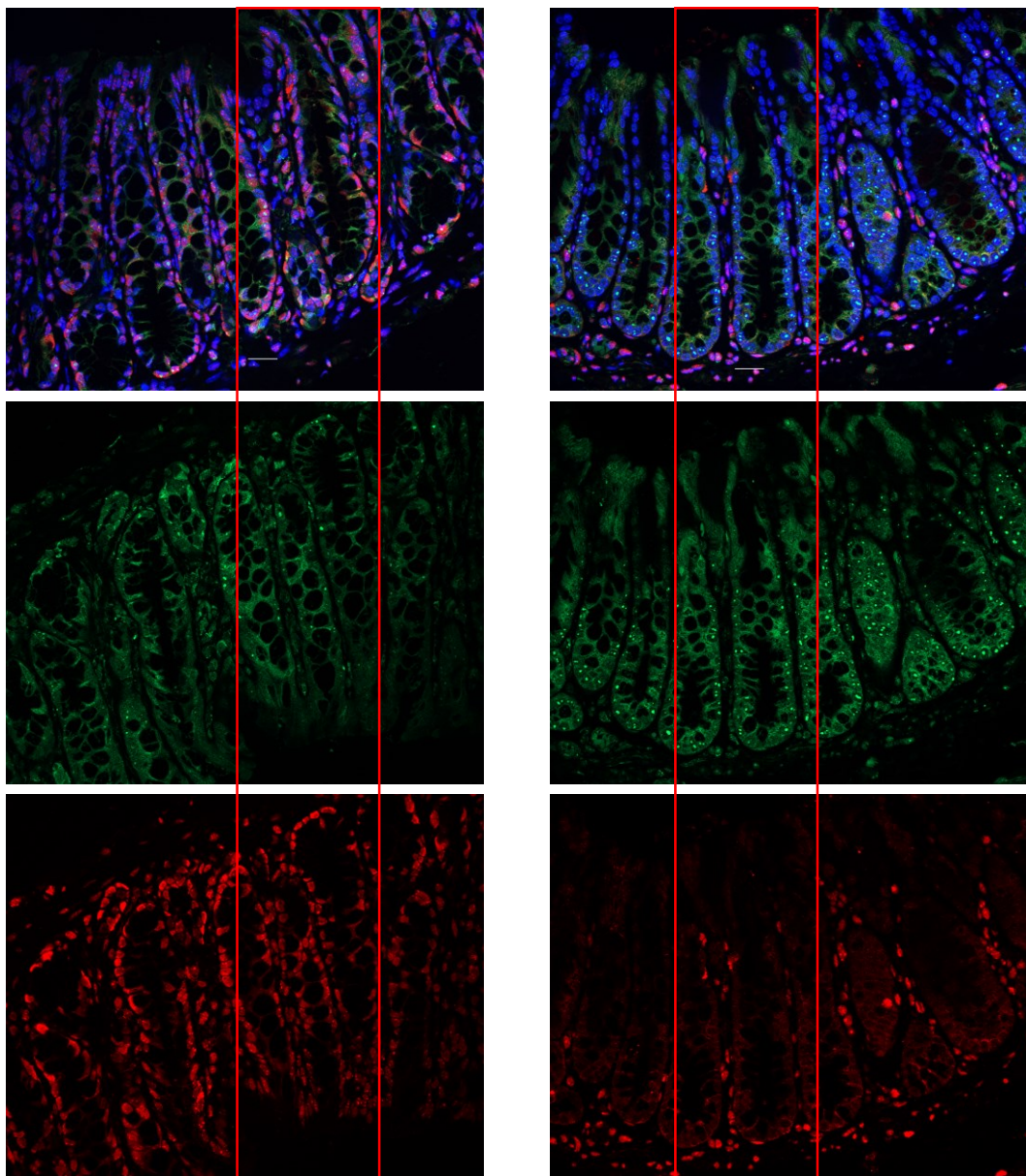

**D**

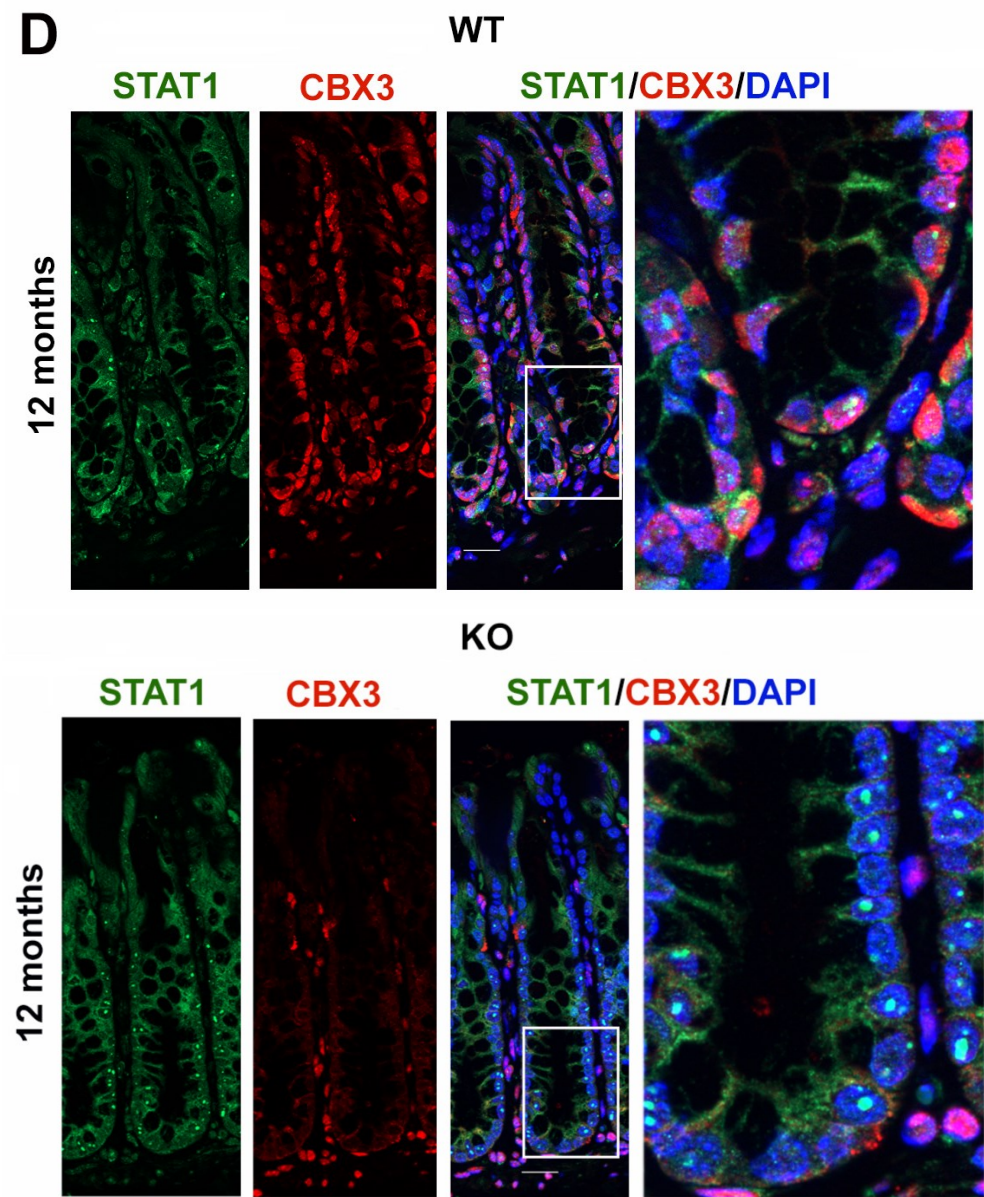

Supplement: Supplementary file 6 — Source data Fig. 4 [file 44321_2024_66_MOESM6_ESM.zip › Figure 4/4D/resume.pdf]

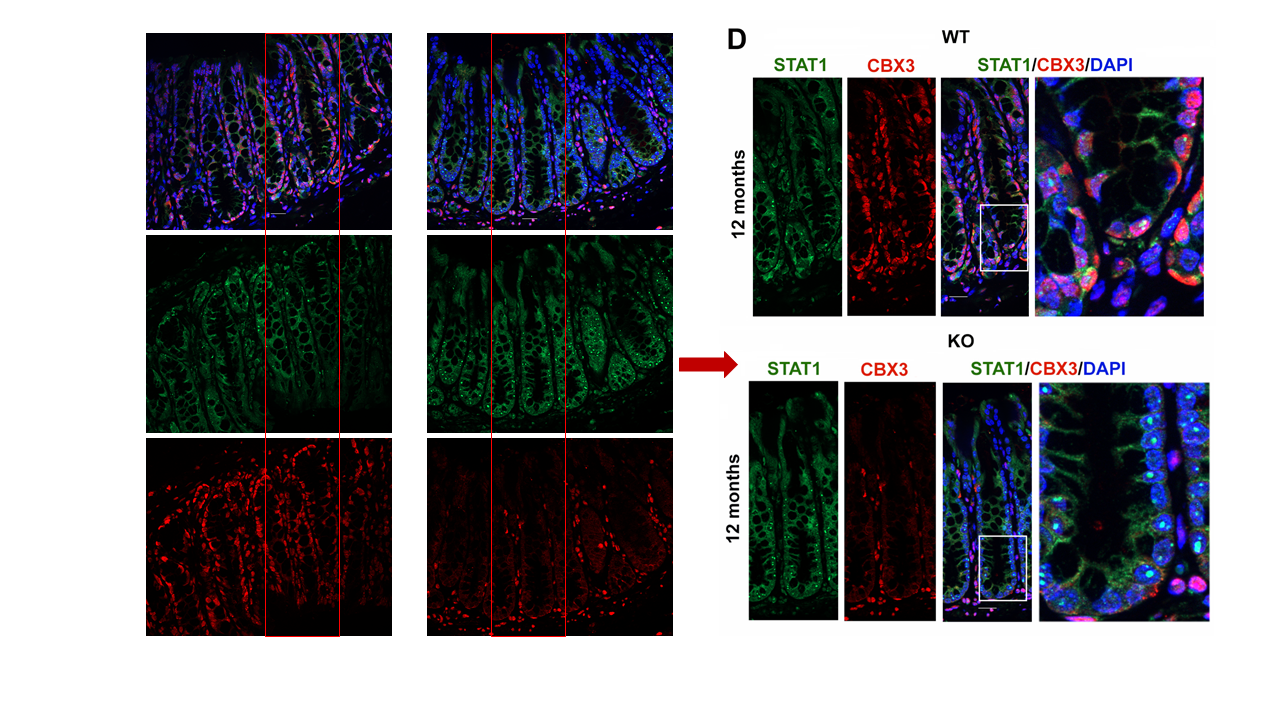

Supplement: Supplementary file 6 — Source data Fig. 4 [file 44321_2024_66_MOESM6_ESM.zip › Figure 4/4D/resume.tif]

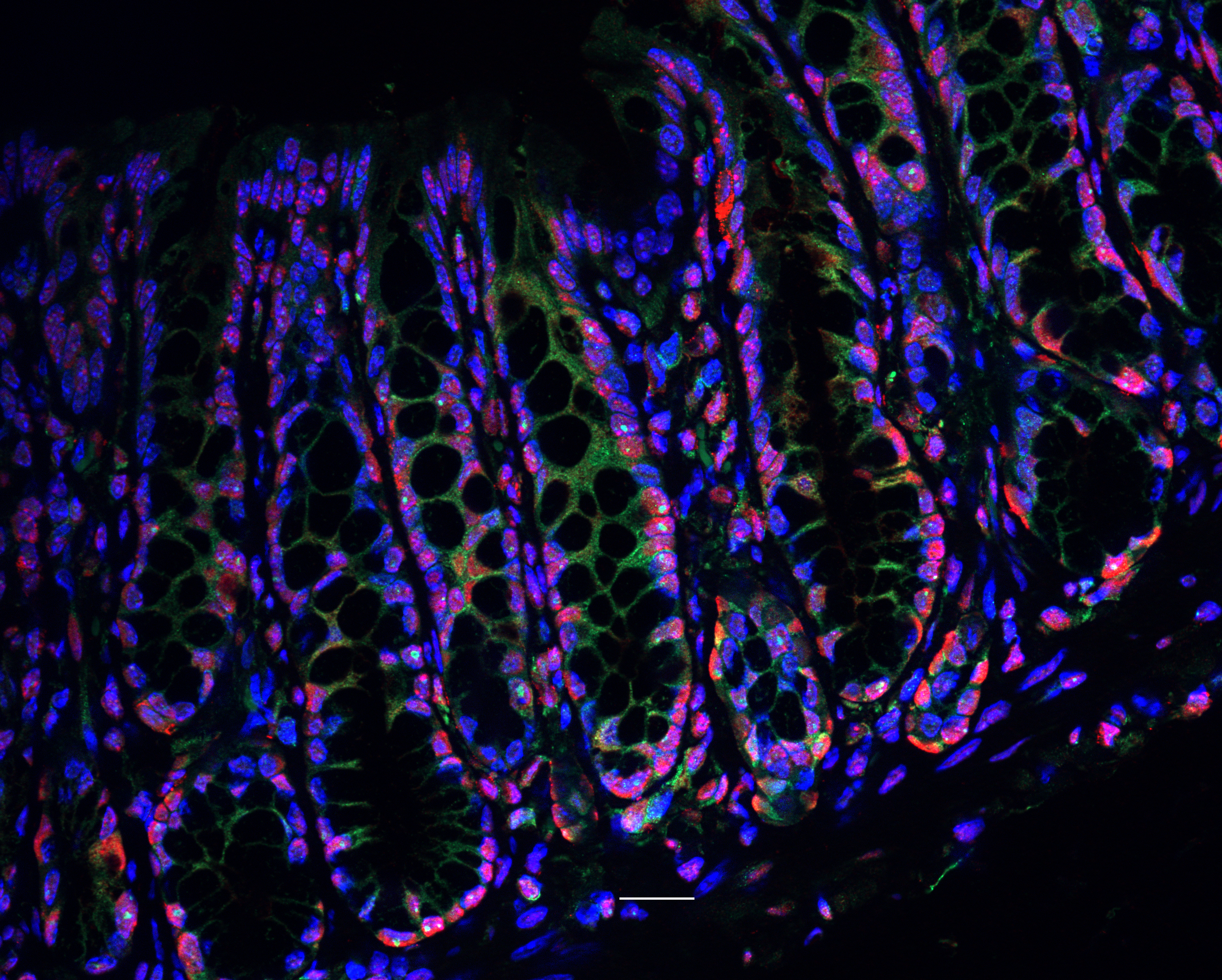

Supplement: Supplementary file 6 — Source data Fig. 4 [file 44321_2024_66_MOESM6_ESM.zip › Figure 4/4D/WT Nov20013colon3.tif_files/images in paper/Nov20013colon3-Bar.tif]

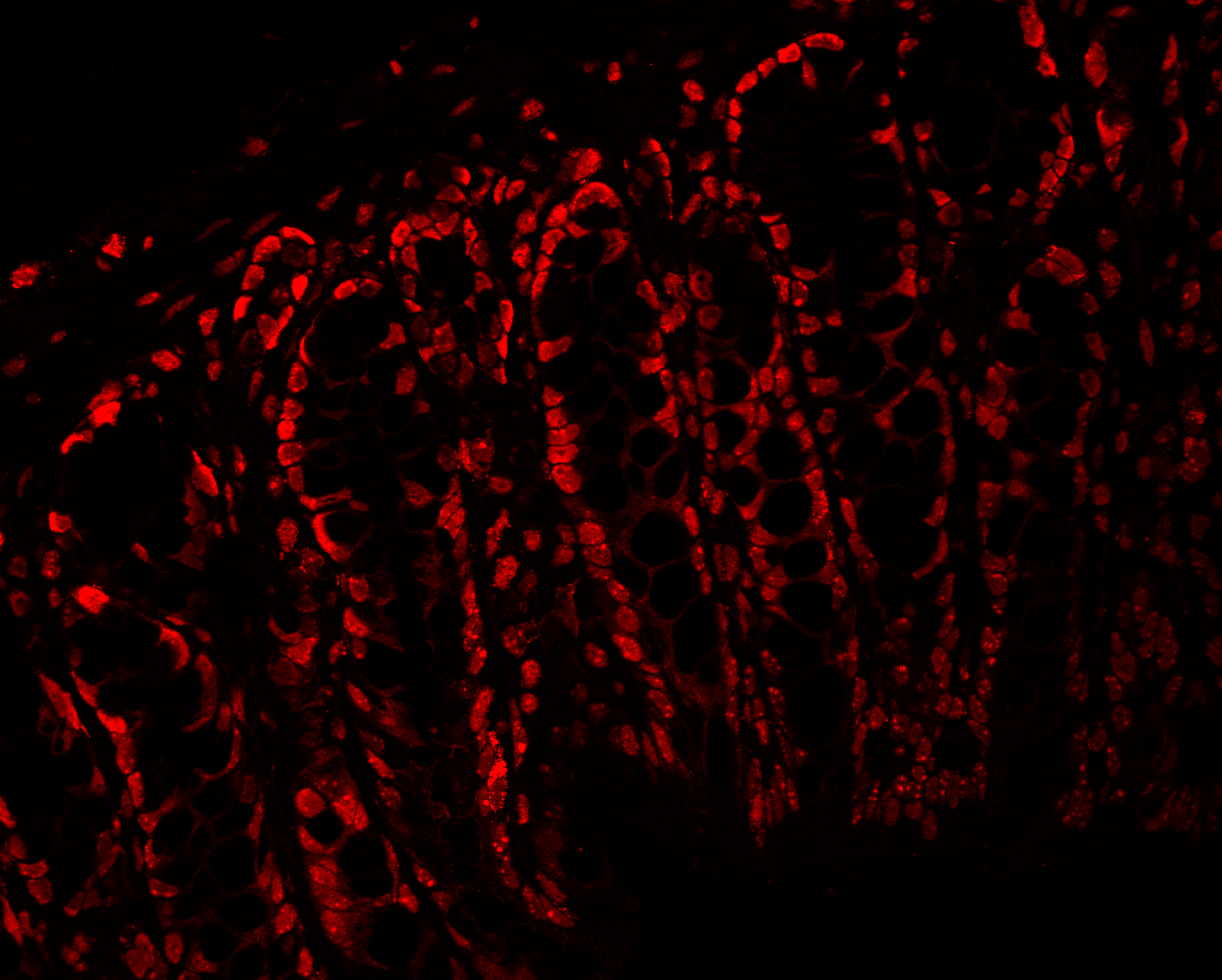

Supplement: Supplementary file 6 — Source data Fig. 4 [file 44321_2024_66_MOESM6_ESM.zip › Figure 4/4D/WT Nov20013colon3.tif_files/images in paper/red Nov20013colon3.tif]

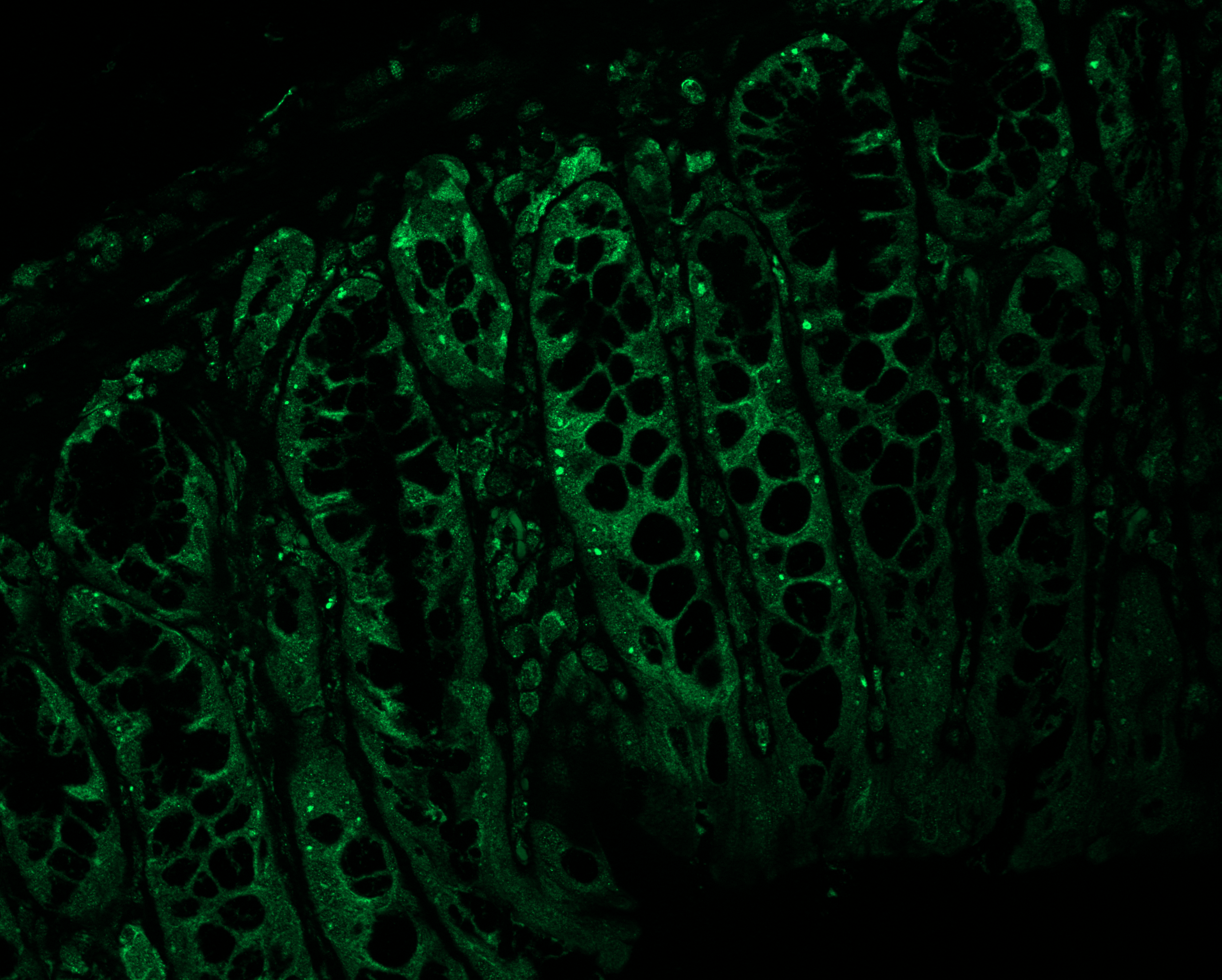

Supplement: Supplementary file 6 — Source data Fig. 4 [file 44321_2024_66_MOESM6_ESM.zip › Figure 4/4D/WT Nov20013colon3.tif_files/images in paper/vert Nov20013colon3.tif]

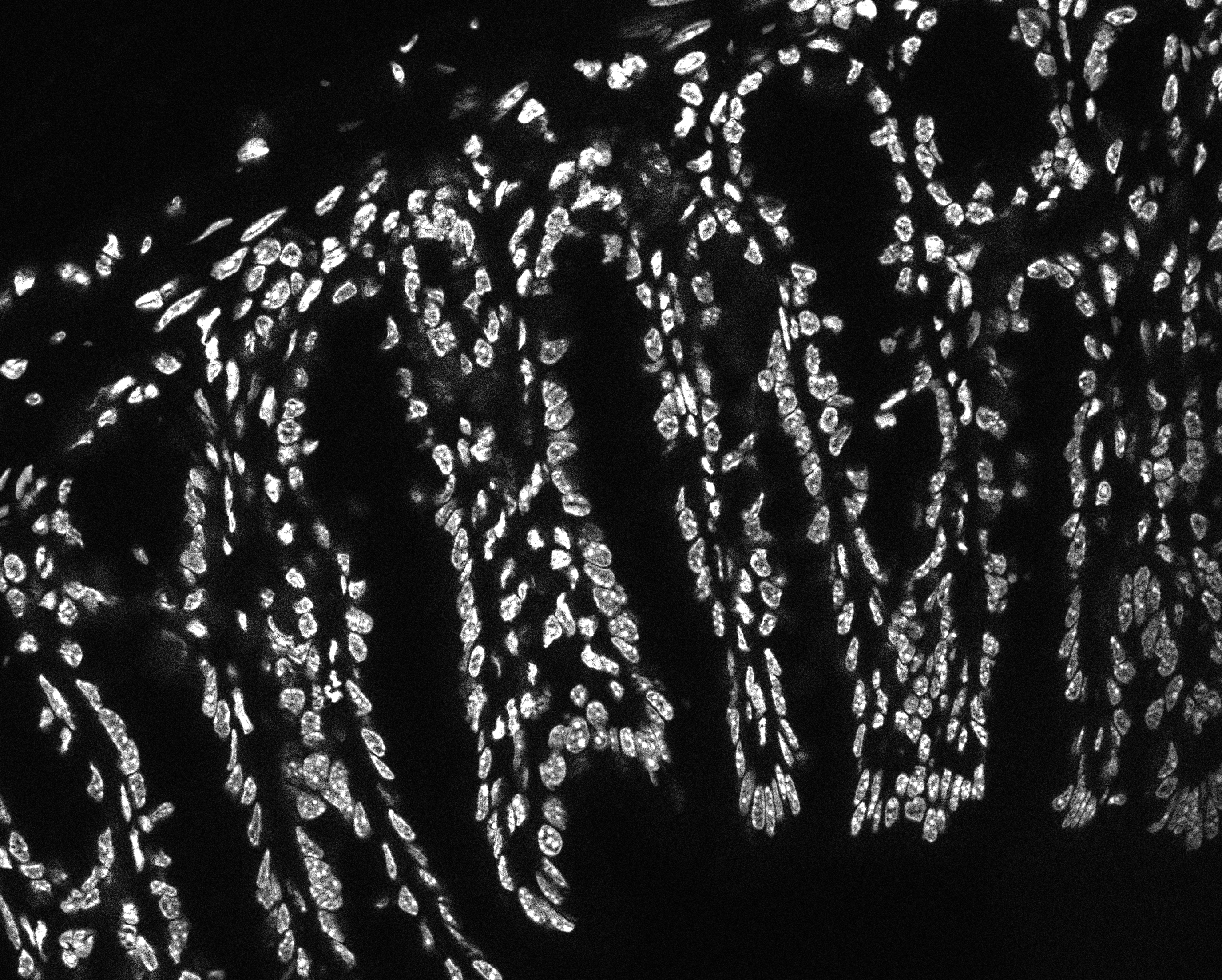

Supplement: Supplementary file 6 — Source data Fig. 4 [file 44321_2024_66_MOESM6_ESM.zip › Figure 4/4D/WT Nov20013colon3.tif_files/Nov20013colon3_h0b0t0c0x0-2752y0-2208.tif]

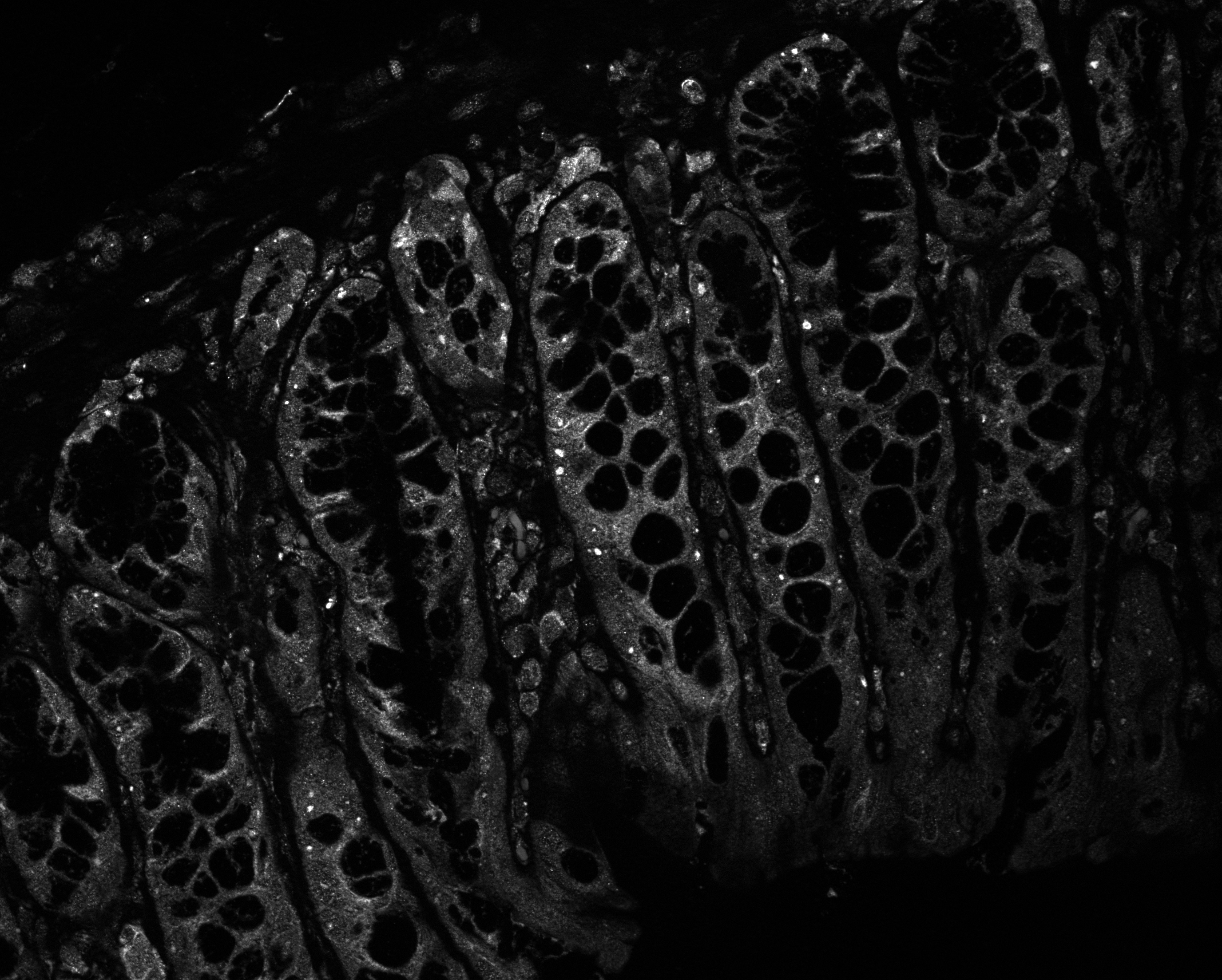

Supplement: Supplementary file 6 — Source data Fig. 4 [file 44321_2024_66_MOESM6_ESM.zip › Figure 4/4D/WT Nov20013colon3.tif_files/Nov20013colon3_h0b0t0c1x0-2752y0-2208.tif]

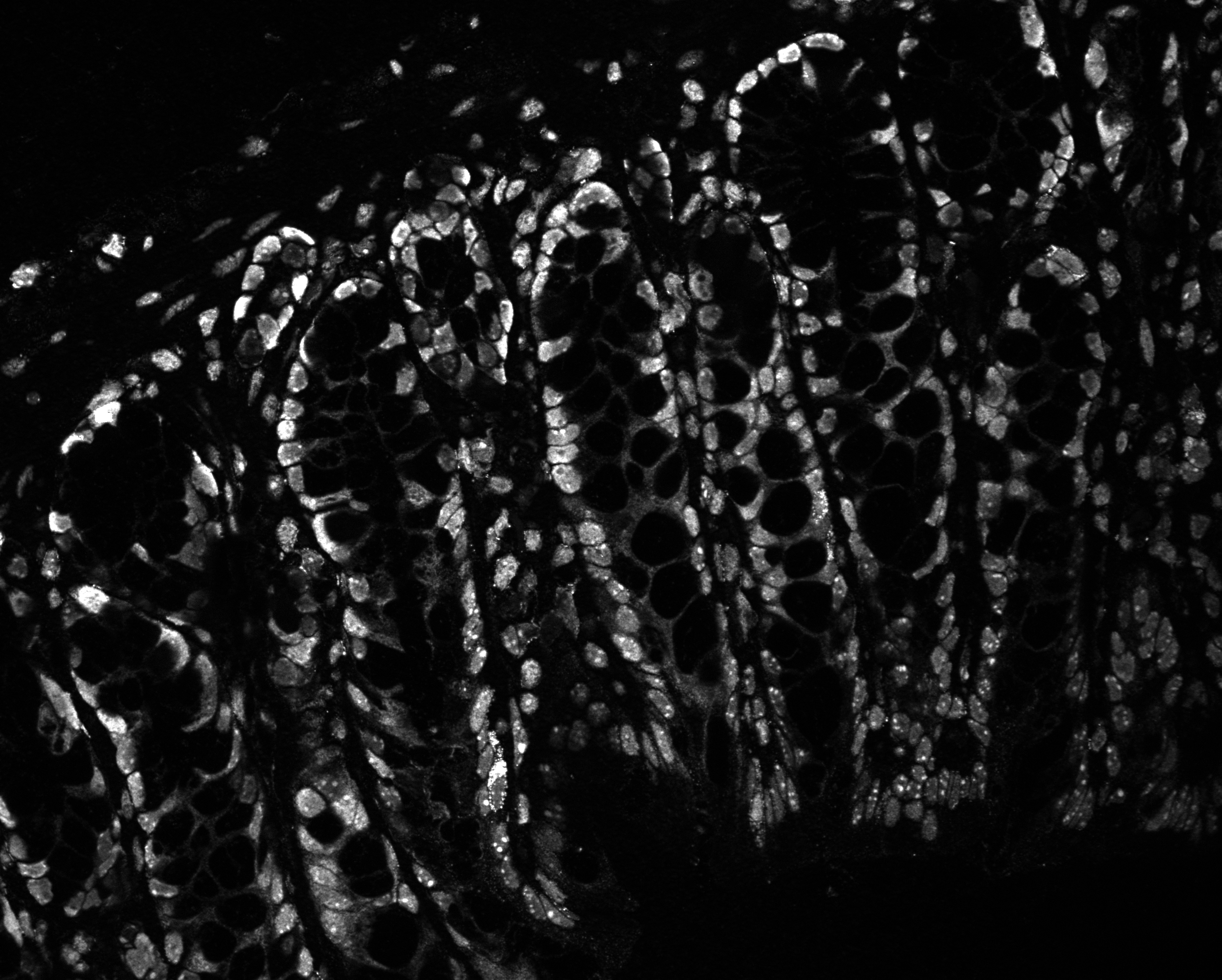

Supplement: Supplementary file 6 — Source data Fig. 4 [file 44321_2024_66_MOESM6_ESM.zip › Figure 4/4D/WT Nov20013colon3.tif_files/Nov20013colon3_h0b0t0c2x0-2752y0-2208.tif]

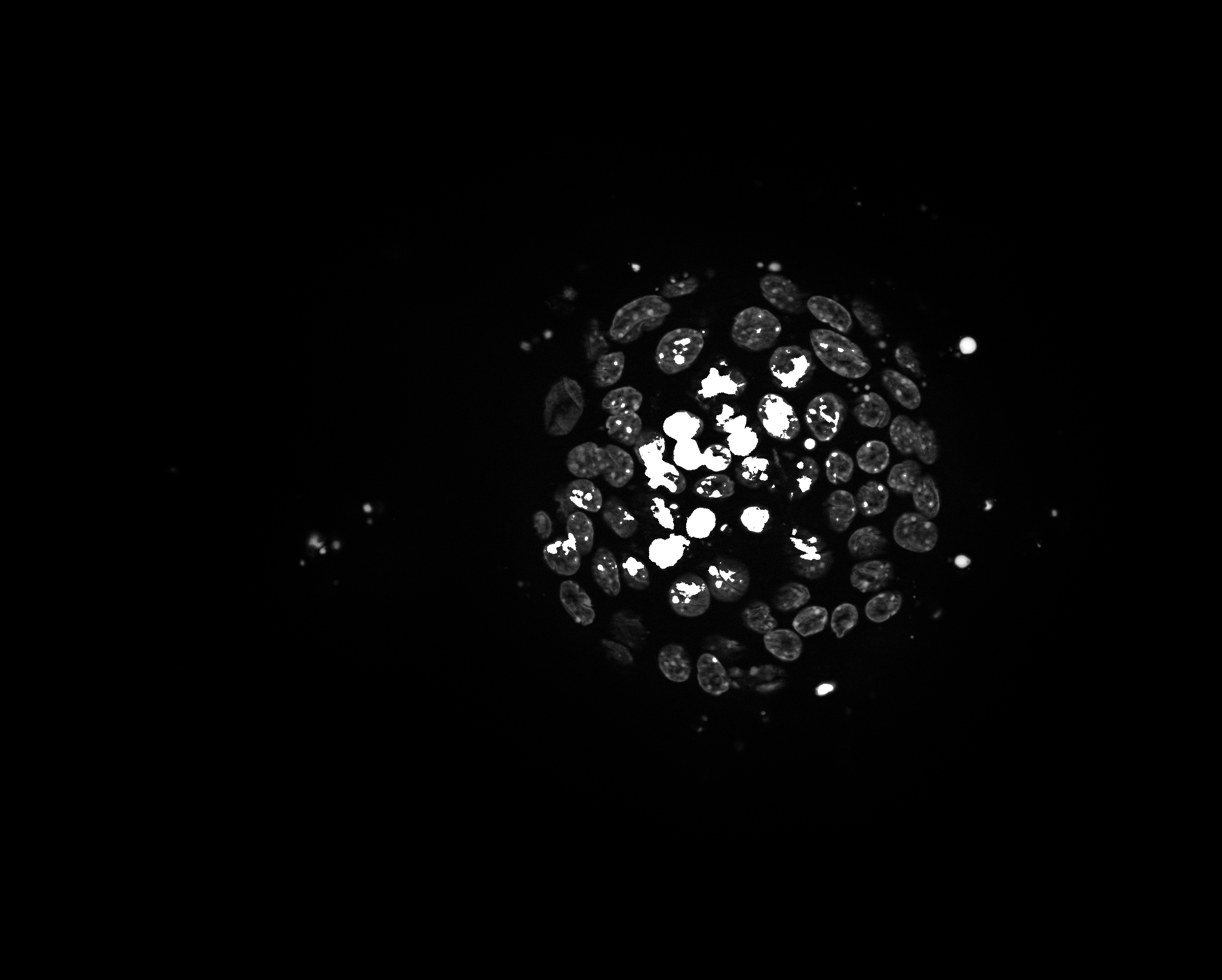

Supplement: Supplementary file 6 — Source data Fig. 4 [file 44321_2024_66_MOESM6_ESM.zip › Figure 4/4E/40X ZO1 RED KO COLON.tif_files/40X ZO1 RED HP1GAMMA KO COLON_h0b0c0x0-2752y0-2208.tif]

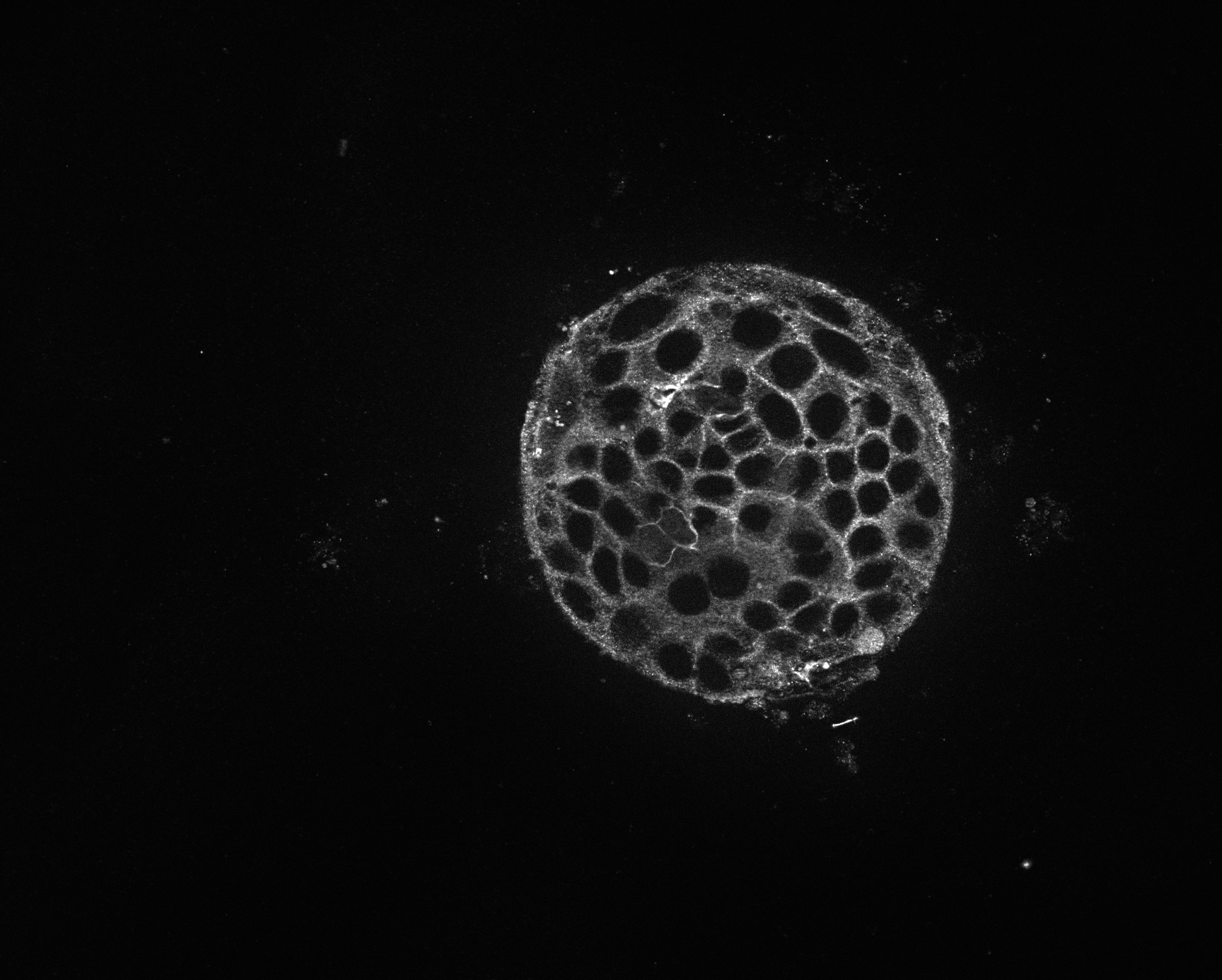

Supplement: Supplementary file 6 — Source data Fig. 4 [file 44321_2024_66_MOESM6_ESM.zip › Figure 4/4E/40X ZO1 RED KO COLON.tif_files/40X ZO1 RED HP1GAMMA KO COLON_h0b0c2x0-2752y0-2208.tif]

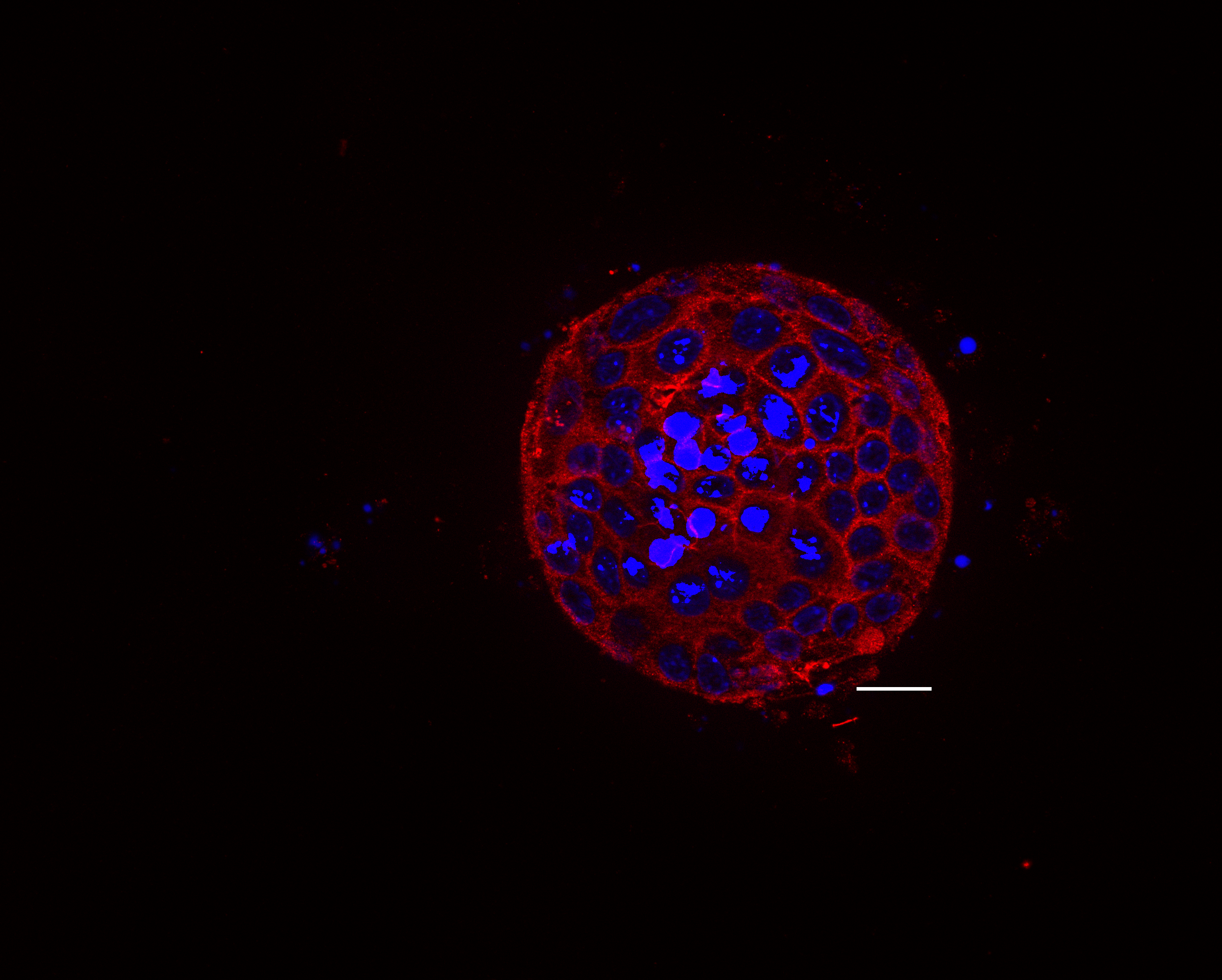

Supplement: Supplementary file 6 — Source data Fig. 4 [file 44321_2024_66_MOESM6_ESM.zip › Figure 4/4E/40X ZO1 RED KO COLON.tif_files/40X ZO1 RED MUC2 GREEN HP1GAMMA KO COLON-Bar.tif]

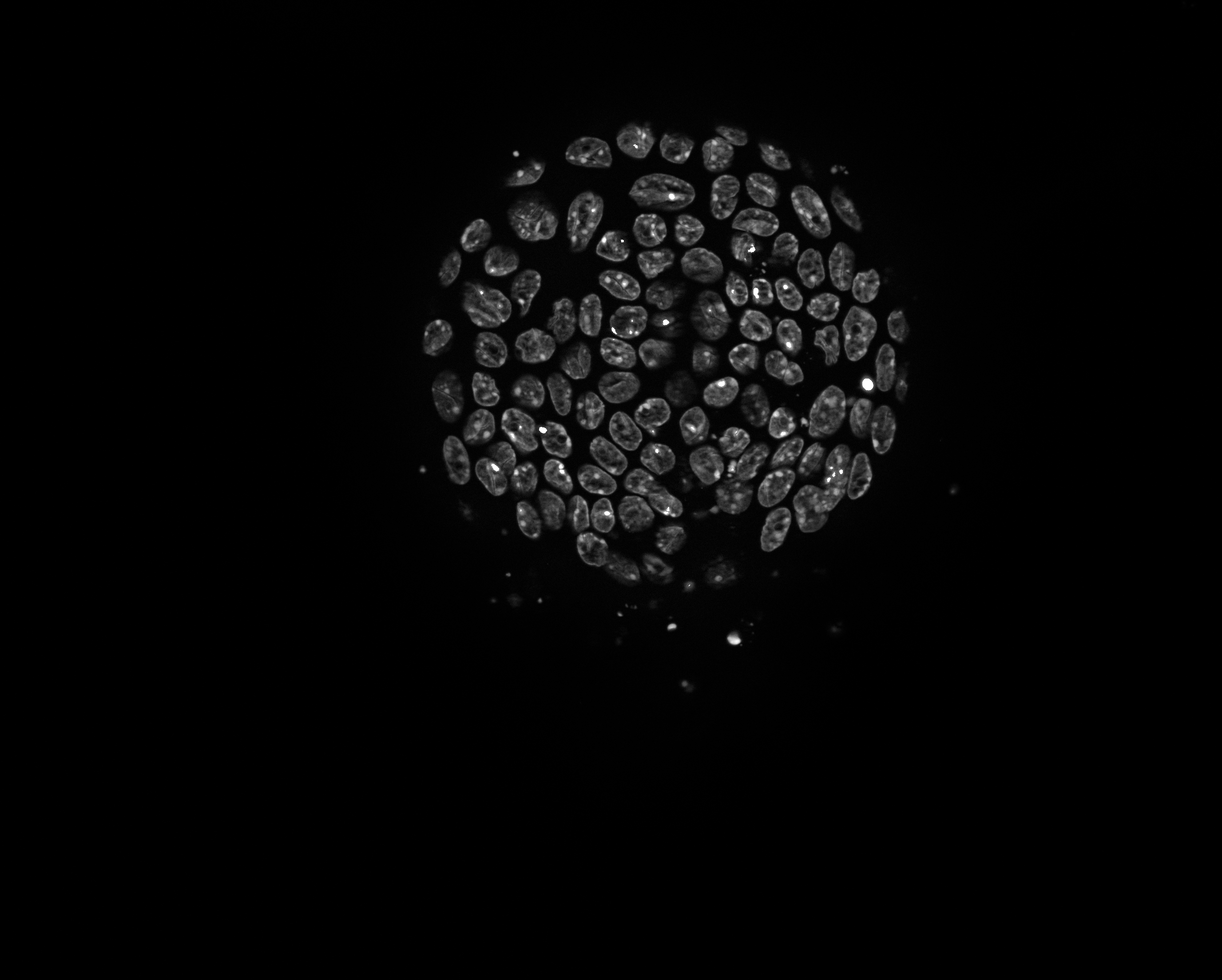

Supplement: Supplementary file 6 — Source data Fig. 4 [file 44321_2024_66_MOESM6_ESM.zip › Figure 4/4E/40X ZO1 RED WT COLON.tif_files/40X ZO1 RED MUC2 GREEN WT COLON_h0b0c0x0-2752y0-2208.tif]

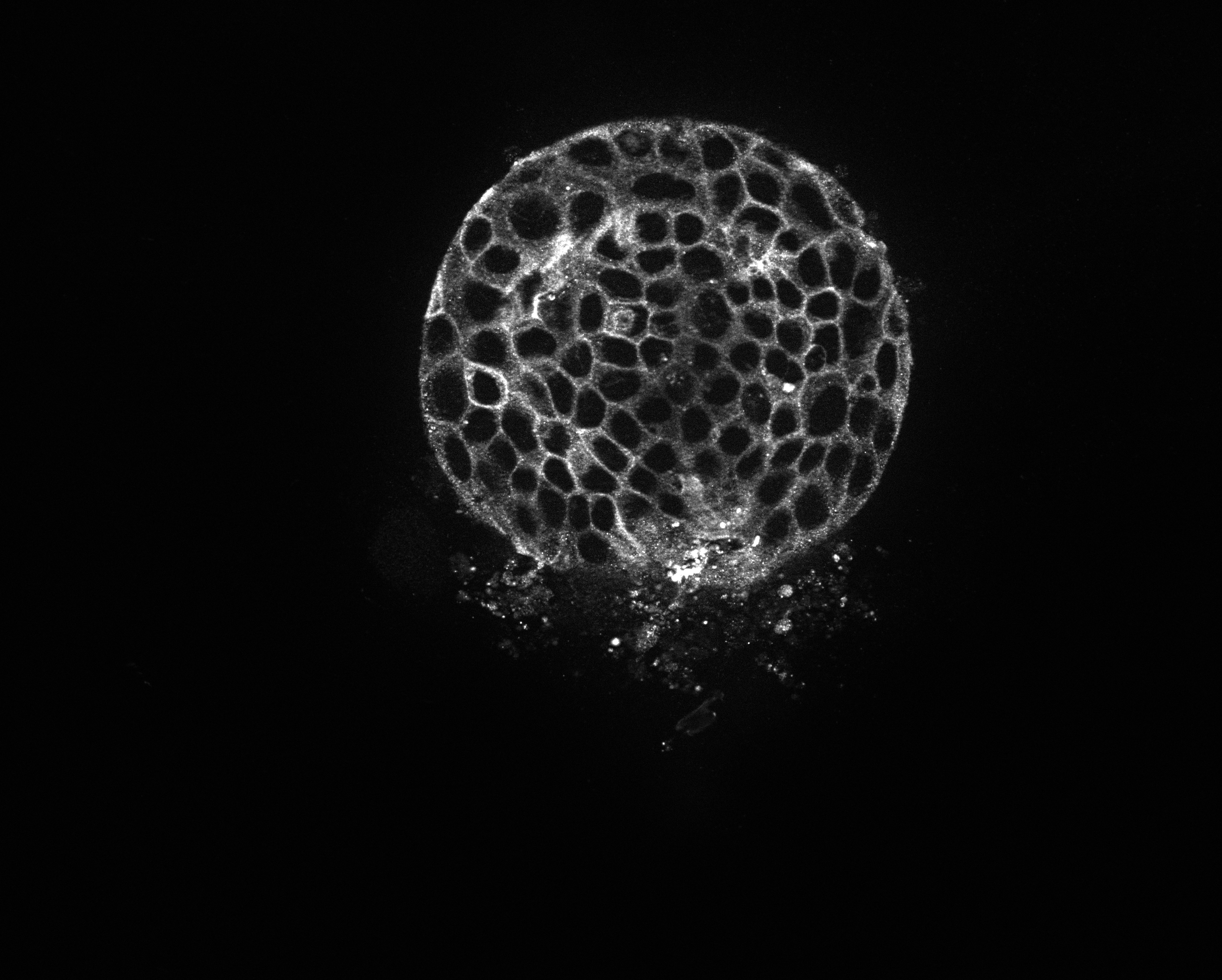

Supplement: Supplementary file 6 — Source data Fig. 4 [file 44321_2024_66_MOESM6_ESM.zip › Figure 4/4E/40X ZO1 RED WT COLON.tif_files/40X ZO1 RED MUC2 GREEN WT COLON_h0b0c3x0-2752y0-2208.tif]

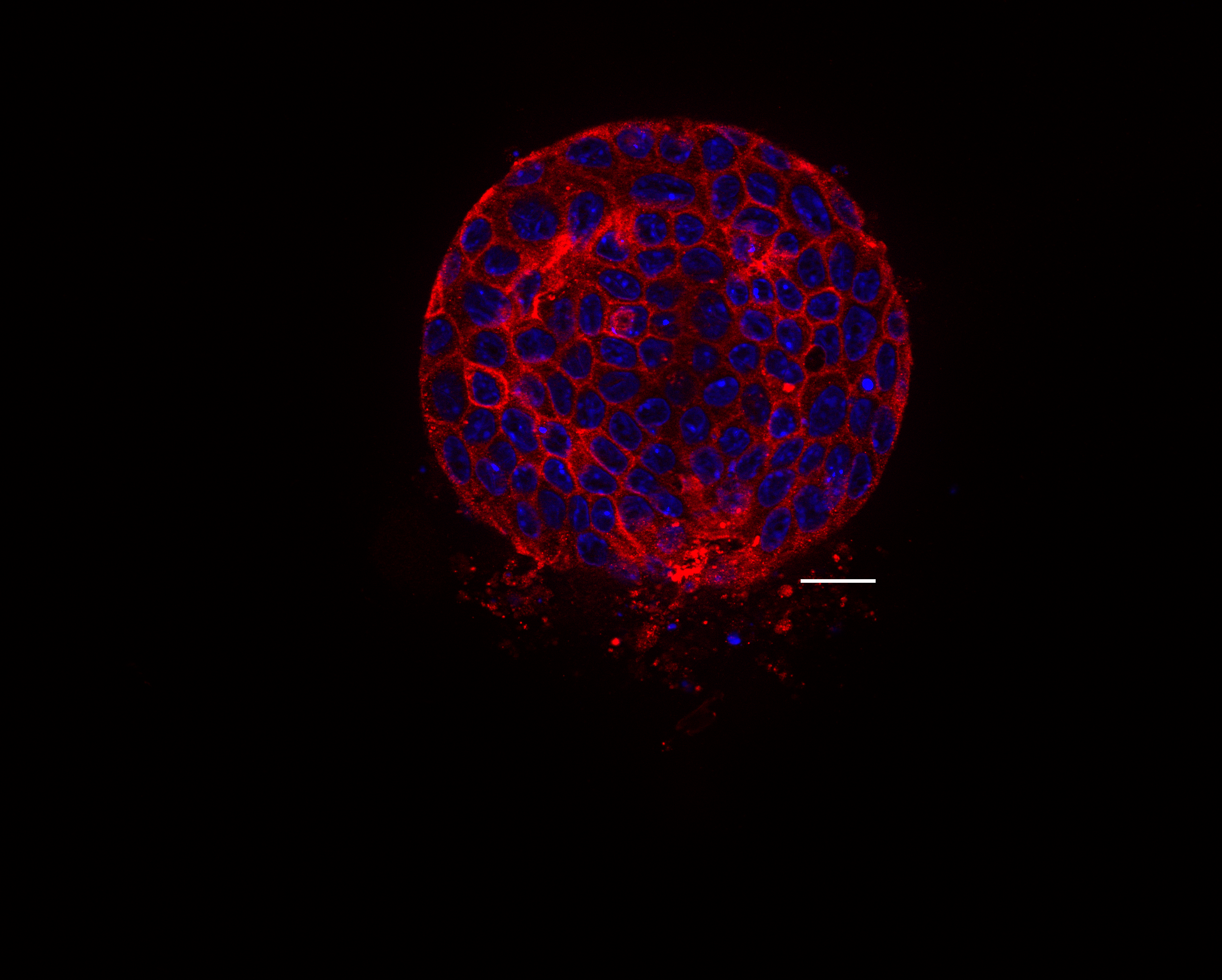

Supplement: Supplementary file 6 — Source data Fig. 4 [file 44321_2024_66_MOESM6_ESM.zip › Figure 4/4E/40X ZO1 RED WT COLON.tif_files/40X ZO1 RED MUC2 GREEN WT COLON-Bar.tif]

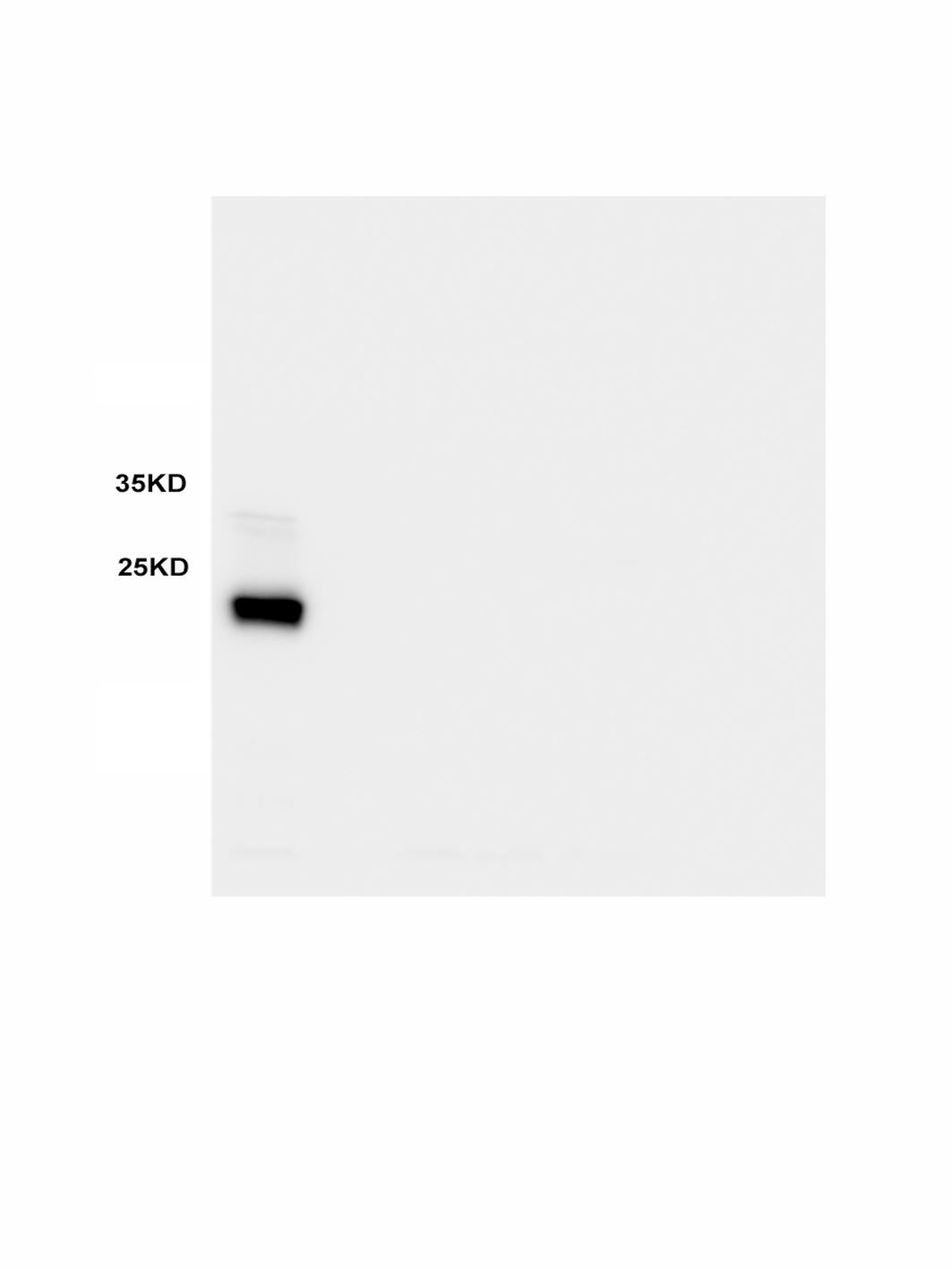

Supplement: Supplementary file 7 — Source data Fig. 5 [file 44321_2024_66_MOESM7_ESM.zip › Figure 5/5A/CBX3 HT29 KO WT (Chemiluminescence).tif]

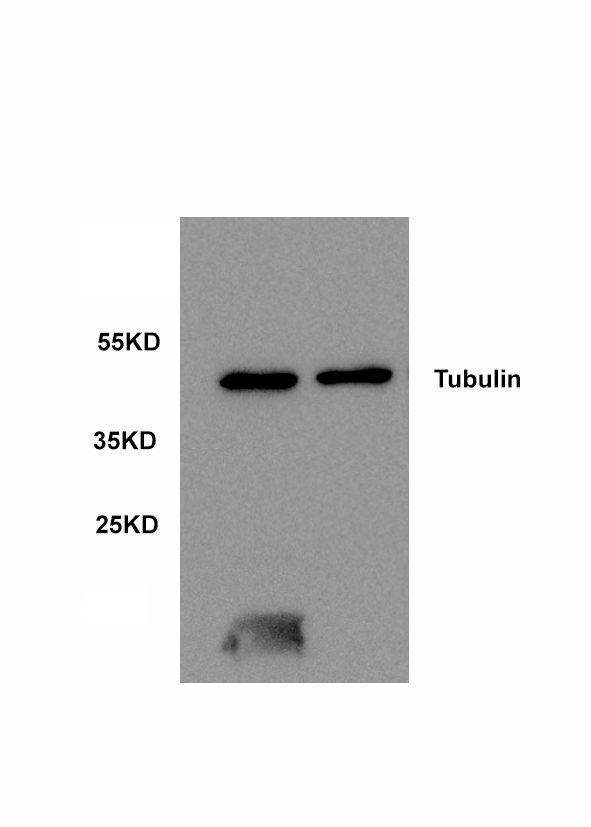

Supplement: Supplementary file 7 — Source data Fig. 5 [file 44321_2024_66_MOESM7_ESM.zip › Figure 5/5A/Tubulin gamma HT29 KO WT(Chemiluminescence).tif]

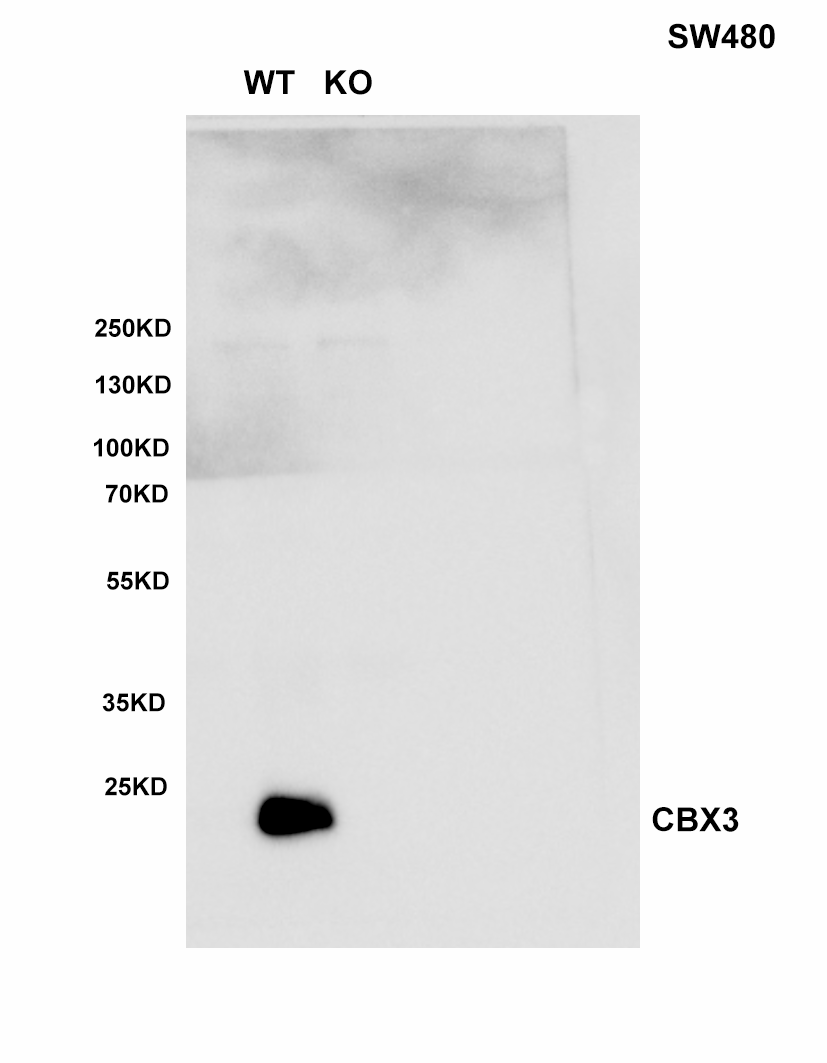

Supplement: Supplementary file 7 — Source data Fig. 5 [file 44321_2024_66_MOESM7_ESM.zip › Figure 5/5B/CBX3SW480 WT KO(Chemiluminescence).tif]

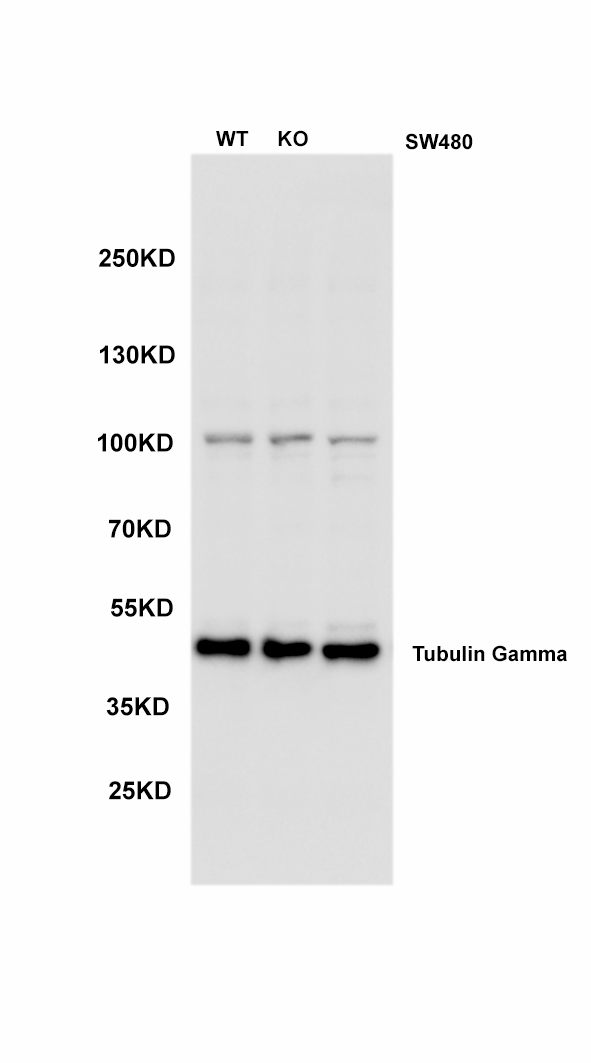

Supplement: Supplementary file 7 — Source data Fig. 5 [file 44321_2024_66_MOESM7_ESM.zip › Figure 5/5B/Tubulin gamma SW480 WT KO(Chemiluminescence).tif]

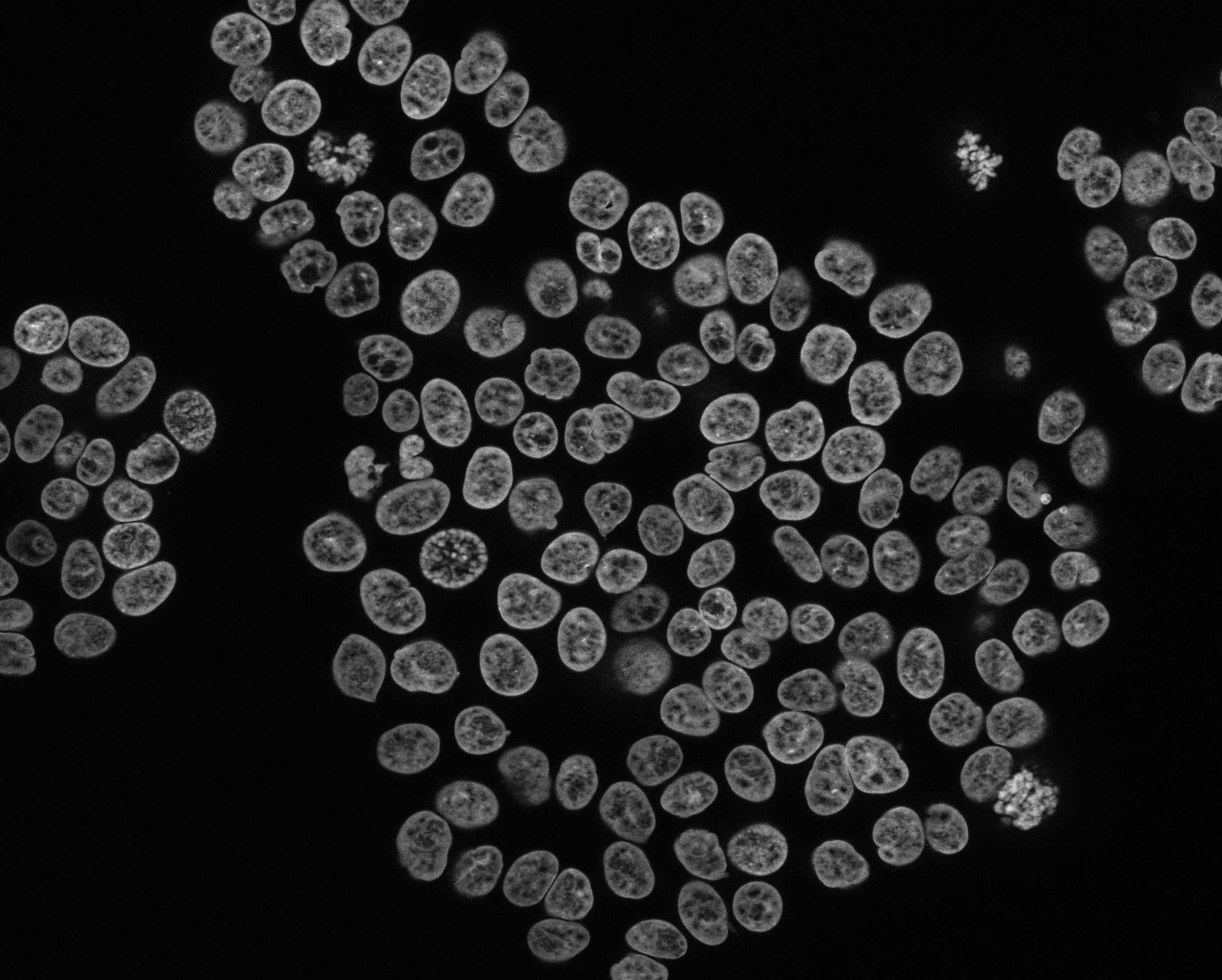

Supplement: Supplementary file 7 — Source data Fig. 5 [file 44321_2024_66_MOESM7_ESM.zip › Figure 5/5C/IF/HT29 KO CTRL STAT1 G CBX3 R1 .tif_files/HT29 06 CTRL STAT1 G CBX3 R1 _h0b0c0x0-2752y0-2208.tif]

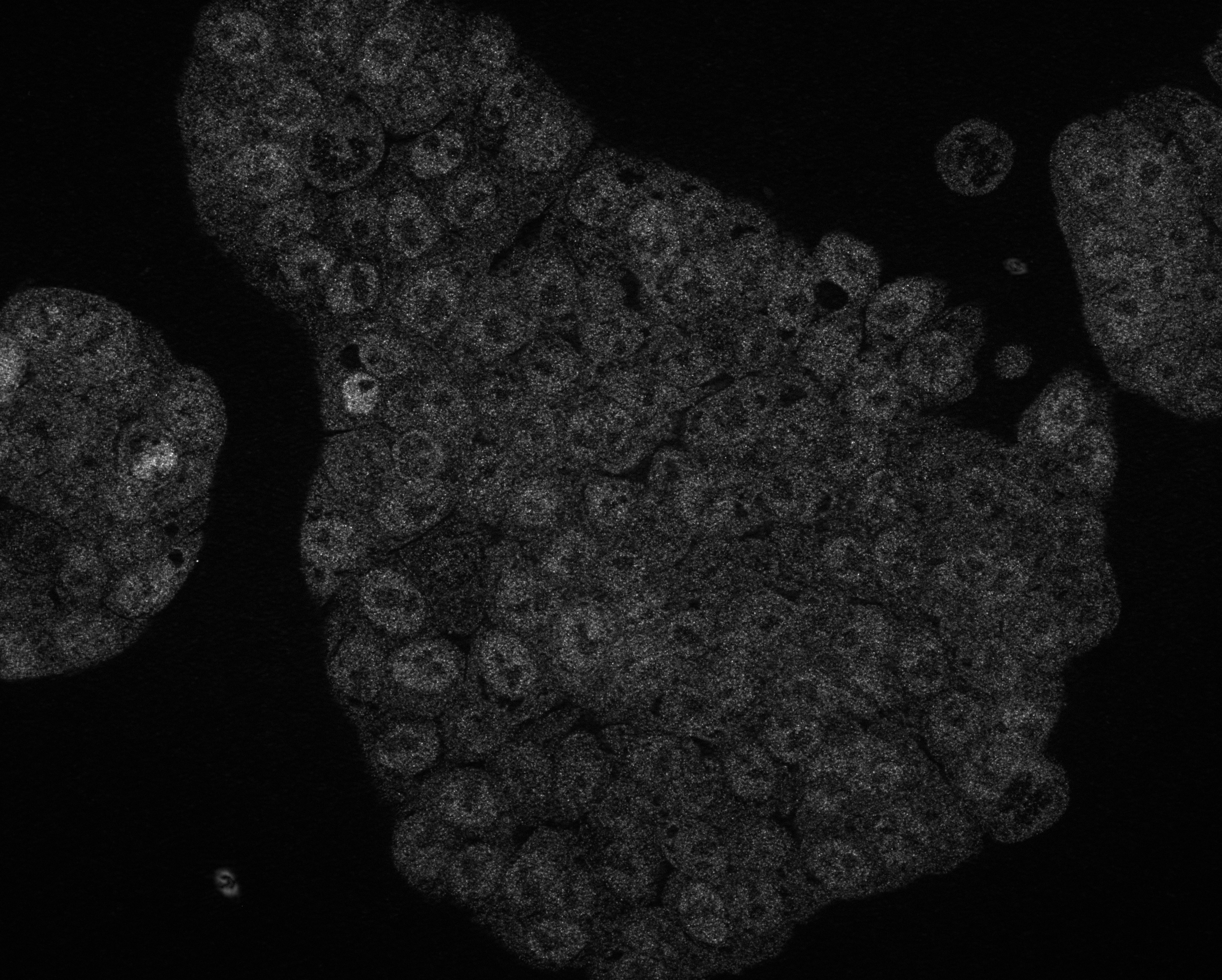

Supplement: Supplementary file 7 — Source data Fig. 5 [file 44321_2024_66_MOESM7_ESM.zip › Figure 5/5C/IF/HT29 KO CTRL STAT1 G CBX3 R1 .tif_files/HT29 06 CTRL STAT1 G CBX3 R1 _h0b0c1x0-2752y0-2208.tif]

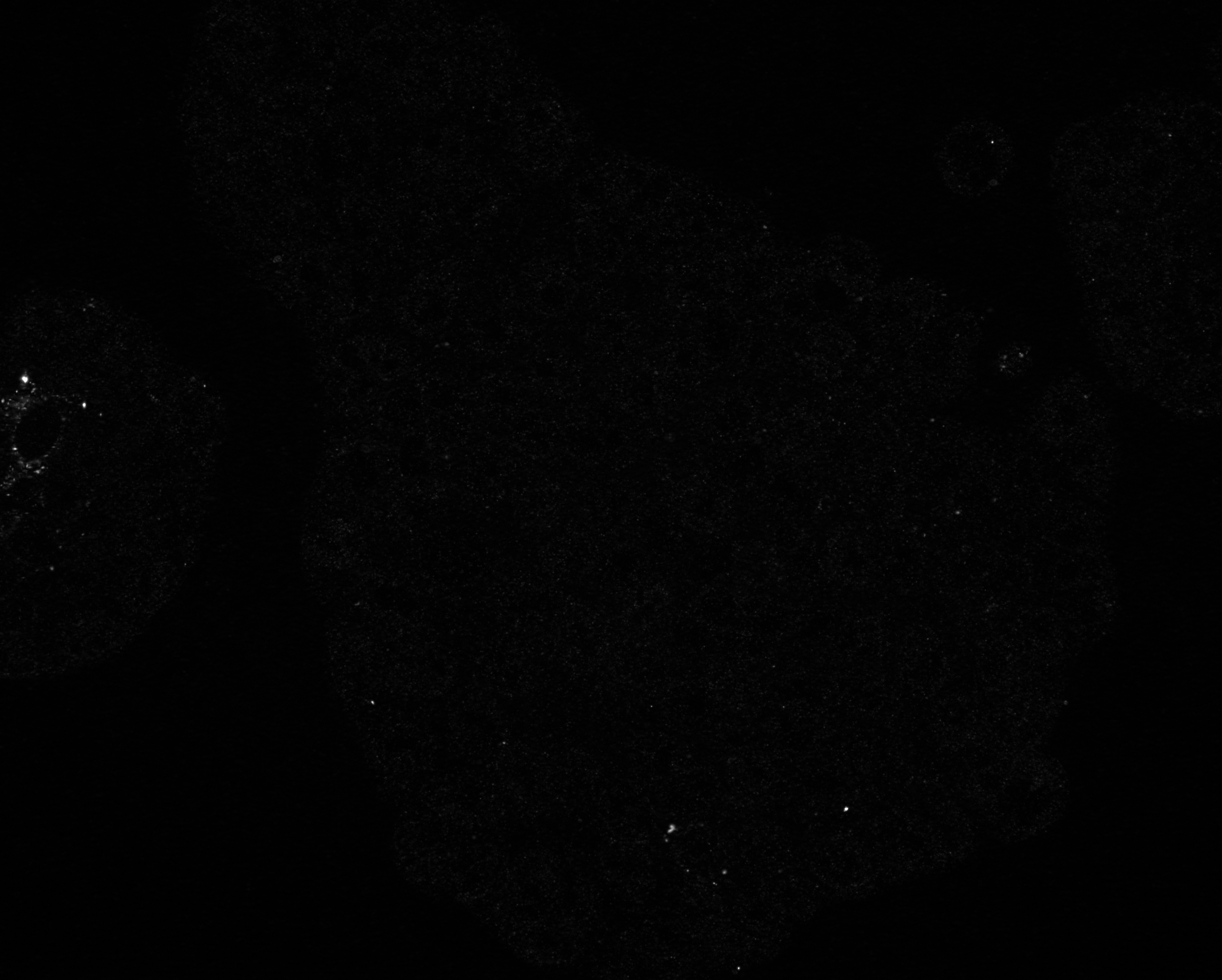

Supplement: Supplementary file 7 — Source data Fig. 5 [file 44321_2024_66_MOESM7_ESM.zip › Figure 5/5C/IF/HT29 KO CTRL STAT1 G CBX3 R1 .tif_files/HT29 06 CTRL STAT1 G CBX3 R1 _h0b0c2x0-2752y0-2208.tif]

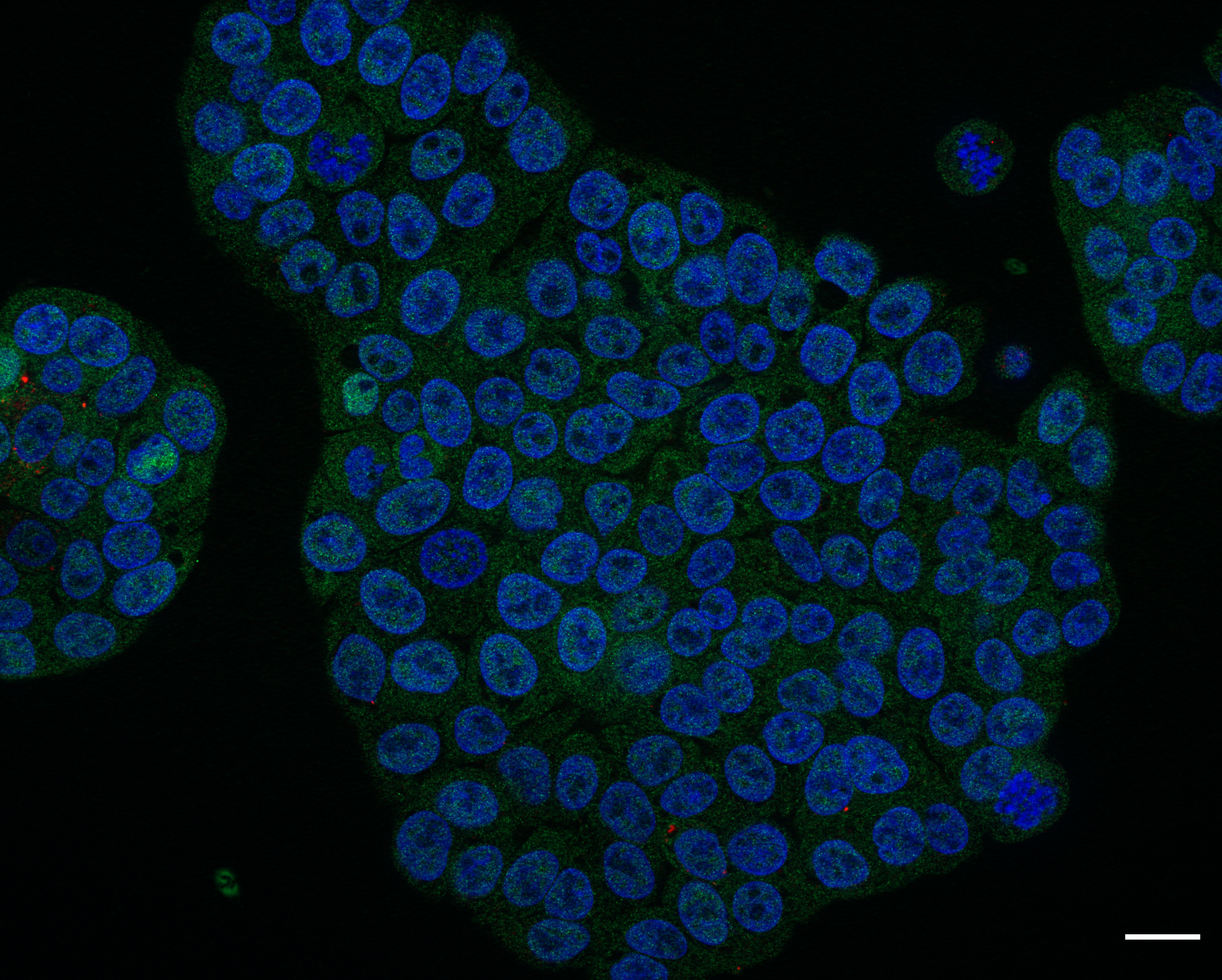

Supplement: Supplementary file 7 — Source data Fig. 5 [file 44321_2024_66_MOESM7_ESM.zip › Figure 5/5C/IF/HT29 KO CTRL STAT1 G CBX3 R1 .tif_files/HT29 CBX3KO CTRL STAT1 G CBX3 R.tif]

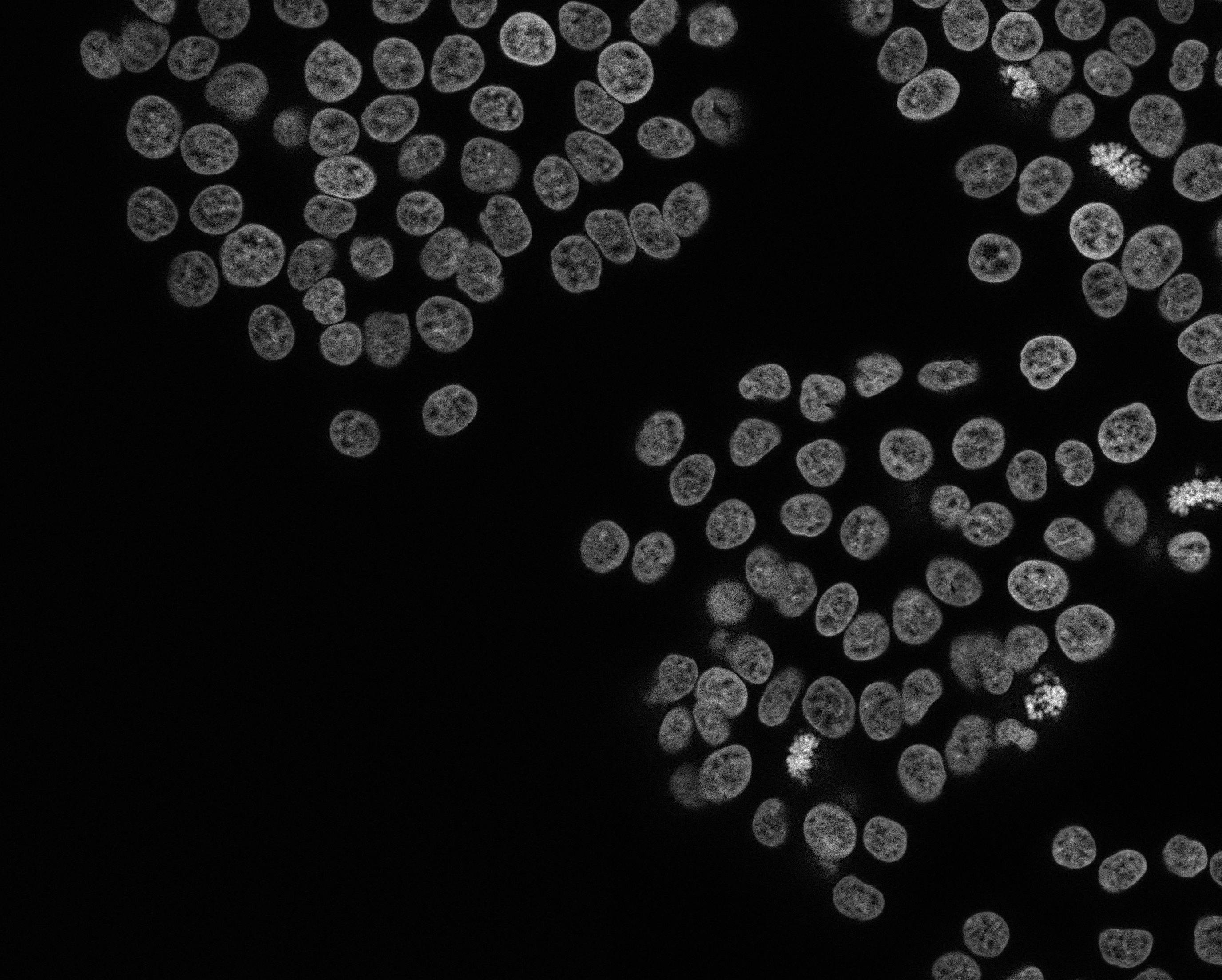

Supplement: Supplementary file 7 — Source data Fig. 5 [file 44321_2024_66_MOESM7_ESM.zip › Figure 5/5C/IF/HT29 KO IFN STAT1 G CBX3 R 1.tif_files/HT29 06 IFN STAT1 G CBX3 R 1_h0b0c0x0-2752y0-2208.tif]

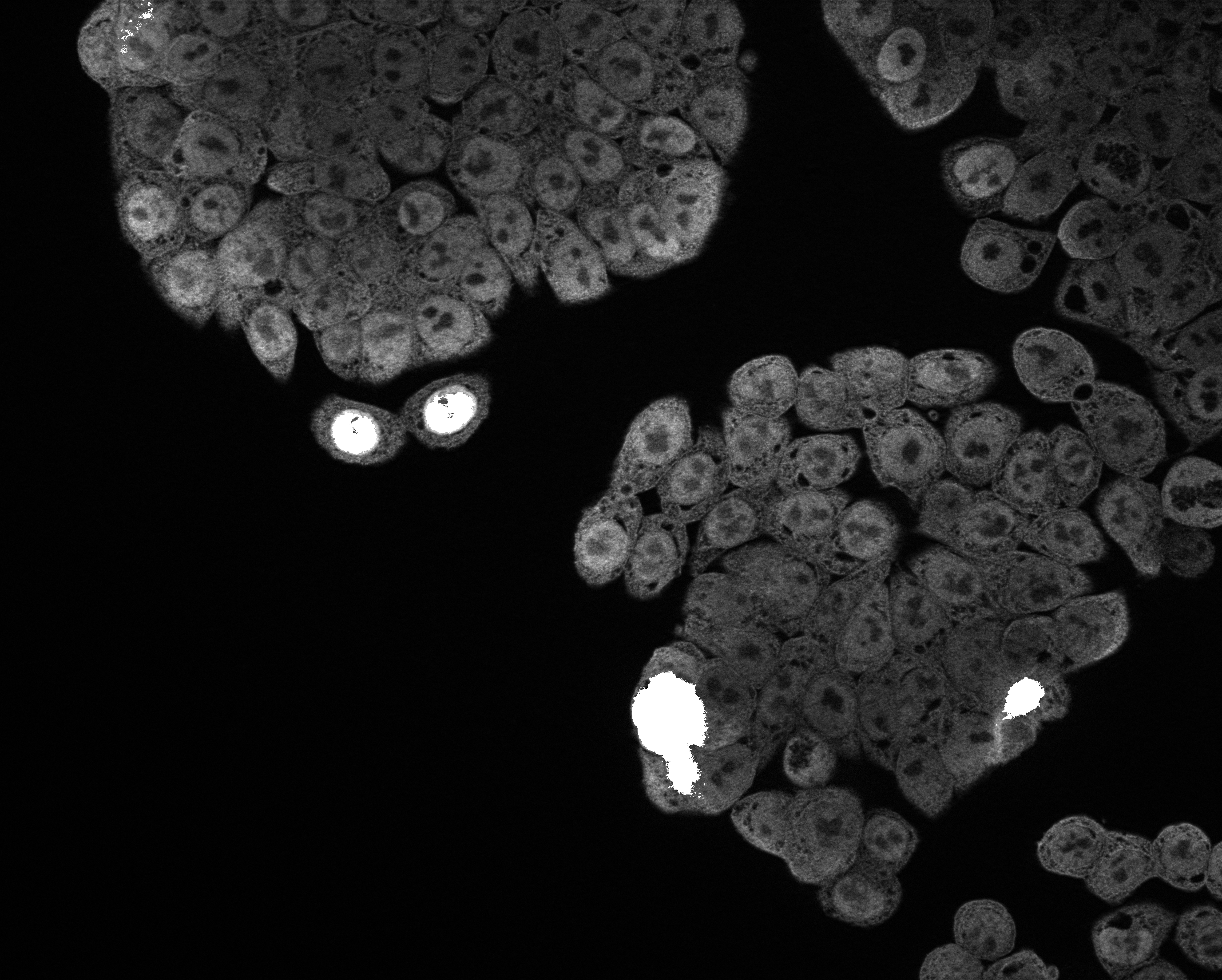

Supplement: Supplementary file 7 — Source data Fig. 5 [file 44321_2024_66_MOESM7_ESM.zip › Figure 5/5C/IF/HT29 KO IFN STAT1 G CBX3 R 1.tif_files/HT29 06 IFN STAT1 G CBX3 R 1_h0b0c1x0-2752y0-2208.tif]

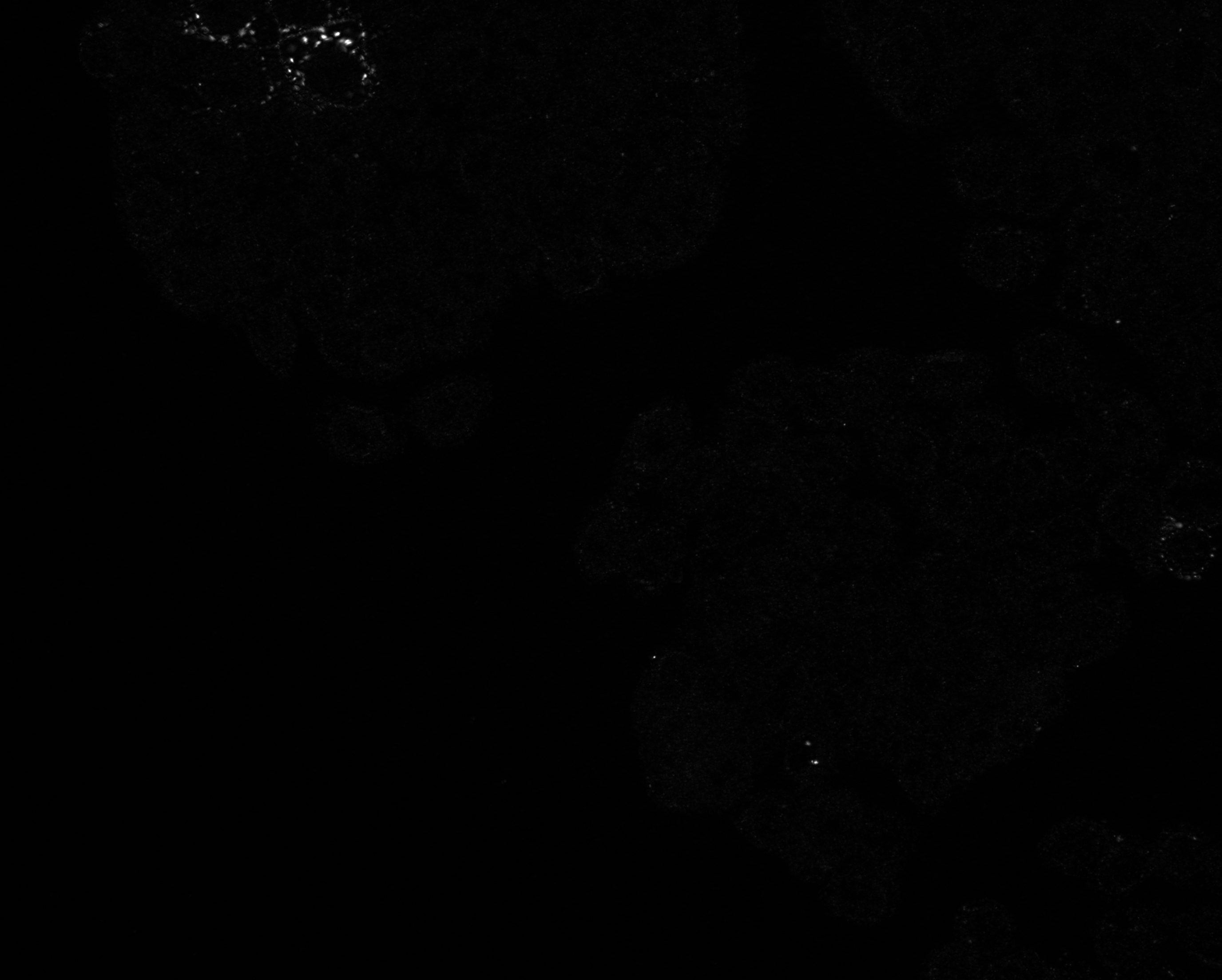

Supplement: Supplementary file 7 — Source data Fig. 5 [file 44321_2024_66_MOESM7_ESM.zip › Figure 5/5C/IF/HT29 KO IFN STAT1 G CBX3 R 1.tif_files/HT29 06 IFN STAT1 G CBX3 R 1_h0b0c2x0-2752y0-2208.tif]

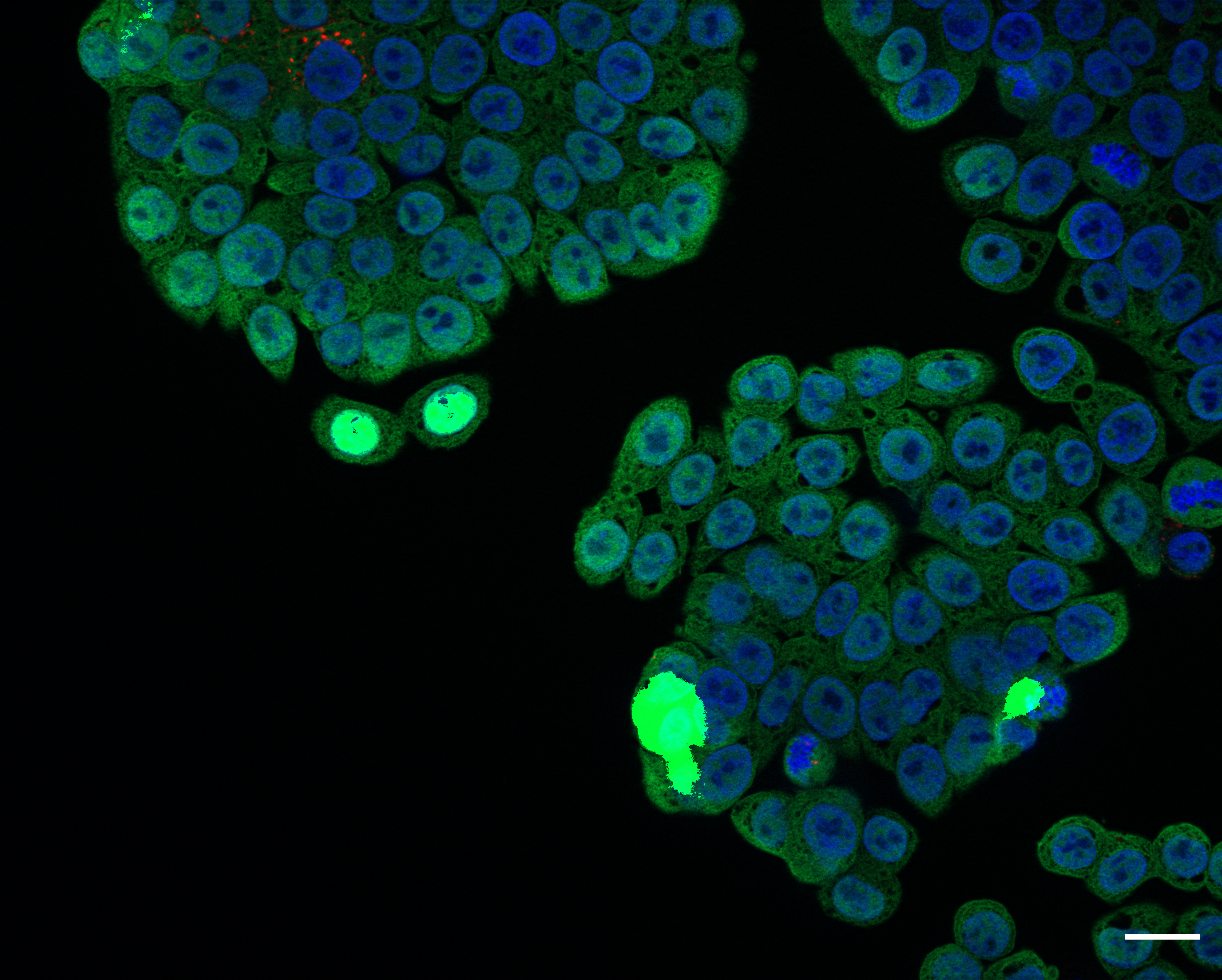

Supplement: Supplementary file 7 — Source data Fig. 5 [file 44321_2024_66_MOESM7_ESM.zip › Figure 5/5C/IF/HT29 KO IFN STAT1 G CBX3 R 1.tif_files/HT29 CBX3KO IFNg STAT1 G CBX3 R.tif]

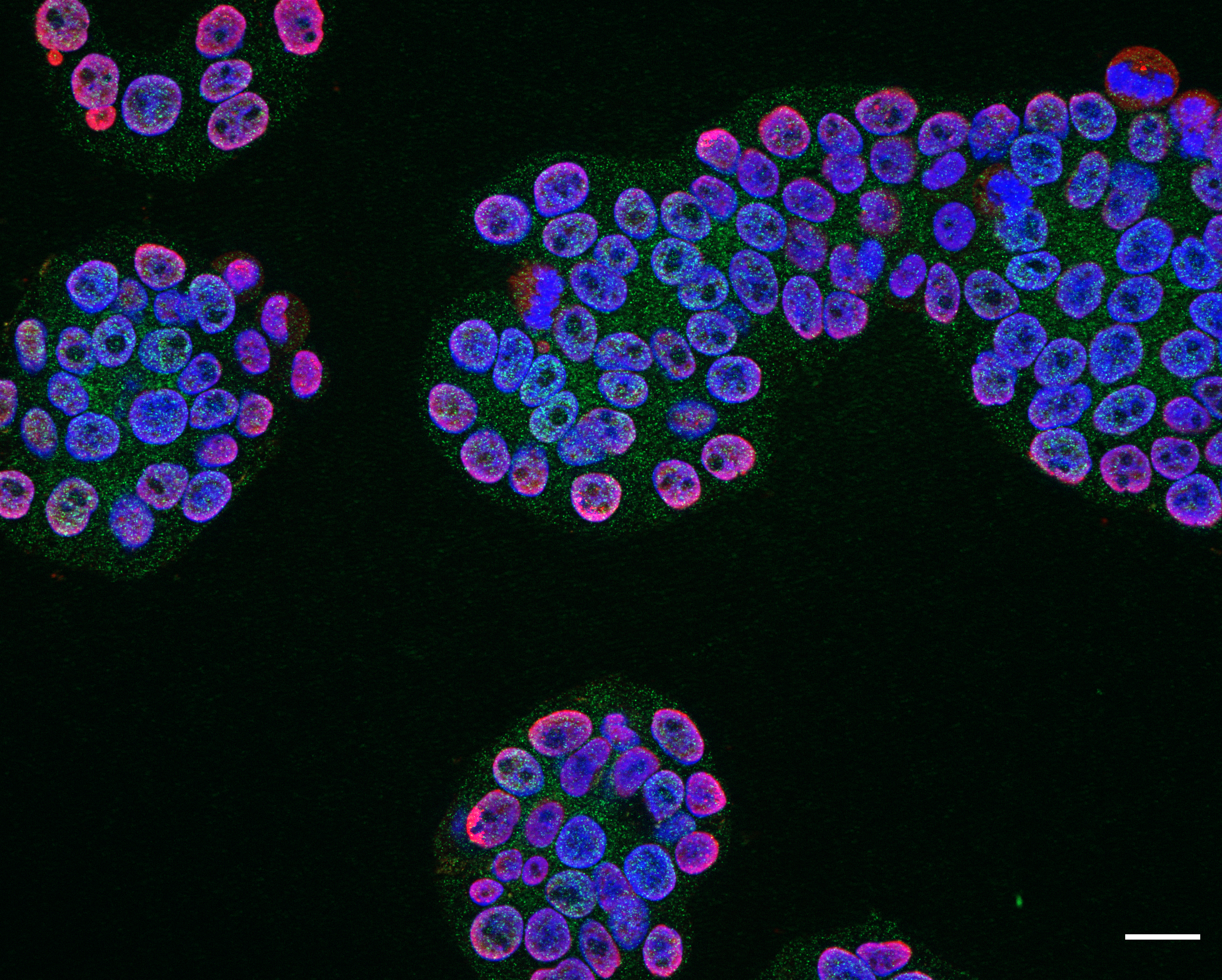

Supplement: Supplementary file 7 — Source data Fig. 5 [file 44321_2024_66_MOESM7_ESM.zip › Figure 5/5C/IF/HT29 WT CTRL STAT1 G cbx3 R1.tif_files/HT29 WT CTRL STAT1 G cbx3 R.tif]

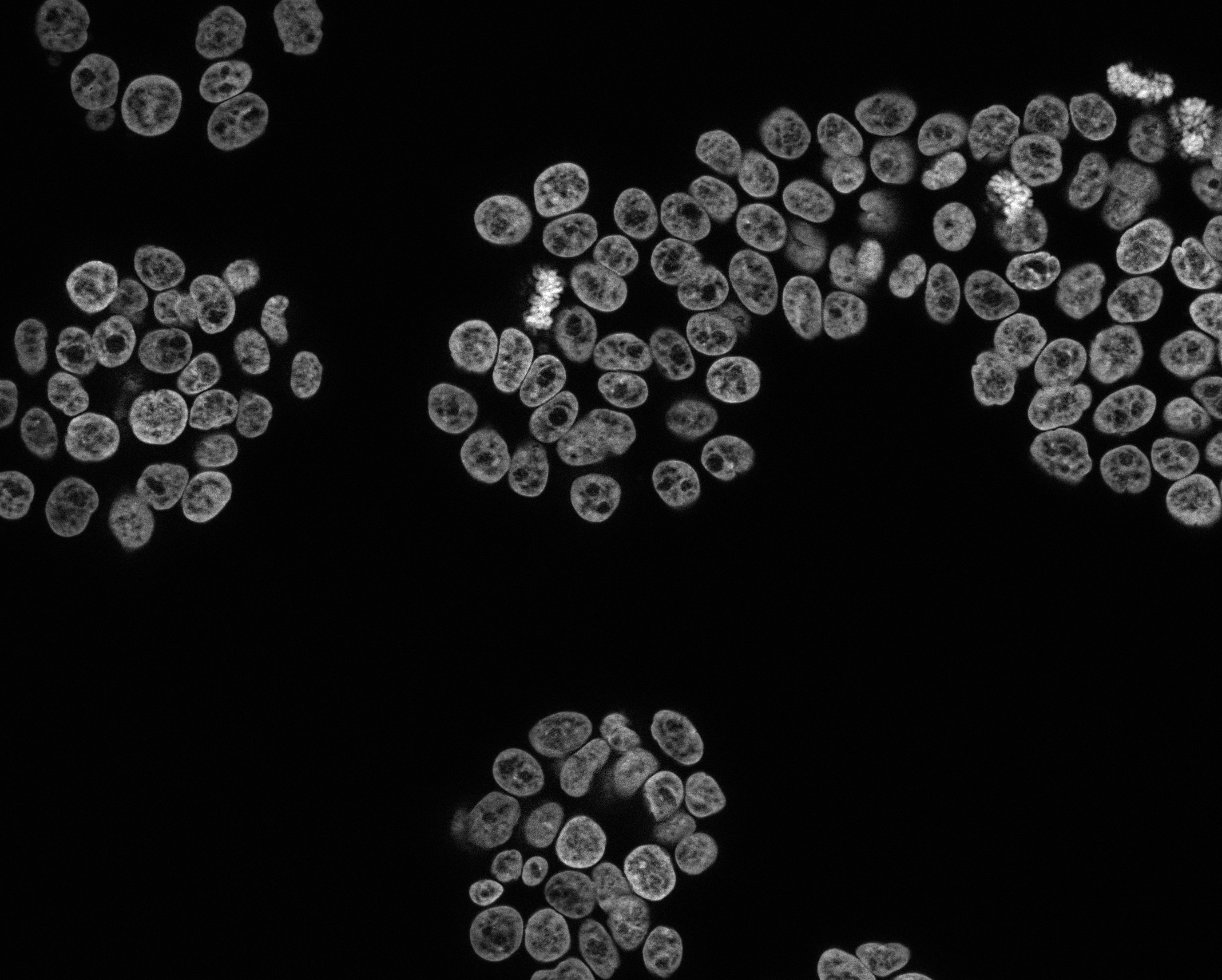

Supplement: Supplementary file 7 — Source data Fig. 5 [file 44321_2024_66_MOESM7_ESM.zip › Figure 5/5C/IF/HT29 WT CTRL STAT1 G cbx3 R1.tif_files/HT29 WT CTRL STAT1 G cbx3 R1_h0b0c0x0-2752y0-2208.tif]

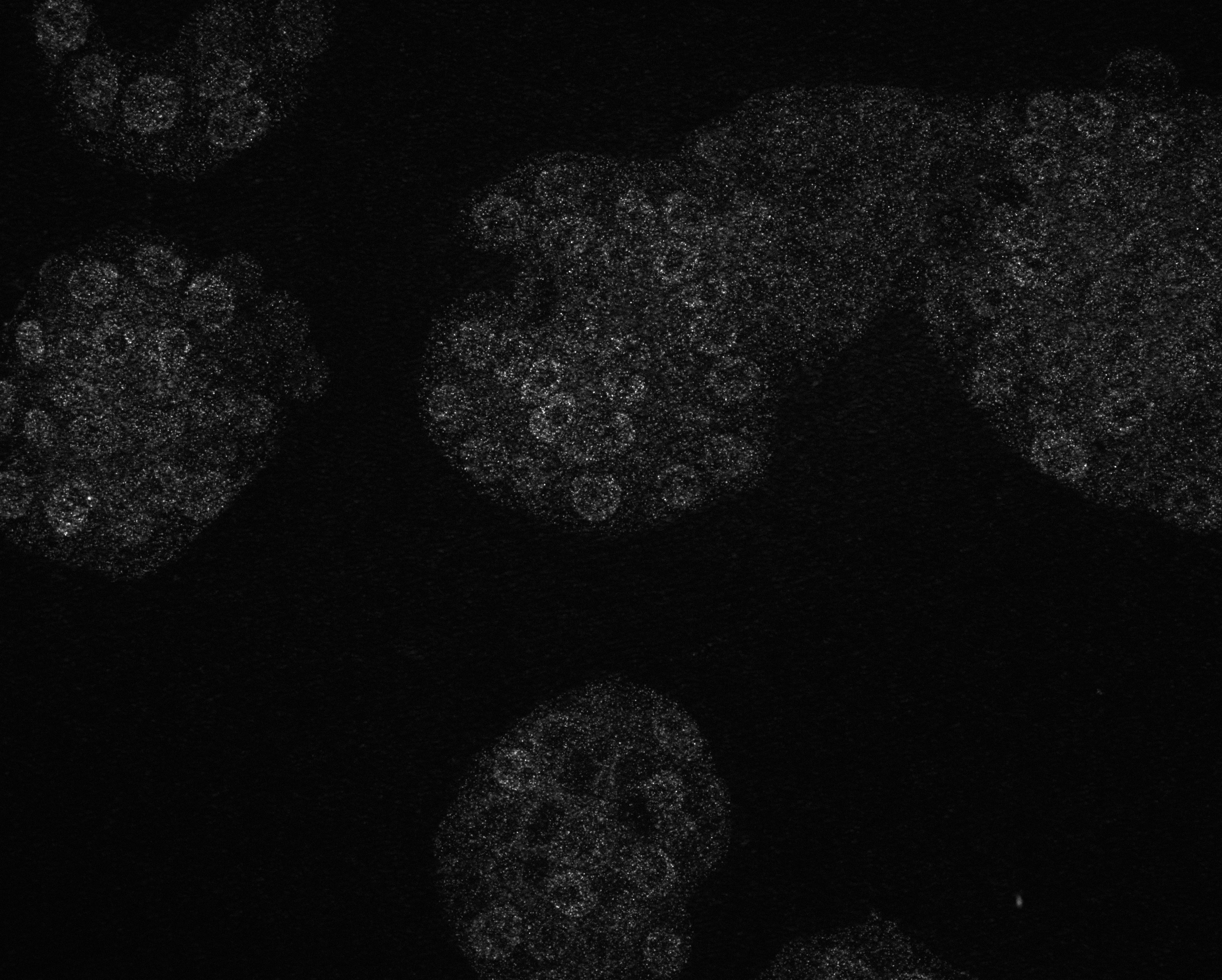

Supplement: Supplementary file 7 — Source data Fig. 5 [file 44321_2024_66_MOESM7_ESM.zip › Figure 5/5C/IF/HT29 WT CTRL STAT1 G cbx3 R1.tif_files/HT29 WT CTRL STAT1 G cbx3 R1_h0b0c1x0-2752y0-2208.tif]

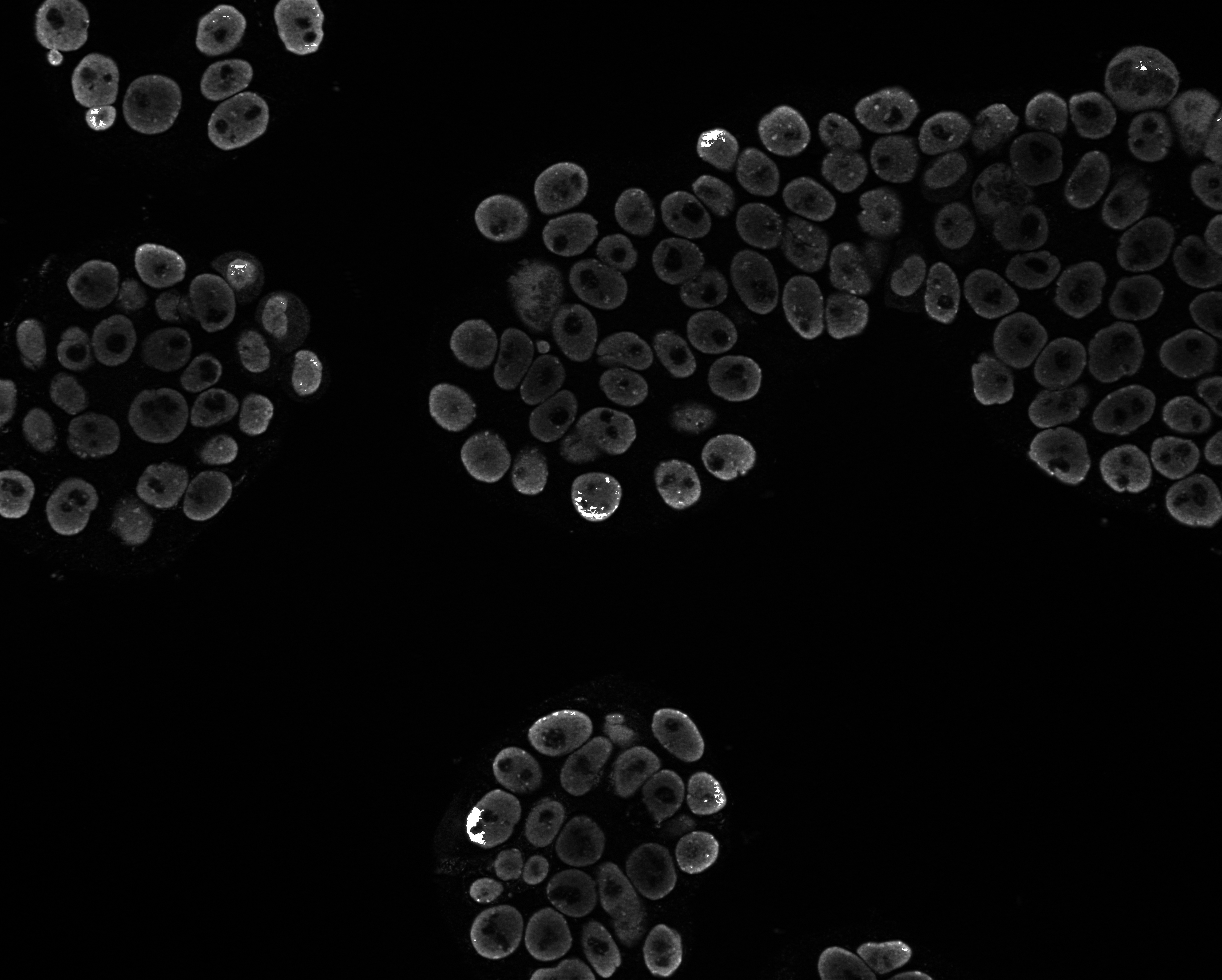

Supplement: Supplementary file 7 — Source data Fig. 5 [file 44321_2024_66_MOESM7_ESM.zip › Figure 5/5C/IF/HT29 WT CTRL STAT1 G cbx3 R1.tif_files/HT29 WT CTRL STAT1 G cbx3 R1_h0b0c2x0-2752y0-2208.tif]

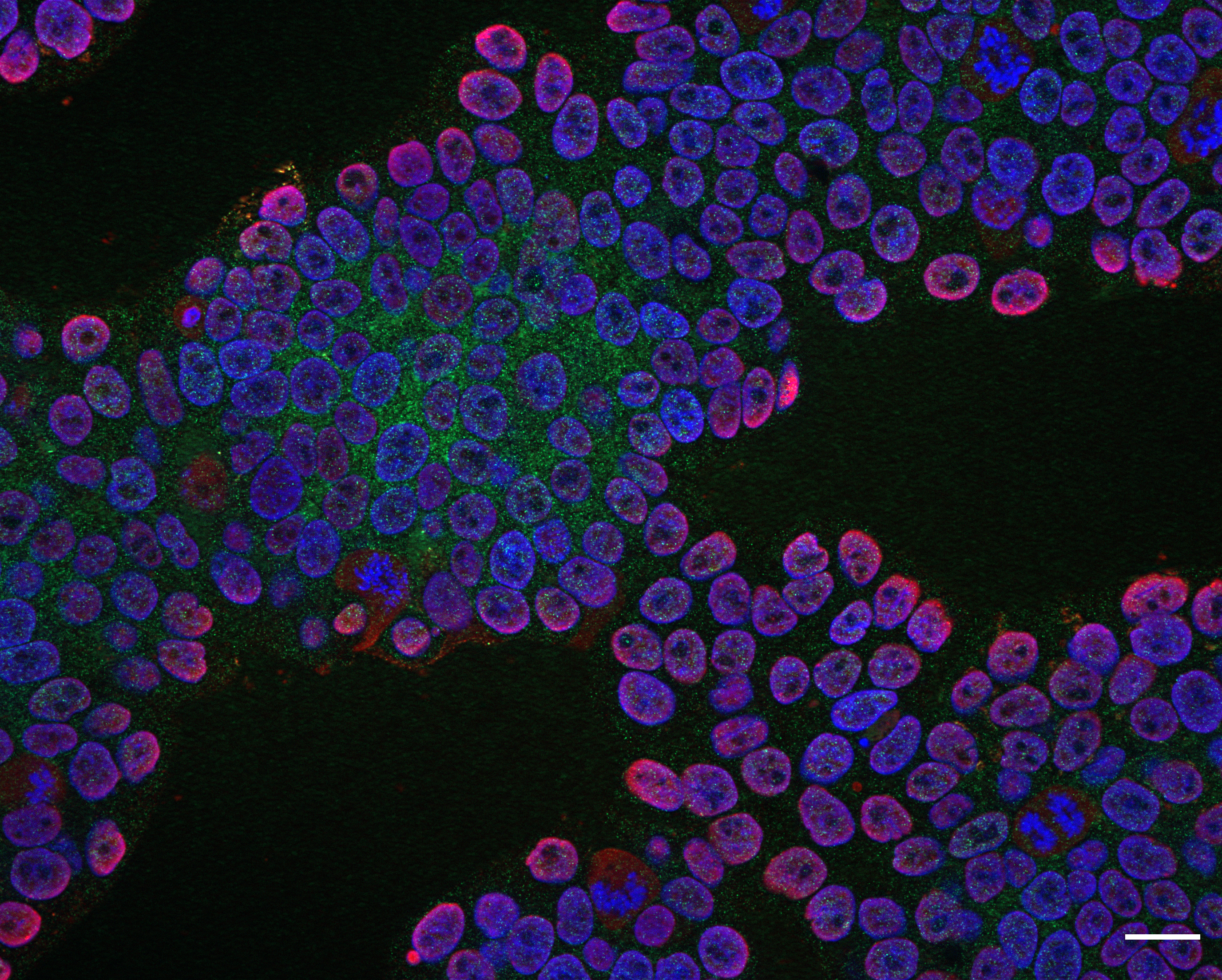

Supplement: Supplementary file 7 — Source data Fig. 5 [file 44321_2024_66_MOESM7_ESM.zip › Figure 5/5C/IF/HT29 WT IFN STAT1 G cbx3 R.tif_files/HT29 WT IFN STAT1 G cbx3 R.tif]

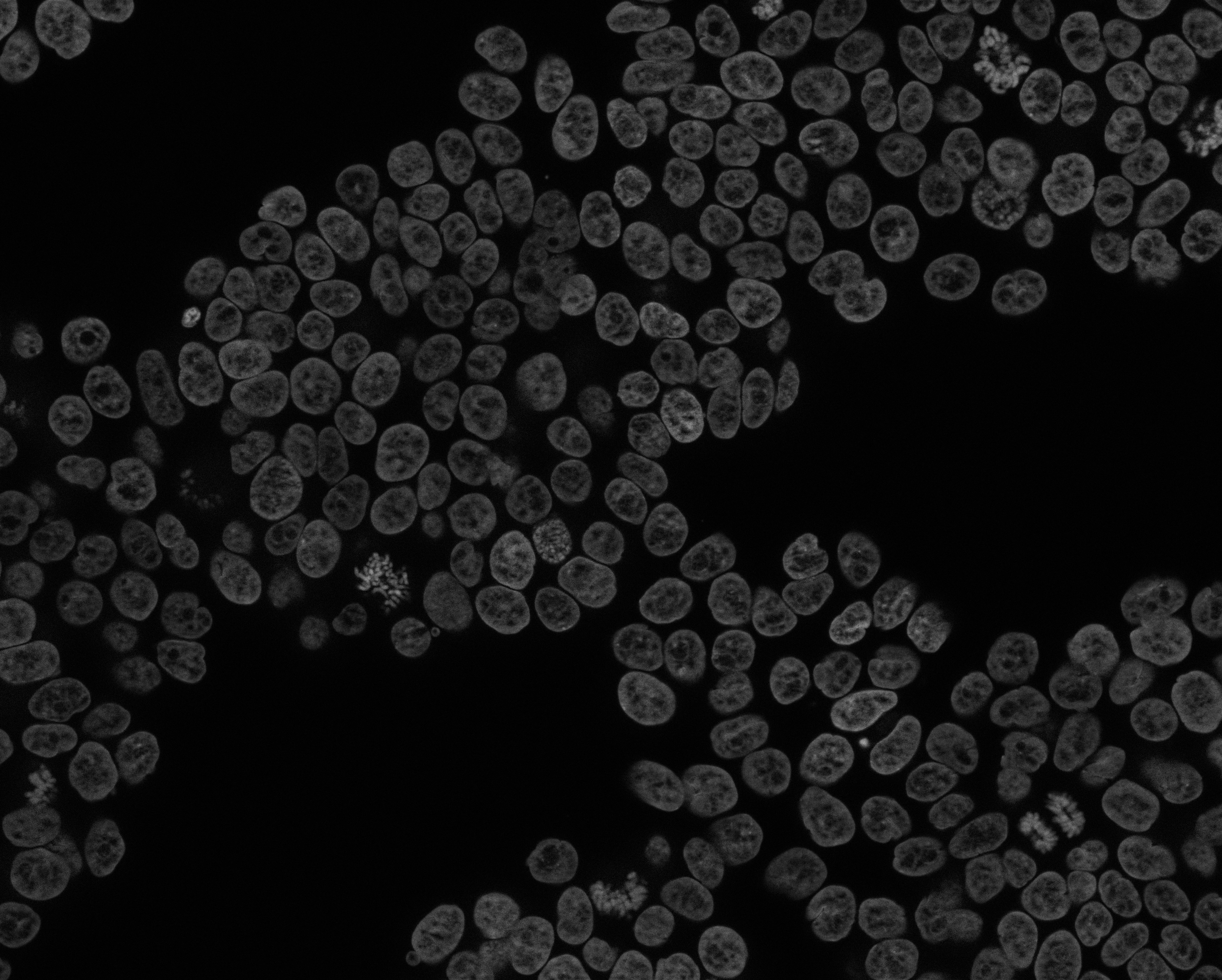

Supplement: Supplementary file 7 — Source data Fig. 5 [file 44321_2024_66_MOESM7_ESM.zip › Figure 5/5C/IF/HT29 WT IFN STAT1 G cbx3 R.tif_files/HT29 WT IFN STAT1 G cbx3 R_h0b0c0x0-2752y0-2208.tif]

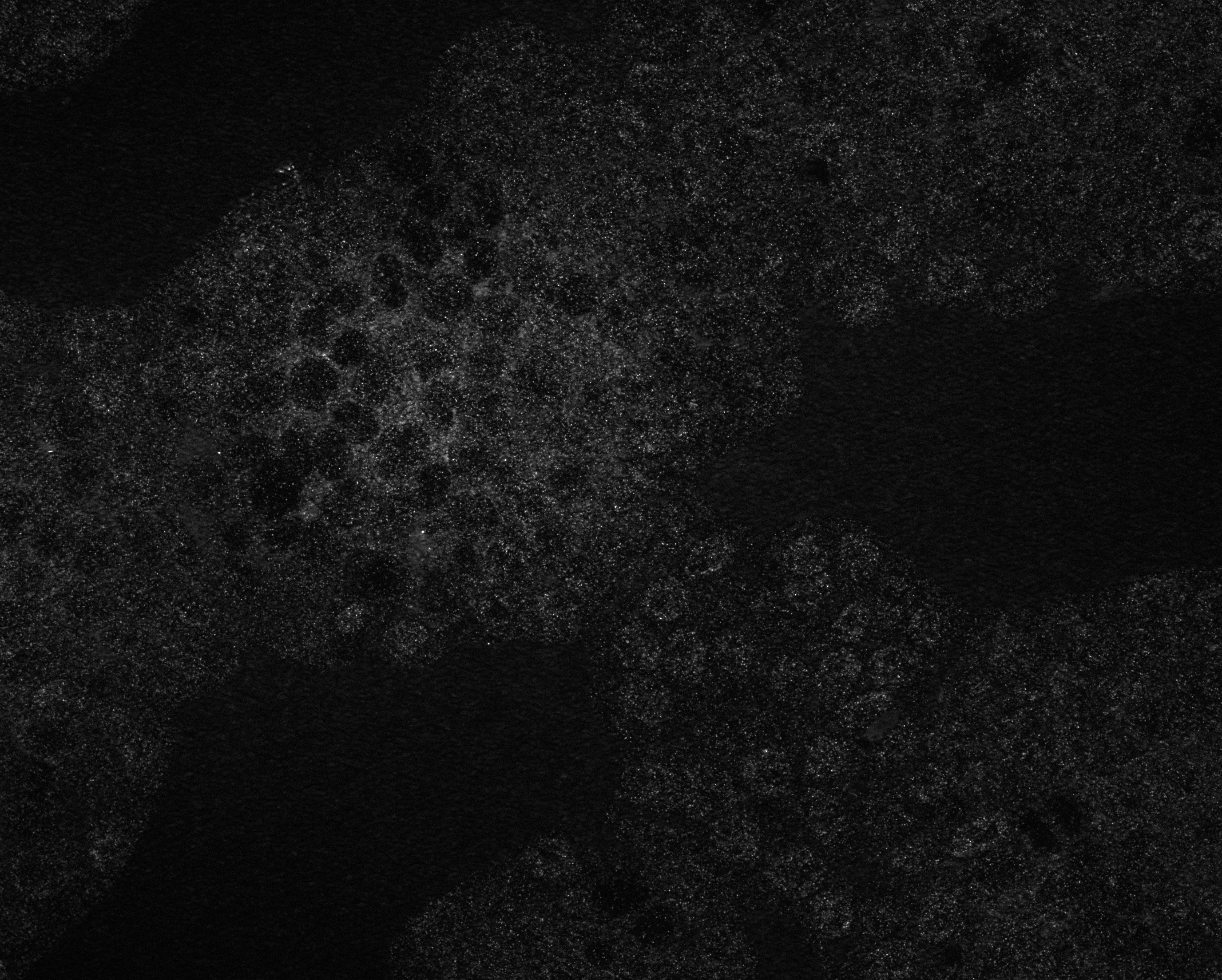

Supplement: Supplementary file 7 — Source data Fig. 5 [file 44321_2024_66_MOESM7_ESM.zip › Figure 5/5C/IF/HT29 WT IFN STAT1 G cbx3 R.tif_files/HT29 WT IFN STAT1 G cbx3 R_h0b0c1x0-2752y0-2208.tif]

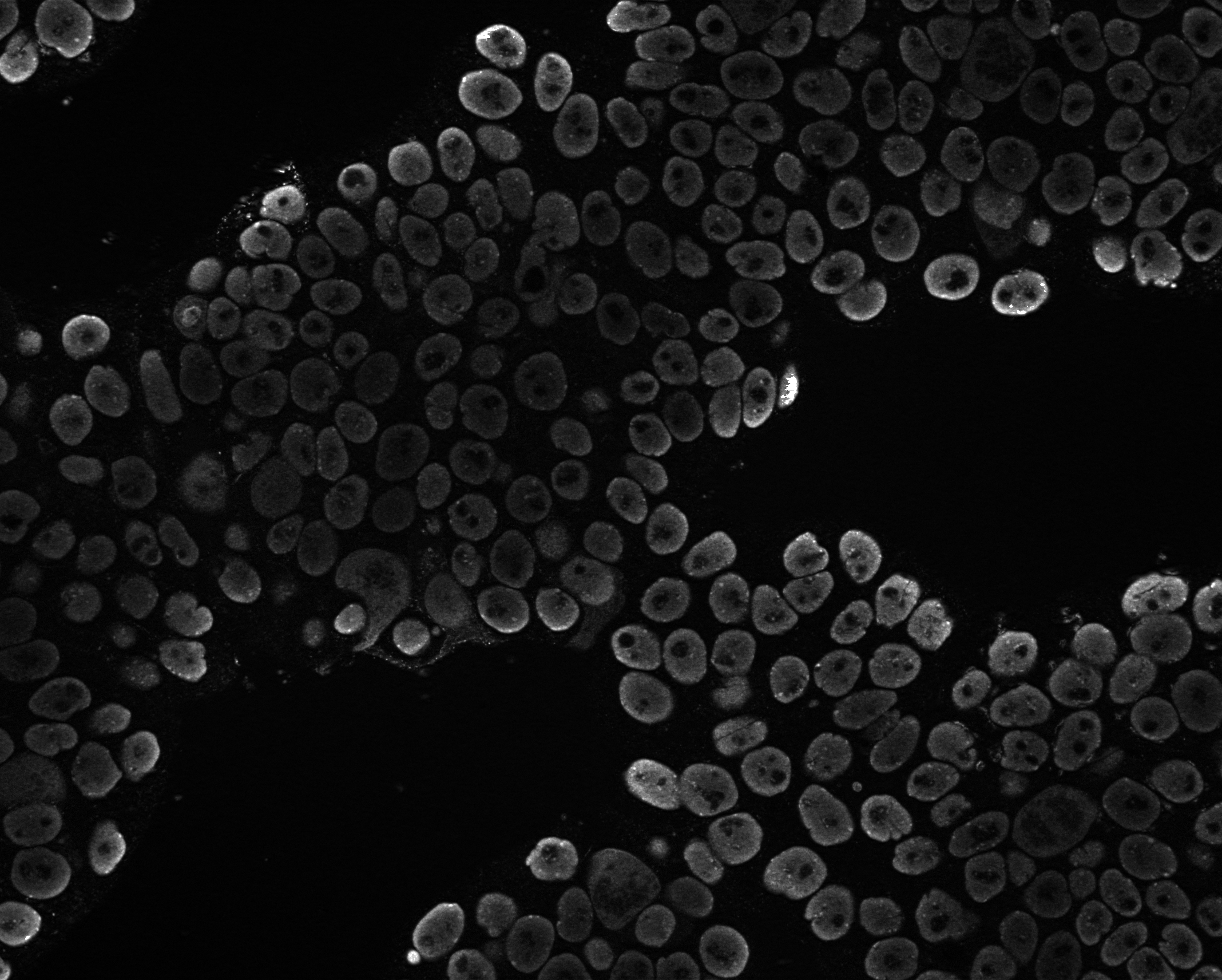

Supplement: Supplementary file 7 — Source data Fig. 5 [file 44321_2024_66_MOESM7_ESM.zip › Figure 5/5C/IF/HT29 WT IFN STAT1 G cbx3 R.tif_files/HT29 WT IFN STAT1 G cbx3 R_h0b0c2x0-2752y0-2208.tif]

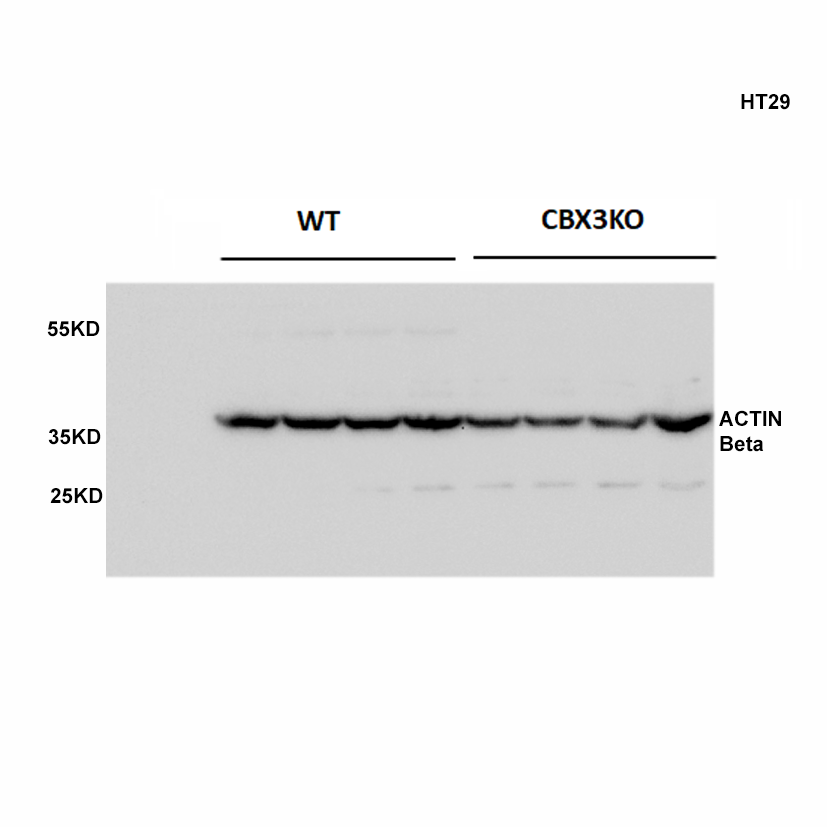

Supplement: Supplementary file 7 — Source data Fig. 5 [file 44321_2024_66_MOESM7_ESM.zip › Figure 5/5C/Western/beta actin ht29_6 WT KO (Chemiluminescence).tif]

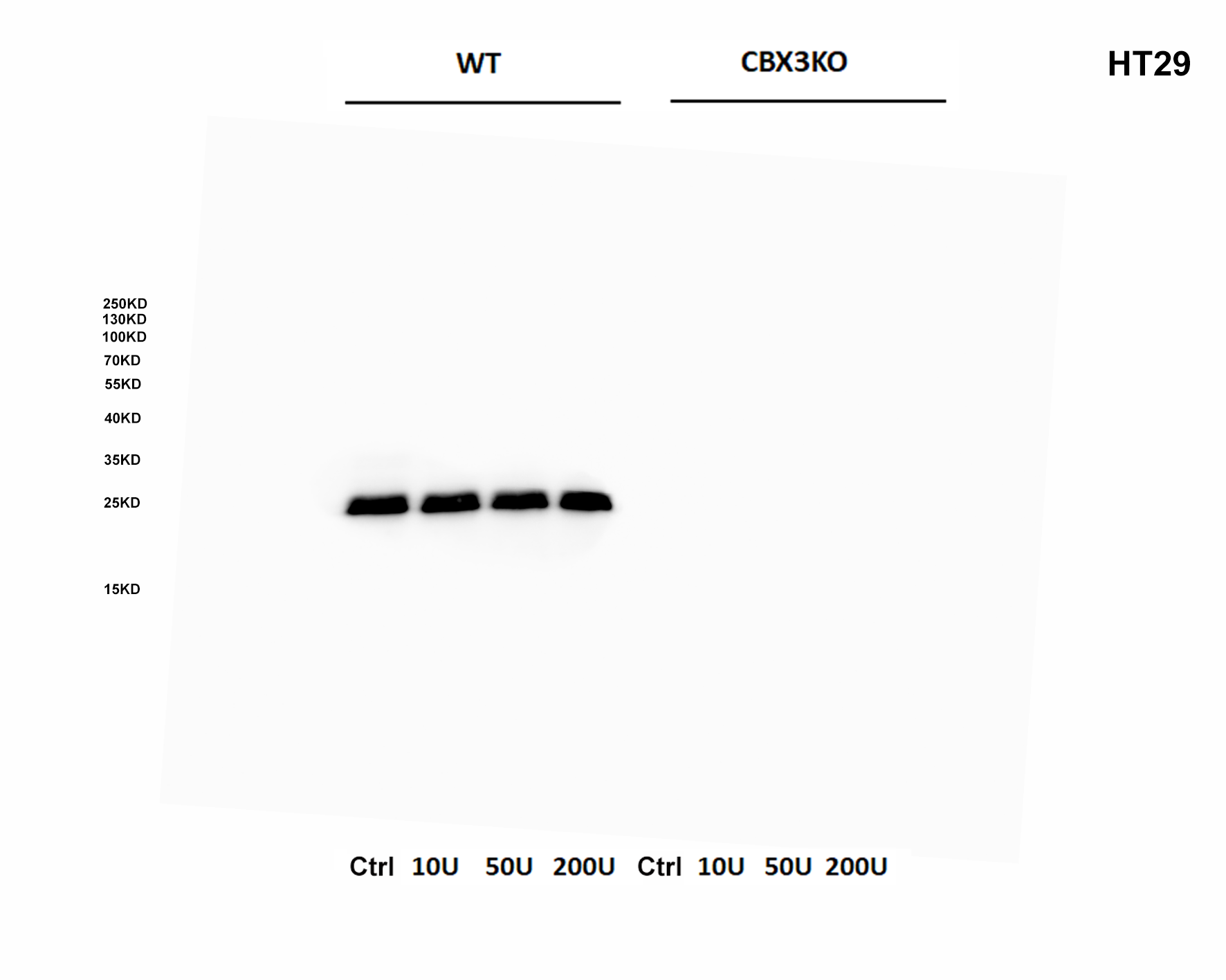

Supplement: Supplementary file 7 — Source data Fig. 5 [file 44321_2024_66_MOESM7_ESM.zip › Figure 5/5C/Western/hp1gamma ht29_1(Chemiluminescence).tif]

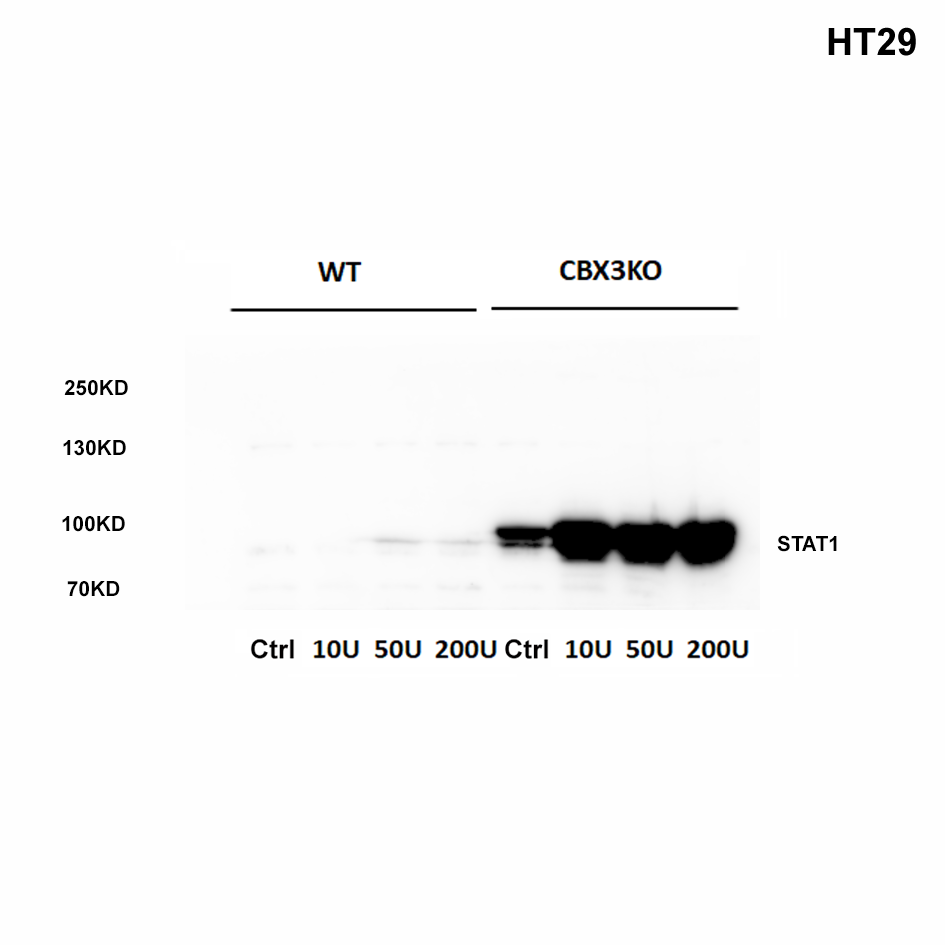

Supplement: Supplementary file 7 — Source data Fig. 5 [file 44321_2024_66_MOESM7_ESM.zip › Figure 5/5C/Western/HT29 STAT1 WTKO .tif]

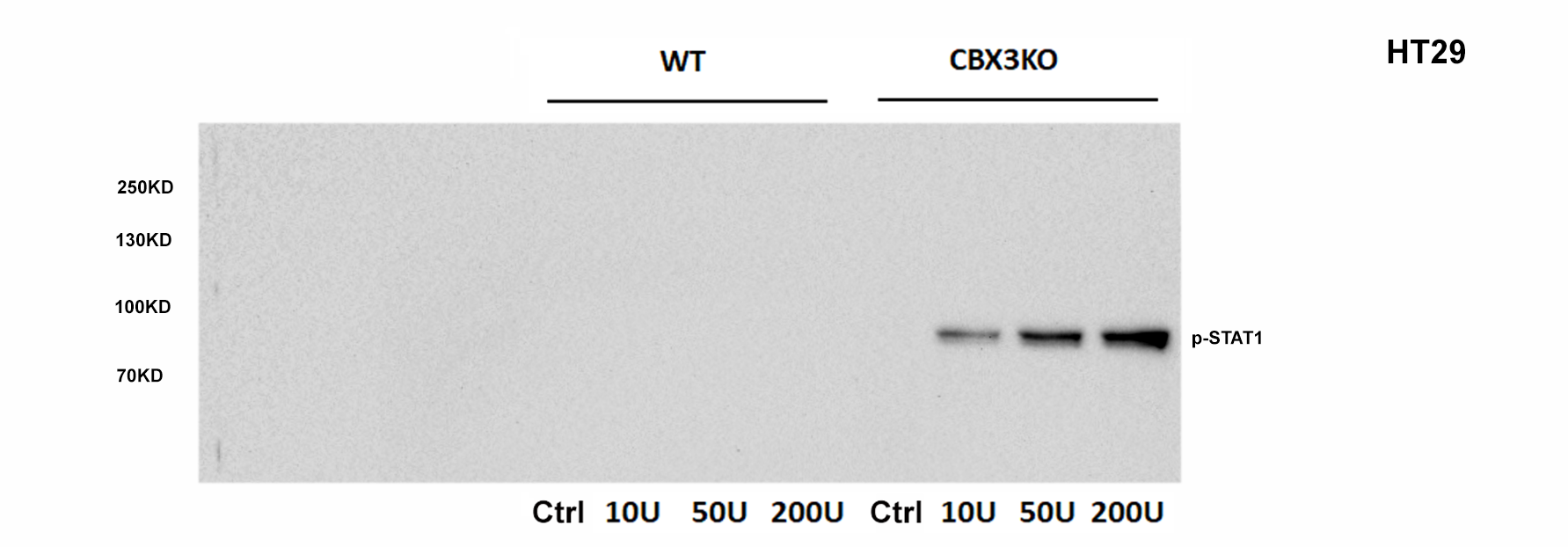

Supplement: Supplementary file 7 — Source data Fig. 5 [file 44321_2024_66_MOESM7_ESM.zip › Figure 5/5C/Western/pSTAT1 HT29 WT KO(Chemiluminescence).tif]

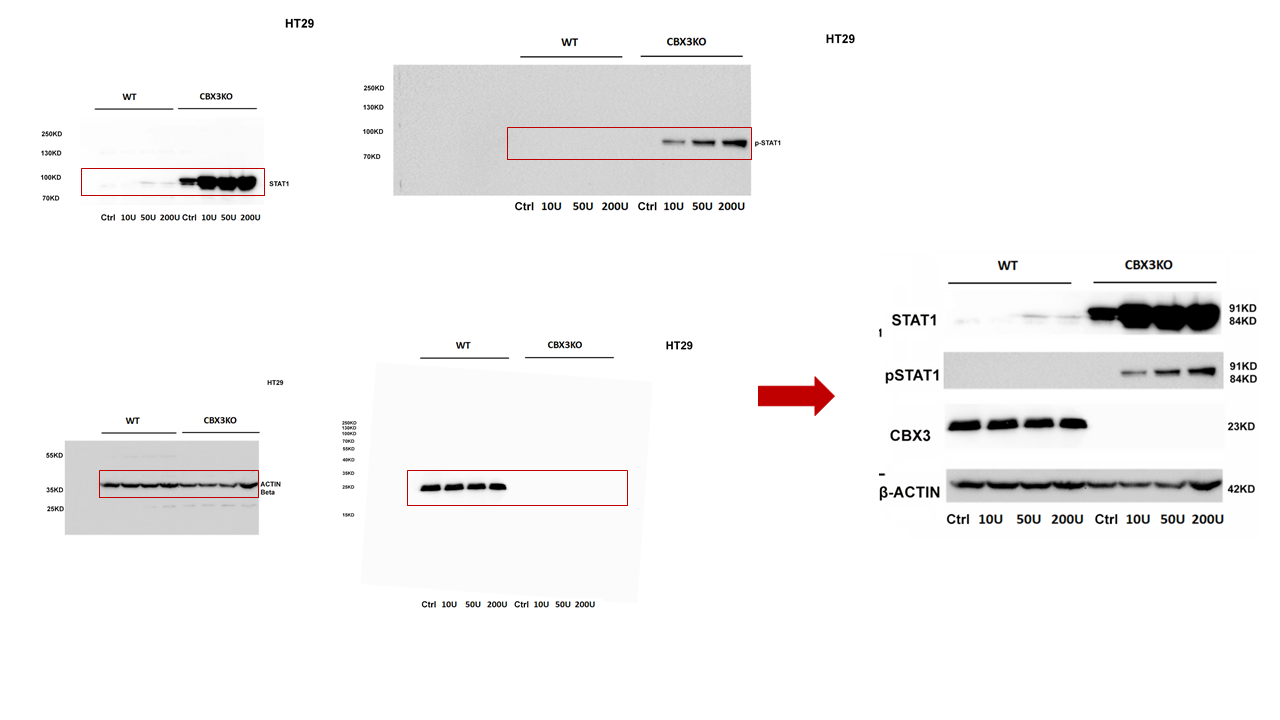

Supplement: Supplementary file 7 — Source data Fig. 5 [file 44321_2024_66_MOESM7_ESM.zip › Figure 5/5C/Western/resume HT29.tif]

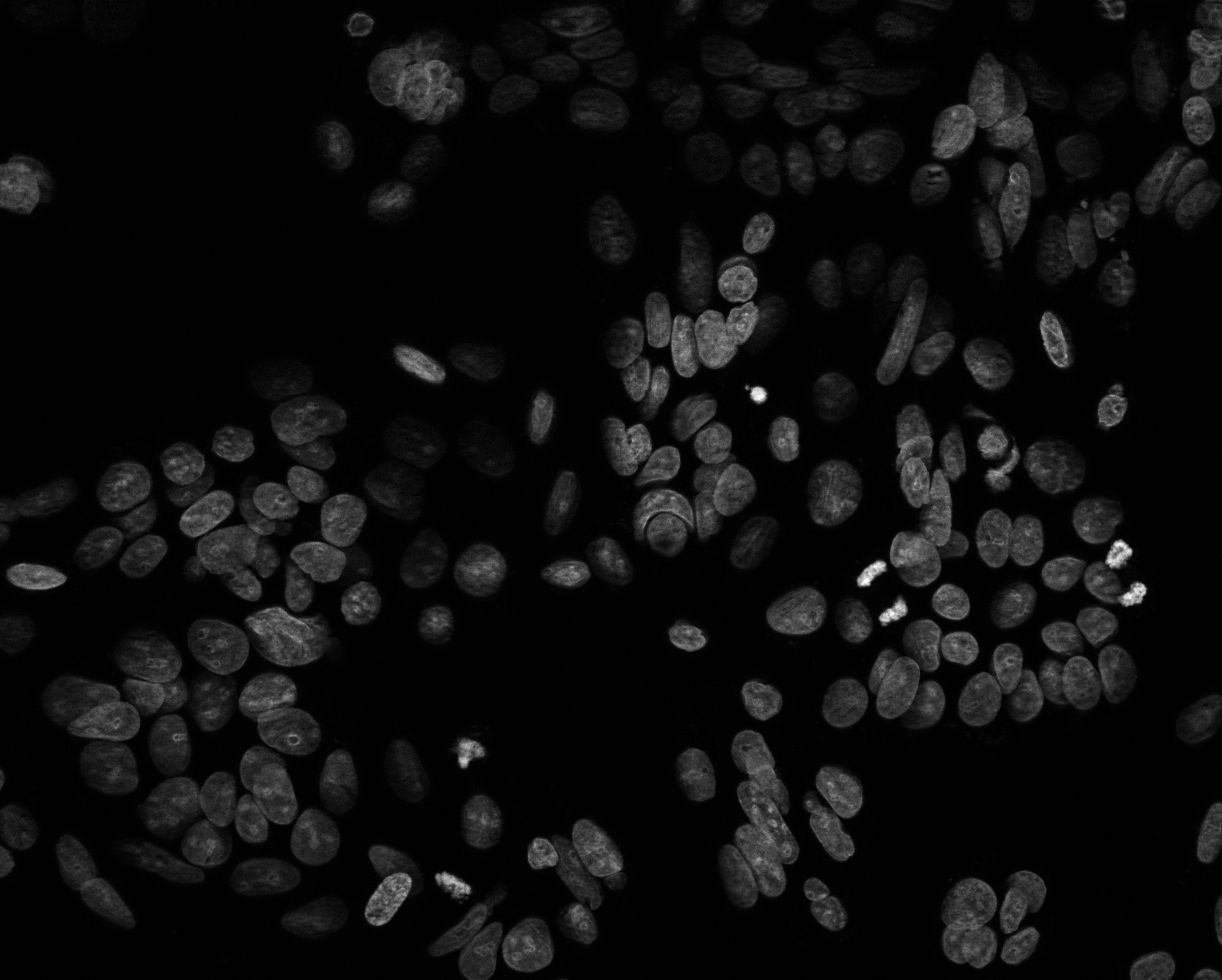

Supplement: Supplementary file 7 — Source data Fig. 5 [file 44321_2024_66_MOESM7_ESM.zip › Figure 5/5D/IF/KOSW480 IFNg STAT1Green CBX3Red 002.tif_files/C004sw480ifn stat1Vcbx3R 002_h0b0c0x0-2752y0-2208.tif]

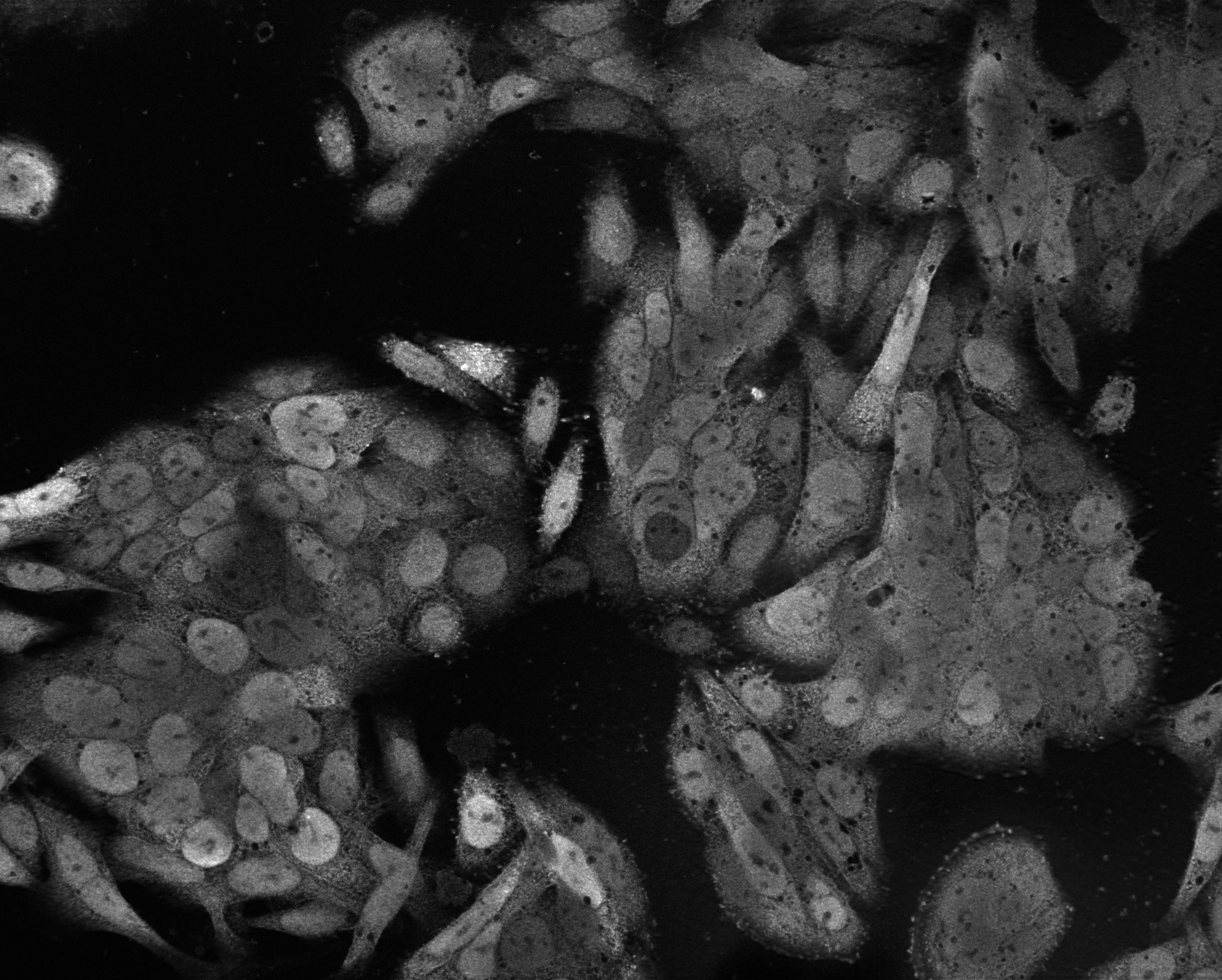

Supplement: Supplementary file 7 — Source data Fig. 5 [file 44321_2024_66_MOESM7_ESM.zip › Figure 5/5D/IF/KOSW480 IFNg STAT1Green CBX3Red 002.tif_files/C004sw480ifn stat1Vcbx3R 002_h0b0c1x0-2752y0-2208.tif]

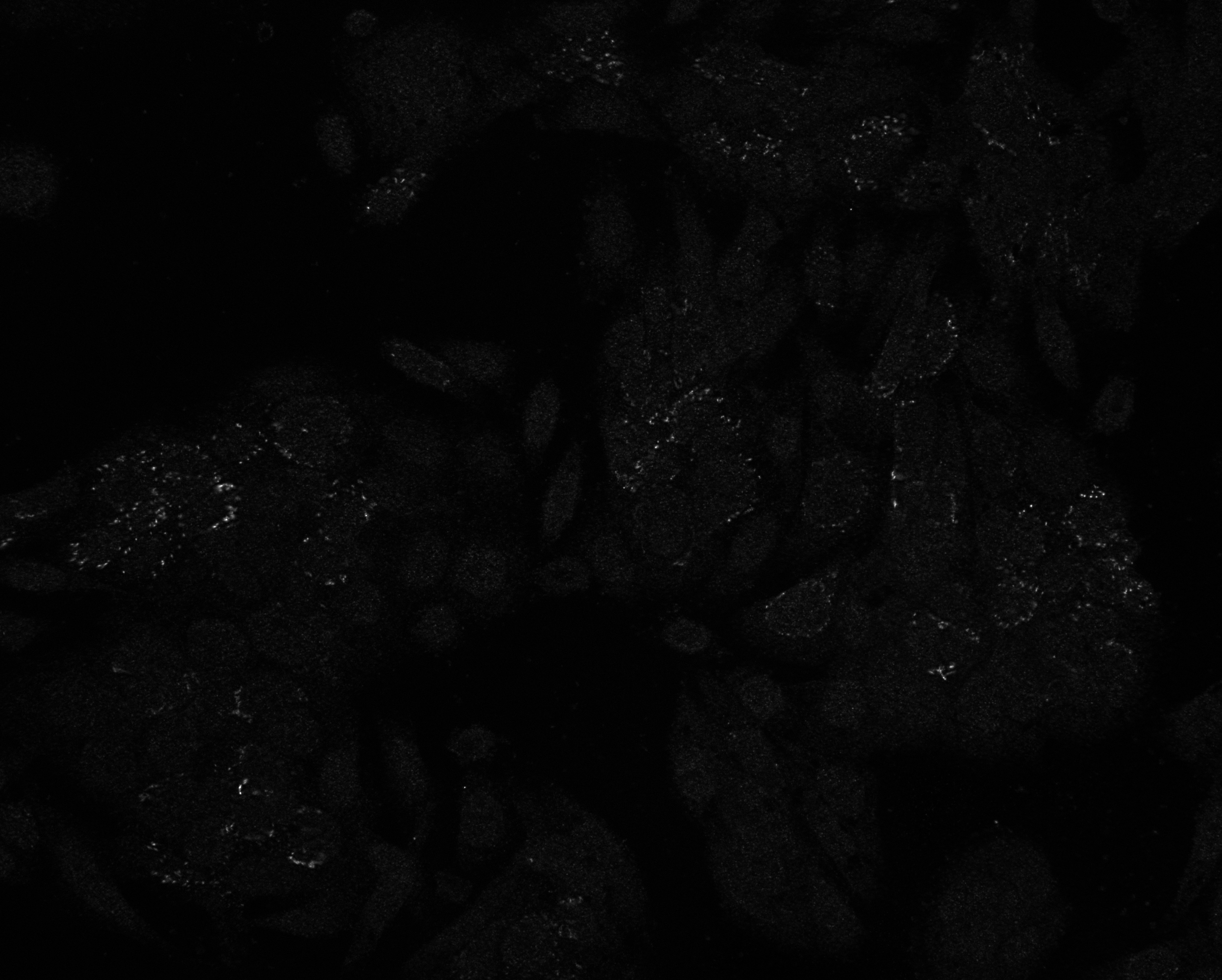

Supplement: Supplementary file 7 — Source data Fig. 5 [file 44321_2024_66_MOESM7_ESM.zip › Figure 5/5D/IF/KOSW480 IFNg STAT1Green CBX3Red 002.tif_files/C004sw480ifn stat1Vcbx3R 002_h0b0c2x0-2752y0-2208.tif]

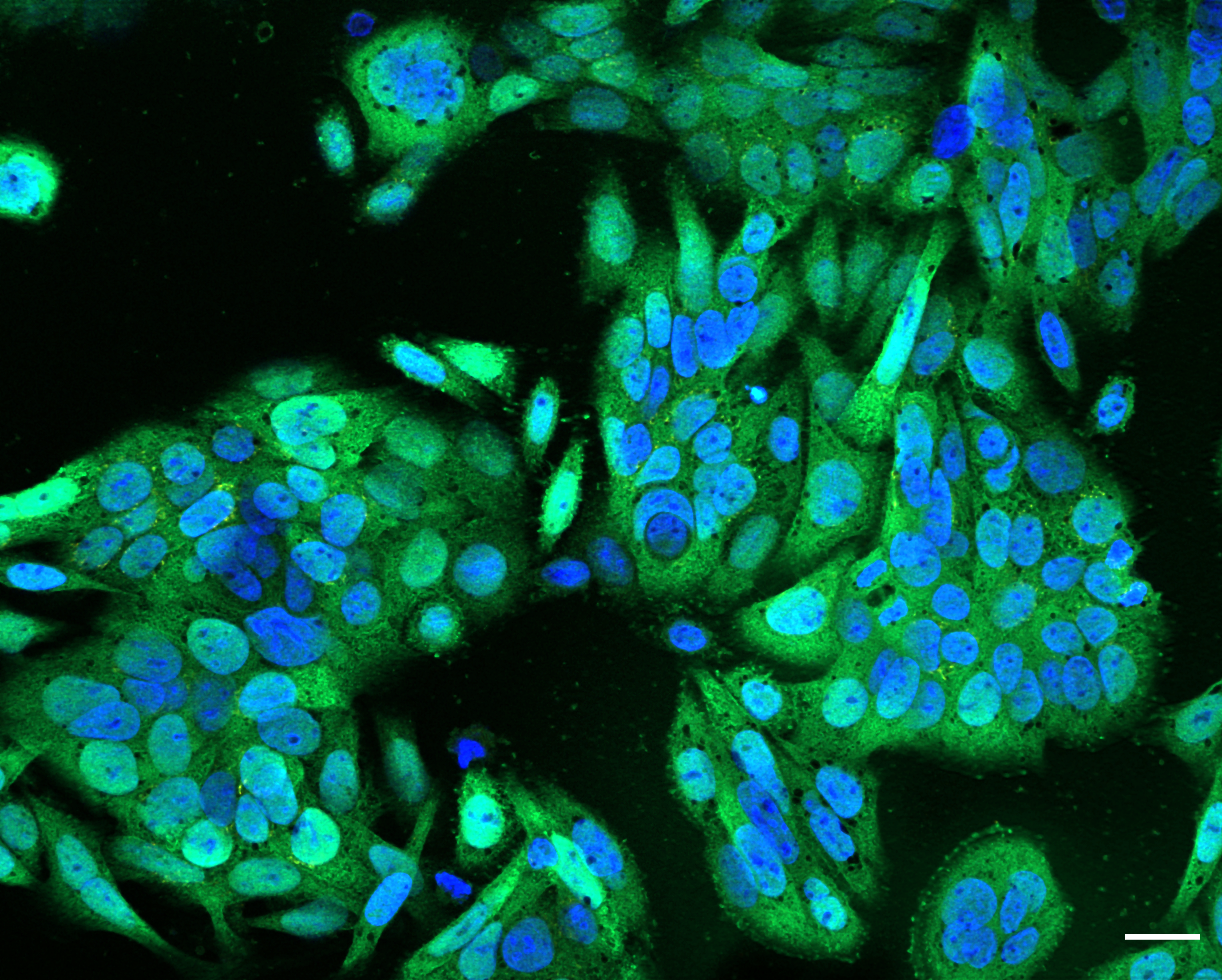

Supplement: Supplementary file 7 — Source data Fig. 5 [file 44321_2024_66_MOESM7_ESM.zip › Figure 5/5D/IF/KOSW480 IFNg STAT1Green CBX3Red 002.tif_files/SW480 CBX3 KO IFNg STAT1 Green CBX3red.tif]

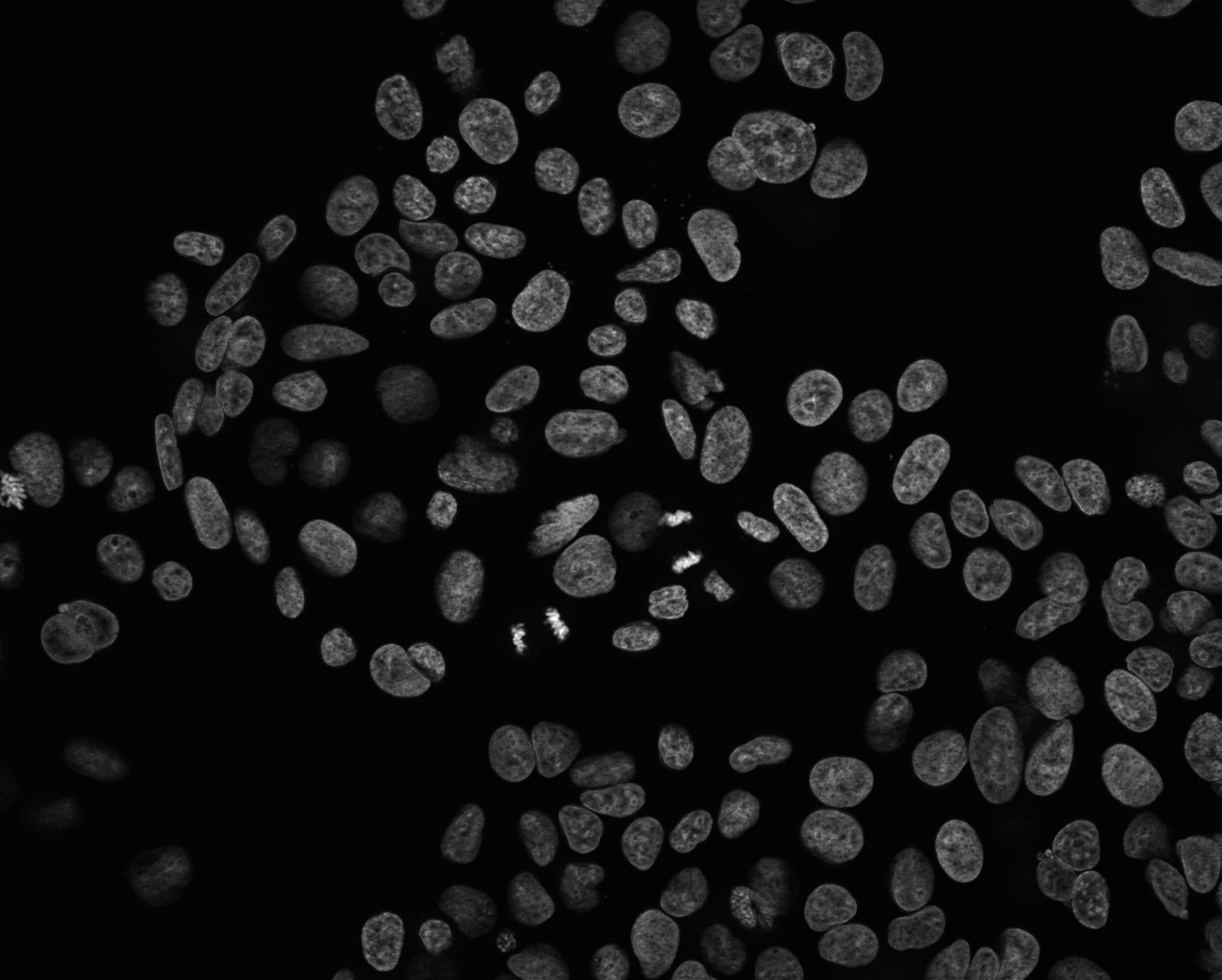

Supplement: Supplementary file 7 — Source data Fig. 5 [file 44321_2024_66_MOESM7_ESM.zip › Figure 5/5D/IF/KOSW480Ctrl STAT1Green CBX3Red.tif_files/C006SW480Ctrl Stat1Vcbx3R_h0b0c0x0-2752y0-2208.tif]

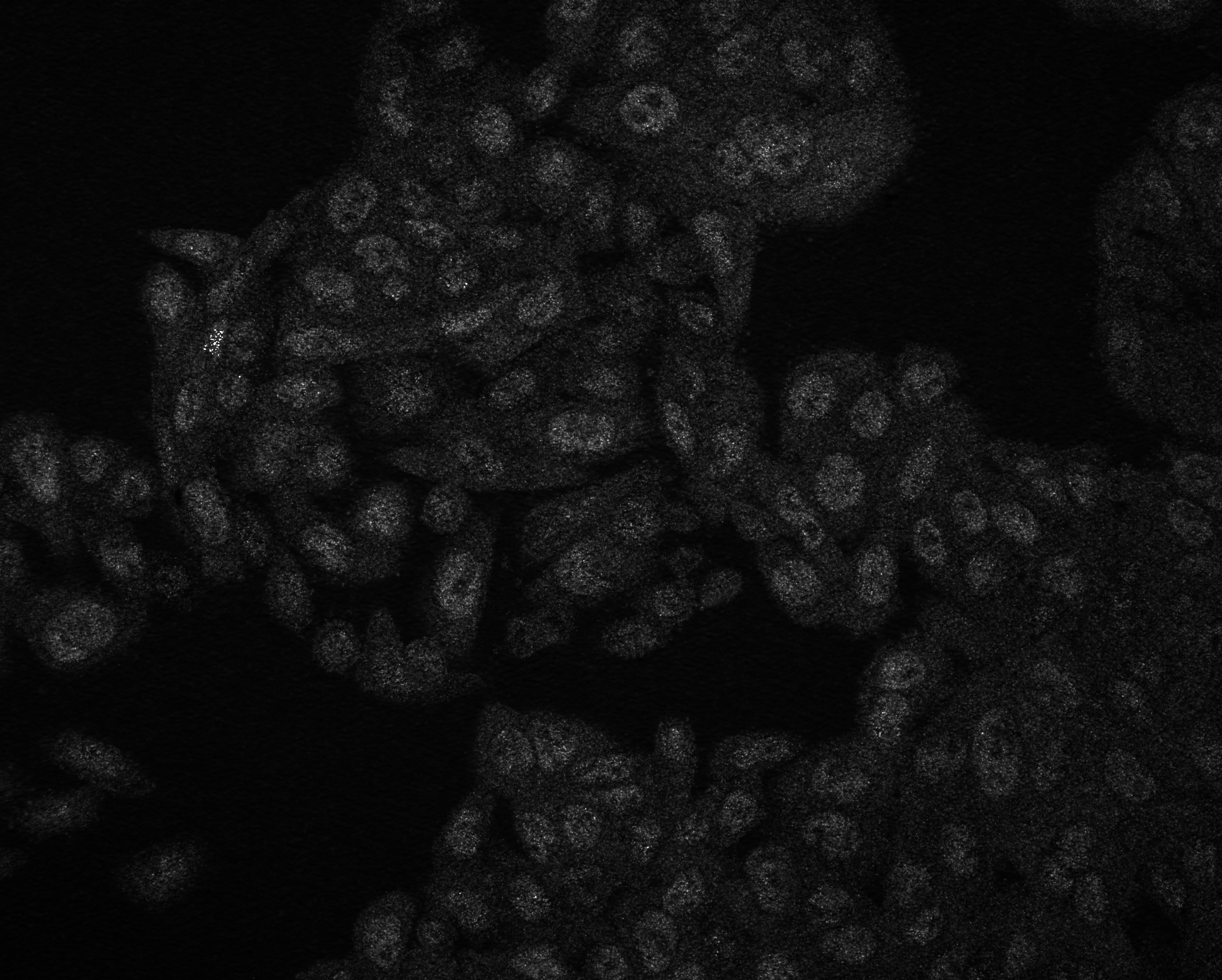

Supplement: Supplementary file 7 — Source data Fig. 5 [file 44321_2024_66_MOESM7_ESM.zip › Figure 5/5D/IF/KOSW480Ctrl STAT1Green CBX3Red.tif_files/C006SW480Ctrl Stat1Vcbx3R_h0b0c1x0-2752y0-2208.tif]

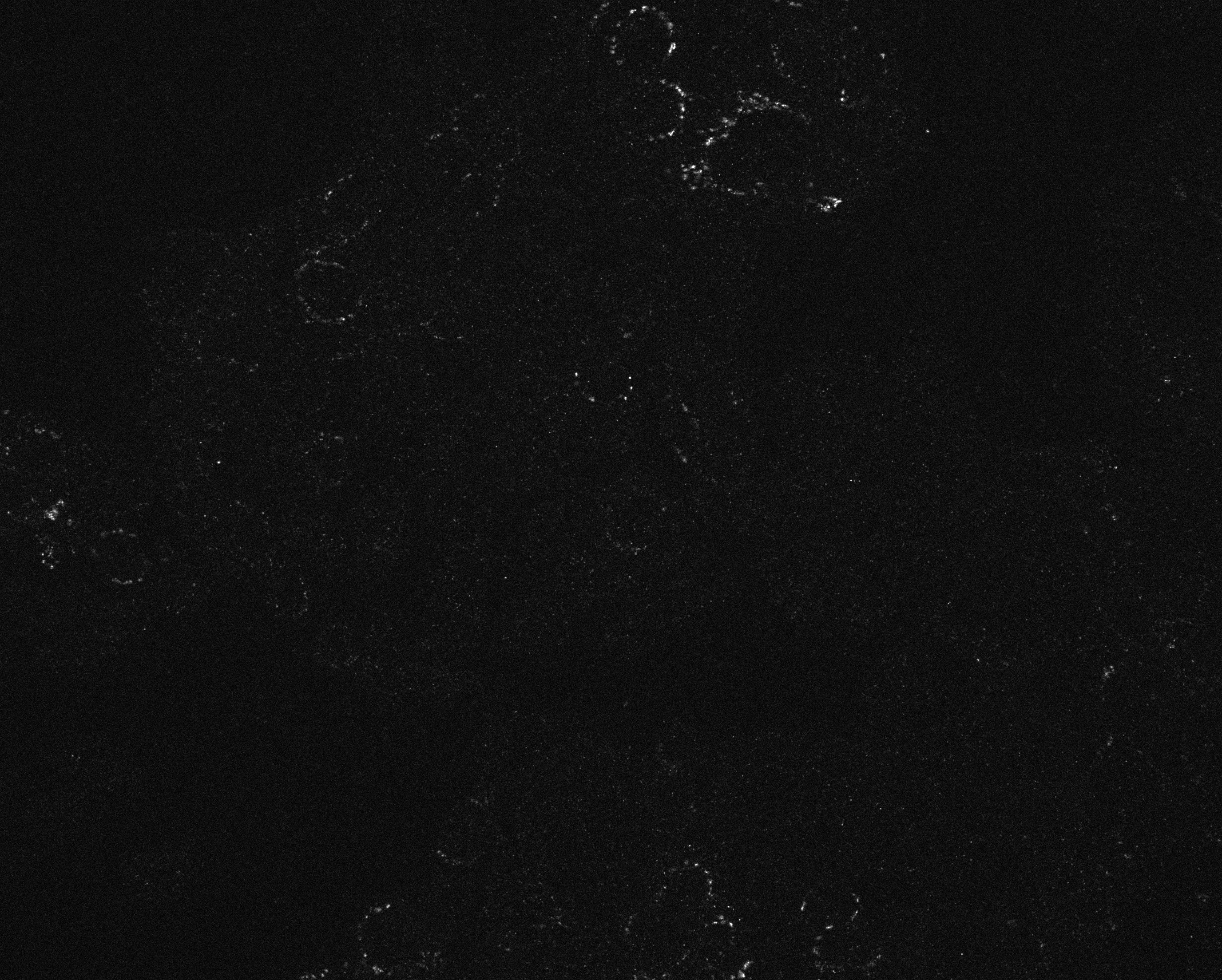

Supplement: Supplementary file 7 — Source data Fig. 5 [file 44321_2024_66_MOESM7_ESM.zip › Figure 5/5D/IF/KOSW480Ctrl STAT1Green CBX3Red.tif_files/C006SW480Ctrl Stat1Vcbx3R_h0b0c2x0-2752y0-2208.tif]

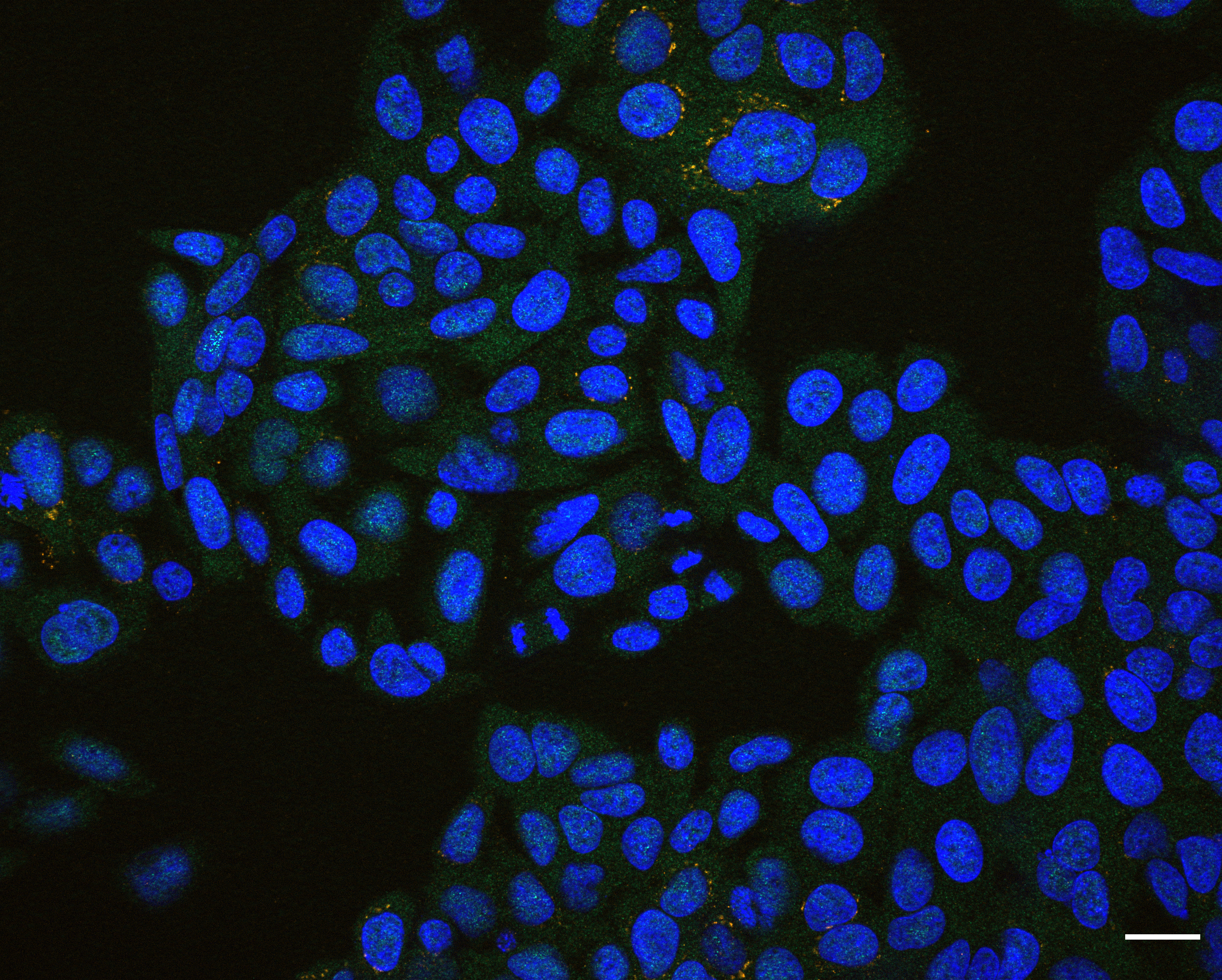

Supplement: Supplementary file 7 — Source data Fig. 5 [file 44321_2024_66_MOESM7_ESM.zip › Figure 5/5D/IF/KOSW480Ctrl STAT1Green CBX3Red.tif_files/SW480 CBX3KOSTAT1 green CBX3 red.tif]

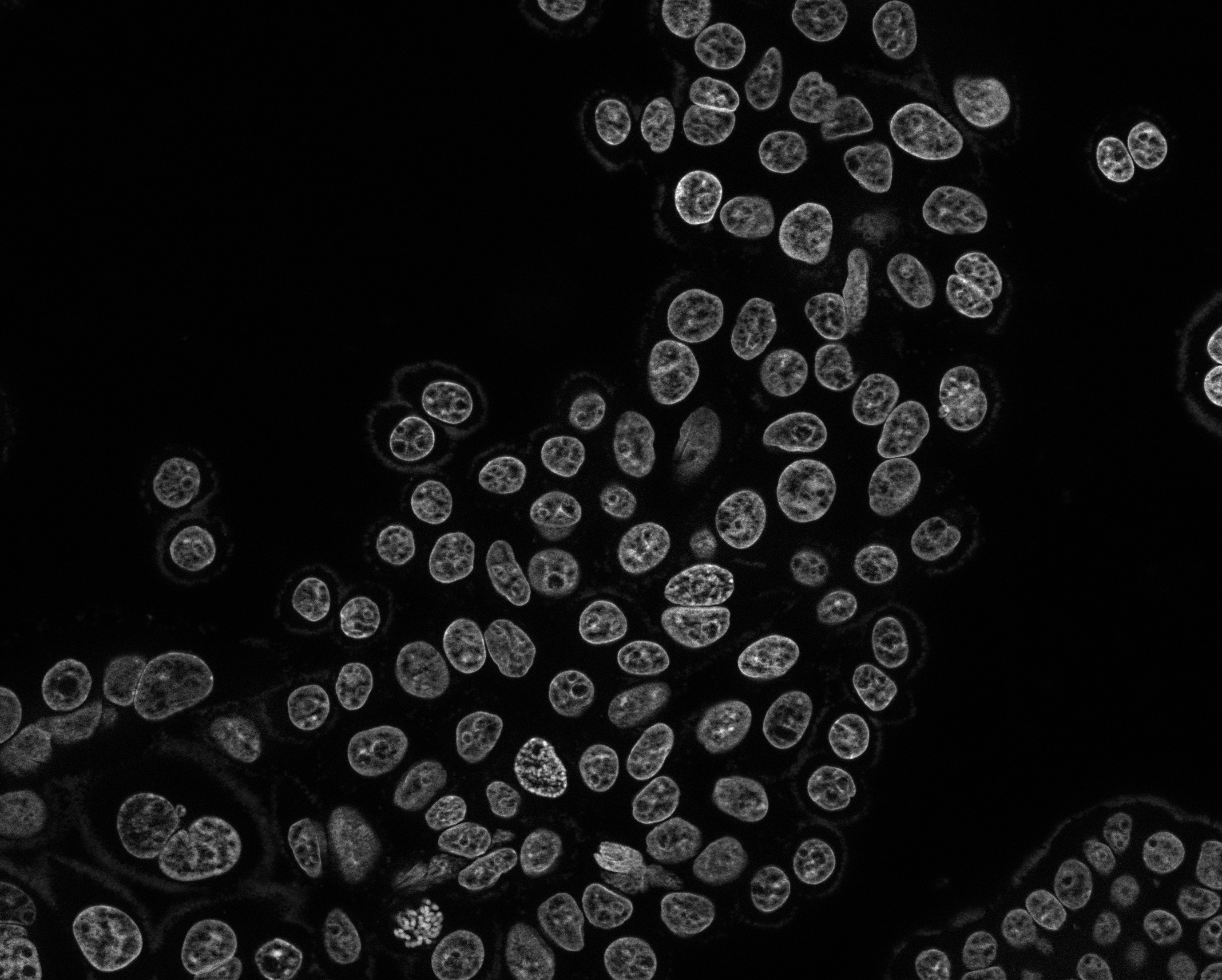

Supplement: Supplementary file 7 — Source data Fig. 5 [file 44321_2024_66_MOESM7_ESM.zip › Figure 5/5D/IF/SW480 WT CTRL STAT1 Green CBX3 Red.tif_files/SW480 WT CTRL STAT1 GREEN HP1GAMMA RED BIS_h0b0c0x0-2752y0-2208.tif]

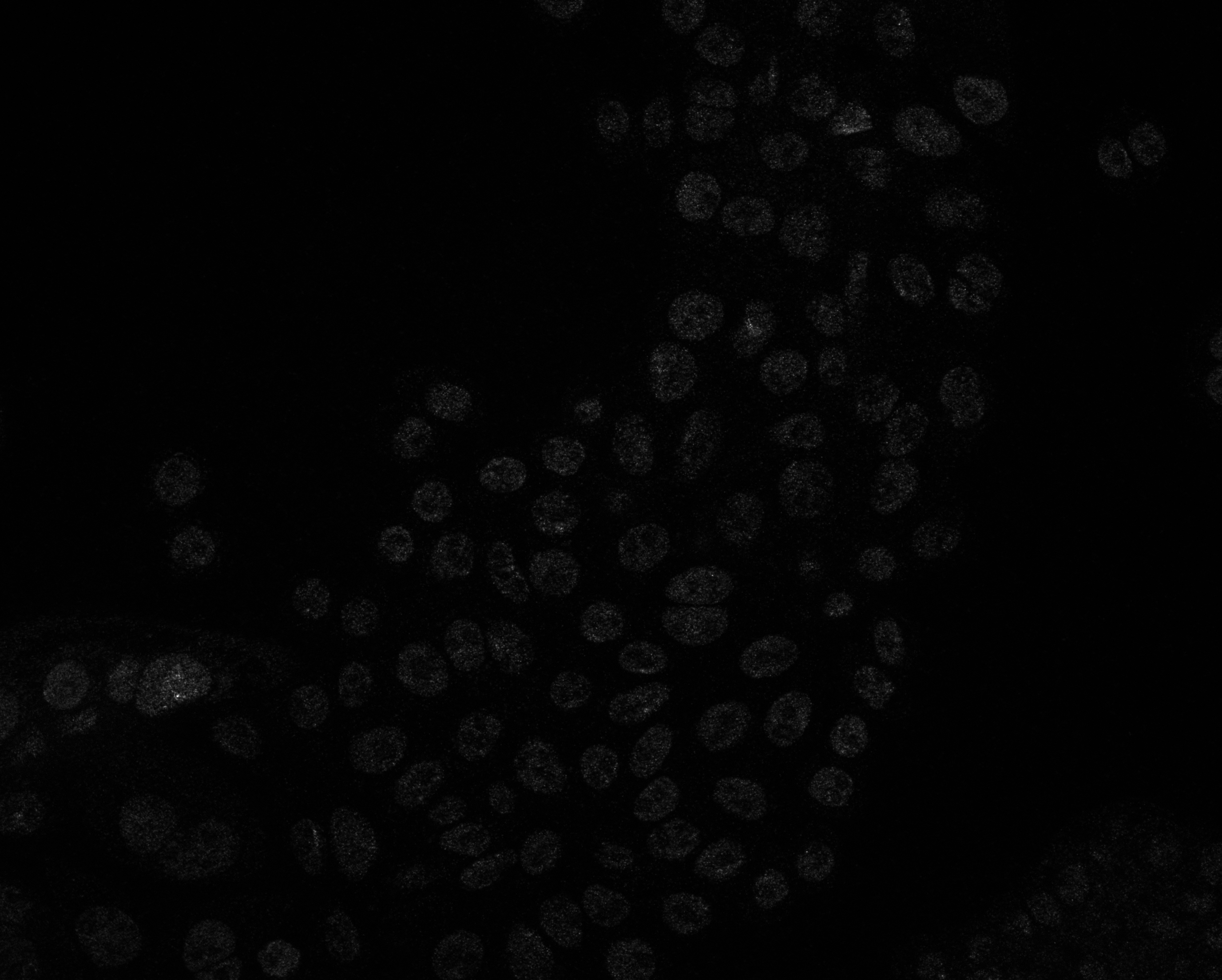

Supplement: Supplementary file 7 — Source data Fig. 5 [file 44321_2024_66_MOESM7_ESM.zip › Figure 5/5D/IF/SW480 WT CTRL STAT1 Green CBX3 Red.tif_files/SW480 WT CTRL STAT1 GREEN HP1GAMMA RED BIS_h0b0c1x0-2752y0-2208.tif]

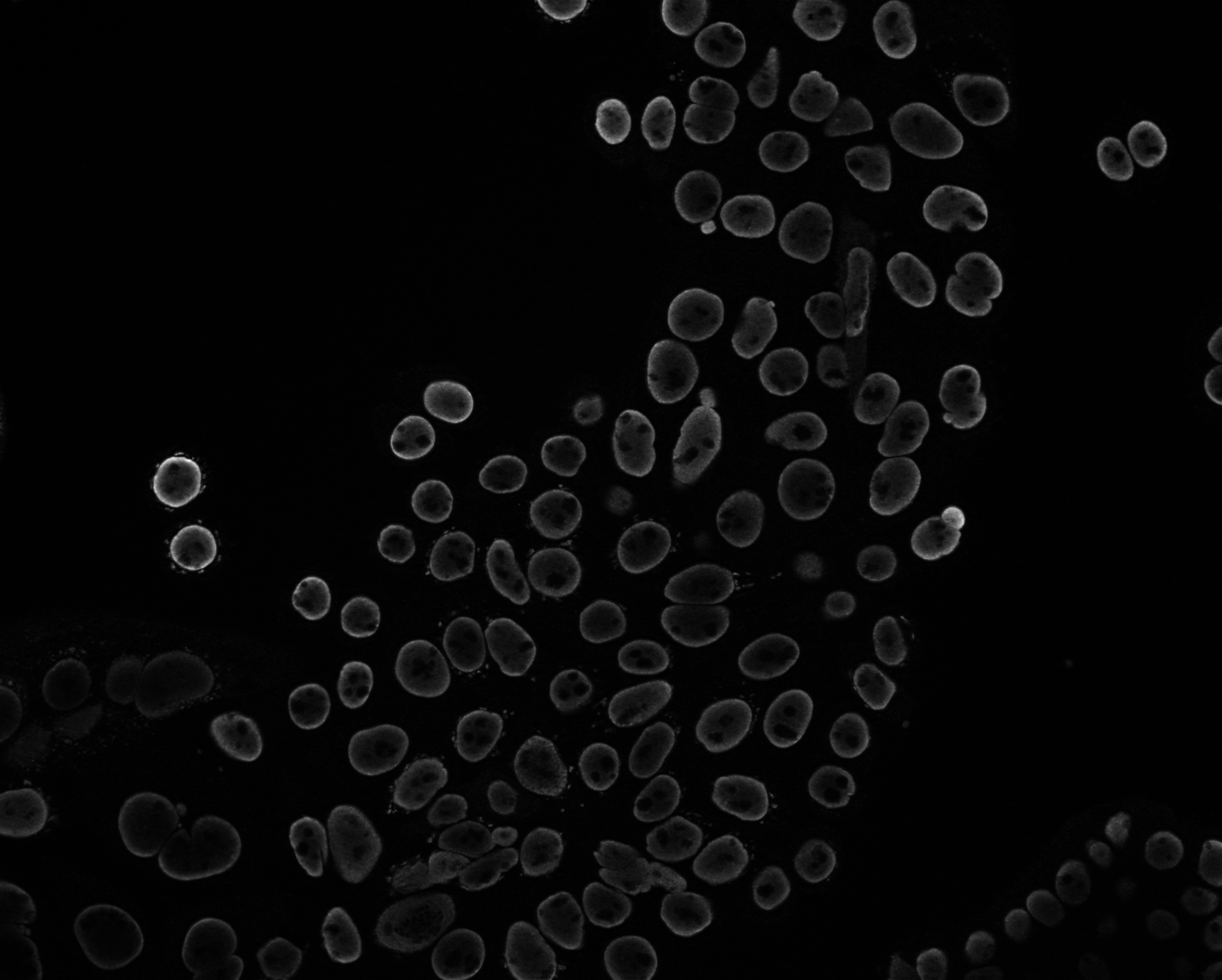

Supplement: Supplementary file 7 — Source data Fig. 5 [file 44321_2024_66_MOESM7_ESM.zip › Figure 5/5D/IF/SW480 WT CTRL STAT1 Green CBX3 Red.tif_files/SW480 WT CTRL STAT1 GREEN HP1GAMMA RED BIS_h0b0c2x0-2752y0-2208.tif]

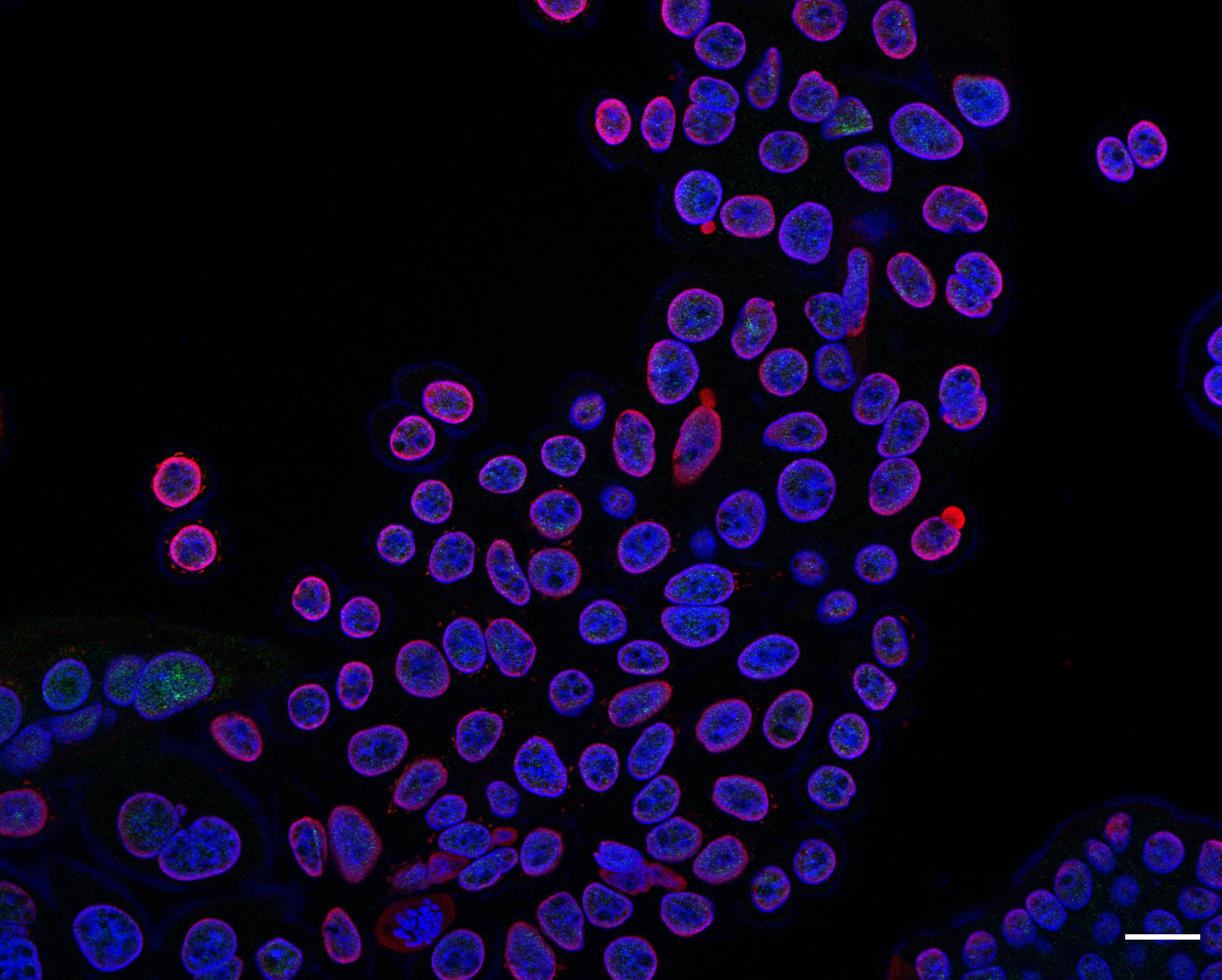

Supplement: Supplementary file 7 — Source data Fig. 5 [file 44321_2024_66_MOESM7_ESM.zip › Figure 5/5D/IF/SW480 WT CTRL STAT1 Green CBX3 Red.tif_files/SW480 WT CTRL STAT1 GREEN HP1GAMMA RED BIS-Bar.tif]

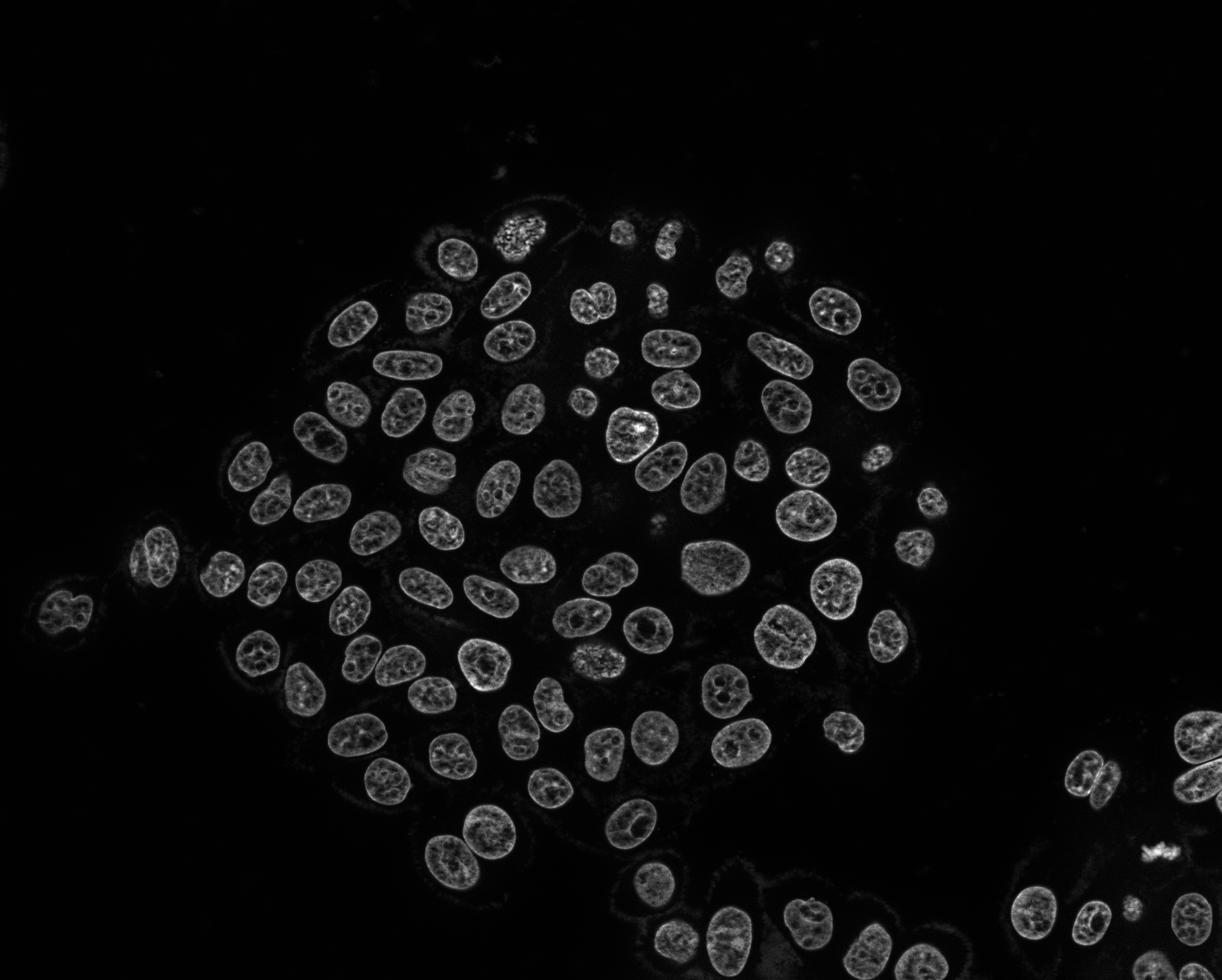

Supplement: Supplementary file 7 — Source data Fig. 5 [file 44321_2024_66_MOESM7_ESM.zip › Figure 5/5D/IF/WT SW480 IFNg STAT1 Green CBX3Red.tif_files/SW480 WT 200U IFN STAT1 GREEN HP1GAMMA RED_h0b0c0x0-2752y0-2208.tif]

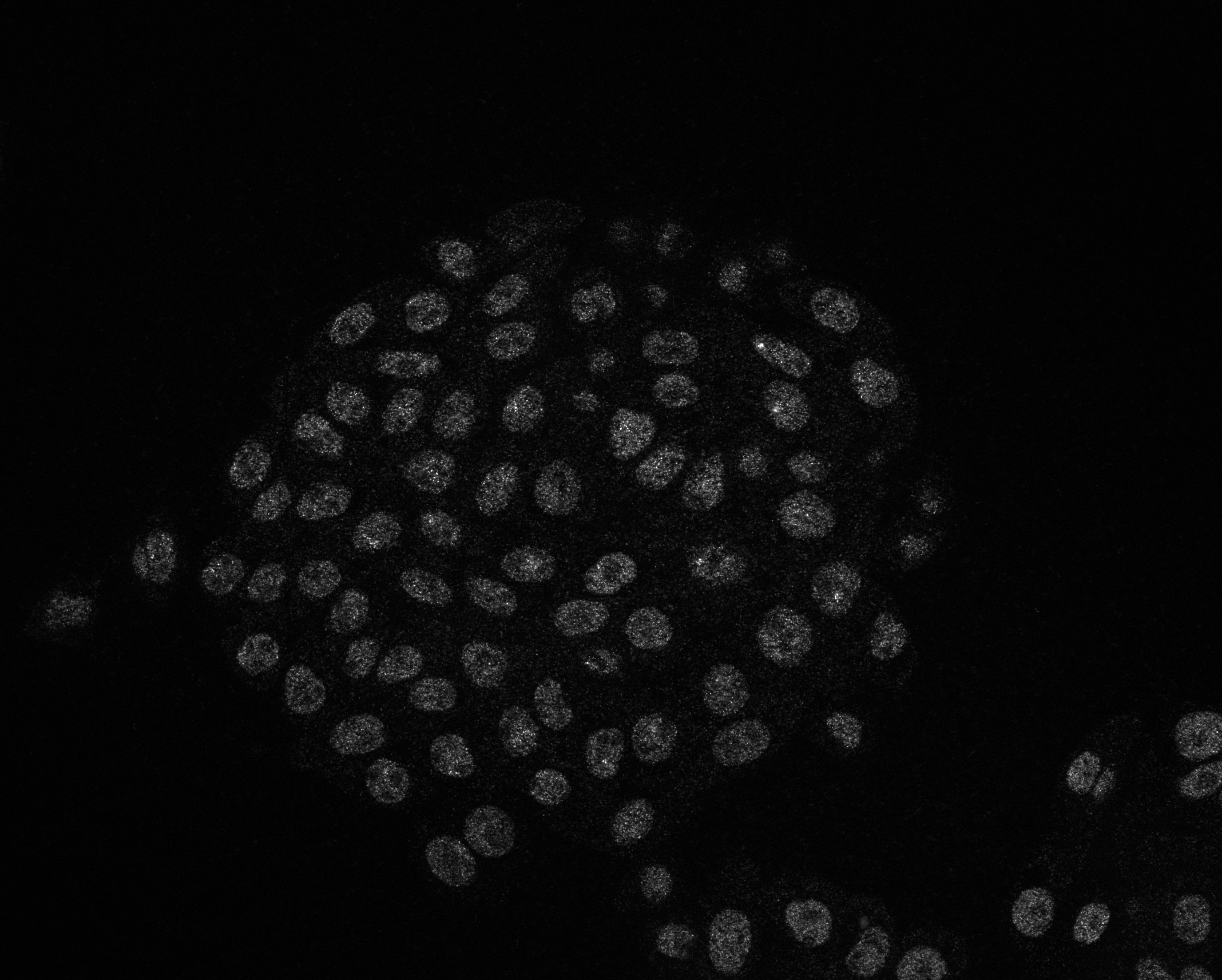

Supplement: Supplementary file 7 — Source data Fig. 5 [file 44321_2024_66_MOESM7_ESM.zip › Figure 5/5D/IF/WT SW480 IFNg STAT1 Green CBX3Red.tif_files/SW480 WT 200U IFN STAT1 GREEN HP1GAMMA RED_h0b0c1x0-2752y0-2208.tif]
