# Supplementary figures and images for: CBX3 antagonizes IFNγ/STAT1/PD-L1 axis to modulate colon inflammation and CRC chemosensitivity (part 2 of 2)
Source: EMBO Mol Med. 2024 Apr 29;16(6):10. doi: 10.1038/s44321-024-00066-6 (PMC11178889; doi:10.1038/s44321-024-00066-6)

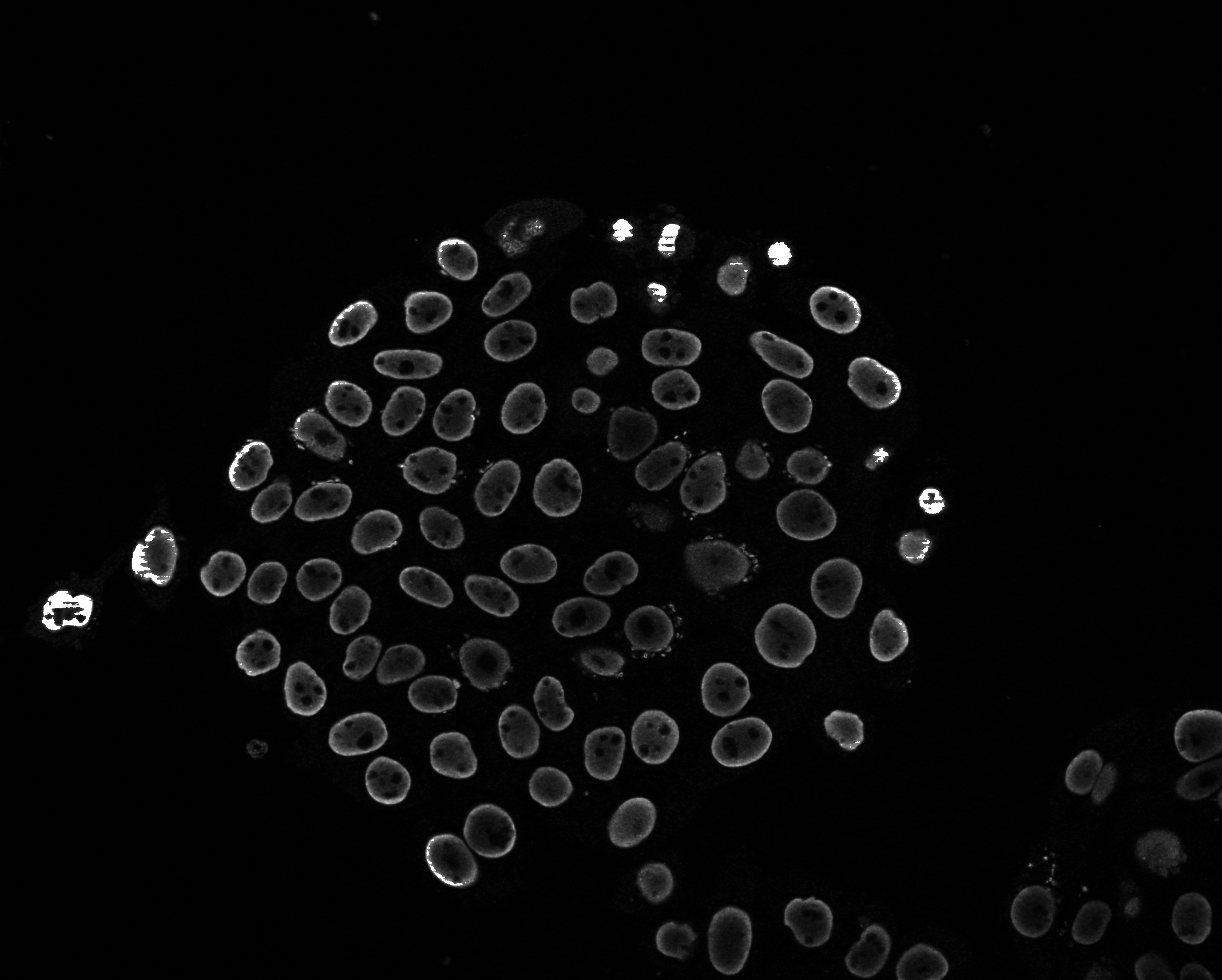

Supplement: Supplementary file 7 — Source data Fig. 5 [file 44321_2024_66_MOESM7_ESM.zip › Figure 5/5D/IF/WT SW480 IFNg STAT1 Green CBX3Red.tif_files/SW480 WT 200U IFN STAT1 GREEN HP1GAMMA RED_h0b0c2x0-2752y0-2208.tif]

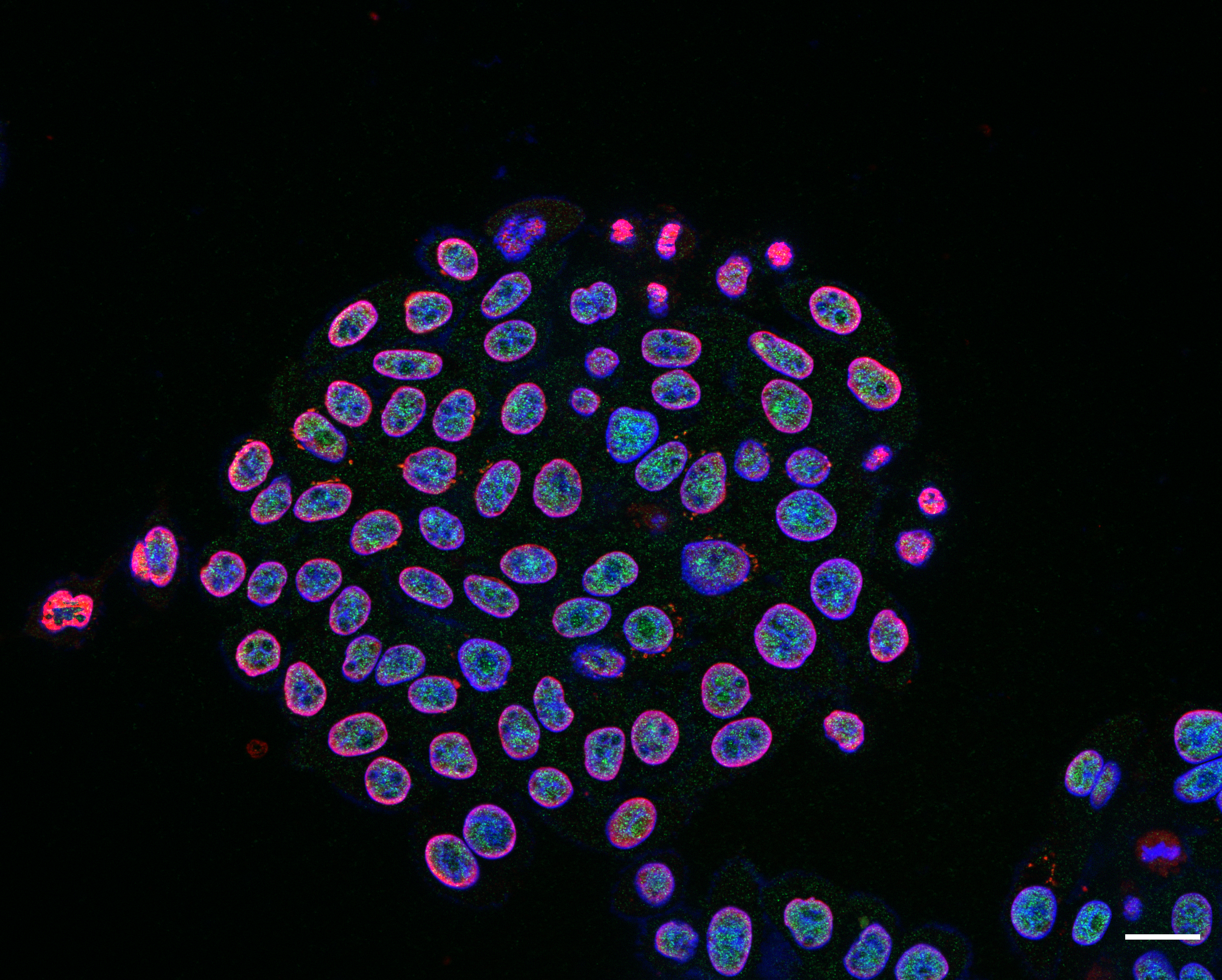

Supplement: Supplementary file 7 — Source data Fig. 5 [file 44321_2024_66_MOESM7_ESM.zip › Figure 5/5D/IF/WT SW480 IFNg STAT1 Green CBX3Red.tif_files/SW480 WT 200U IFN STAT1 GREEN HP1GAMMA RED-Bar.tif]

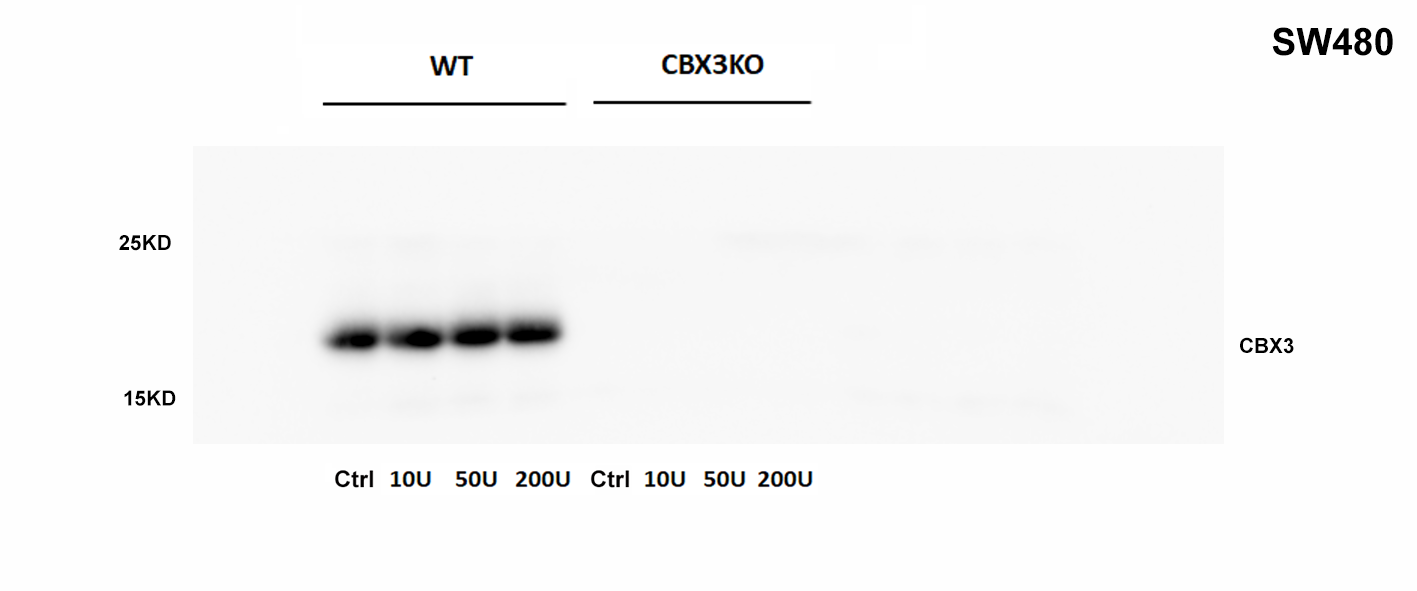

Supplement: Supplementary file 7 — Source data Fig. 5 [file 44321_2024_66_MOESM7_ESM.zip › Figure 5/5D/western/HP1gamma SW480 WT KO_5(Chemiluminescence).tif]

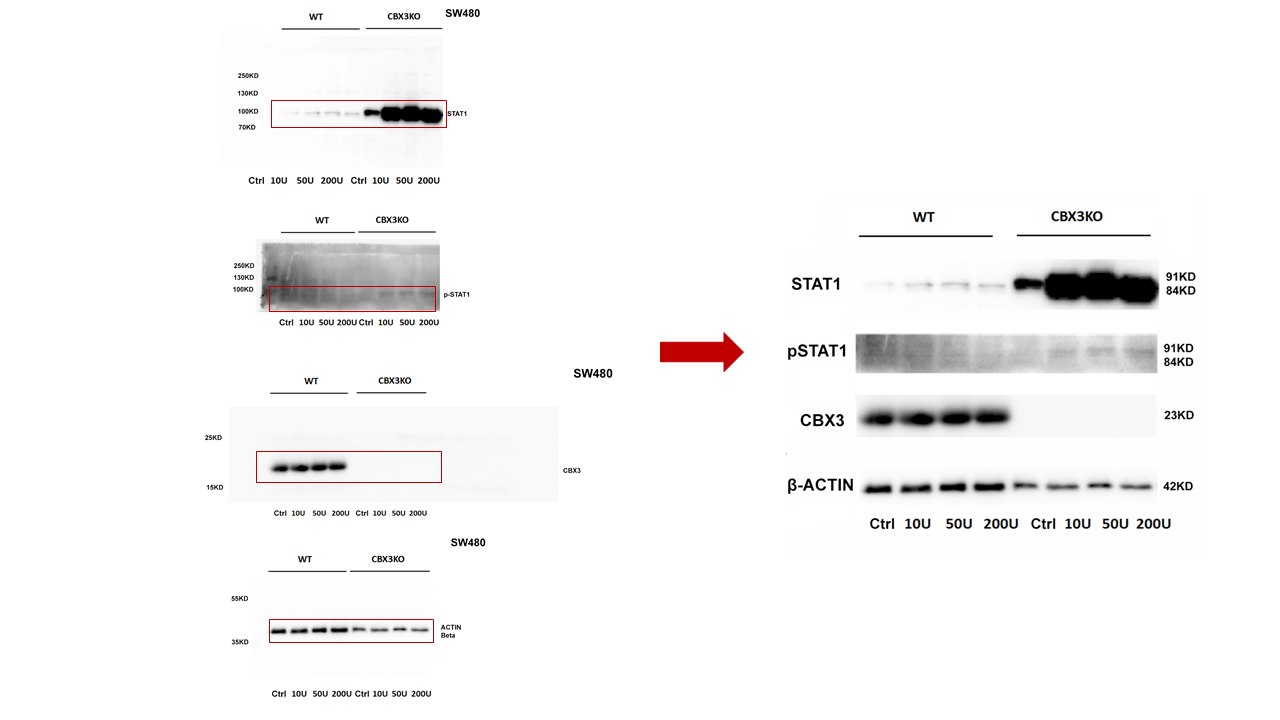

Supplement: Supplementary file 7 — Source data Fig. 5 [file 44321_2024_66_MOESM7_ESM.zip › Figure 5/5D/western/resume SW480.tif]

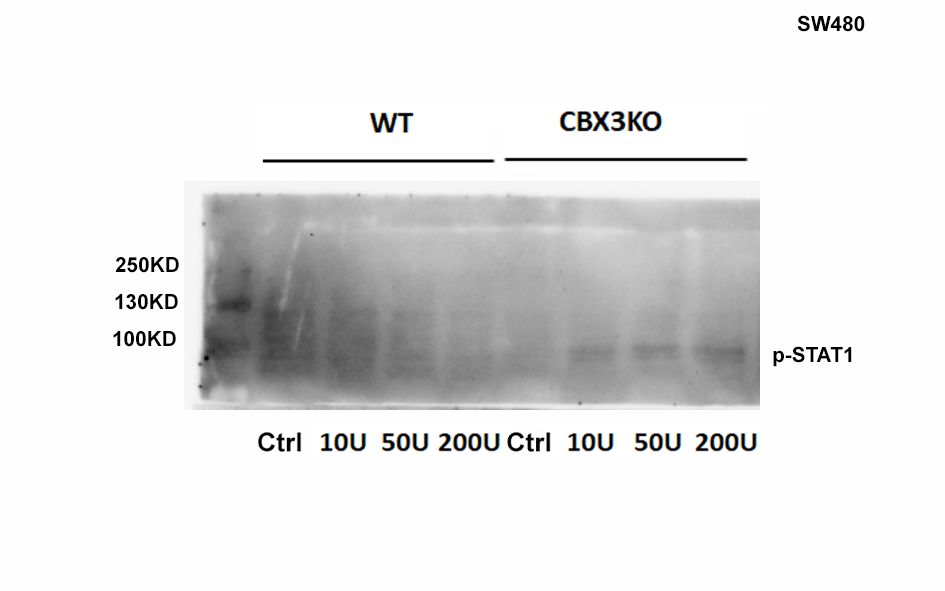

Supplement: Supplementary file 7 — Source data Fig. 5 [file 44321_2024_66_MOESM7_ESM.zip › Figure 5/5D/western/SW480 p-stat1 WT KO_4(Chemiluminescence).tif]

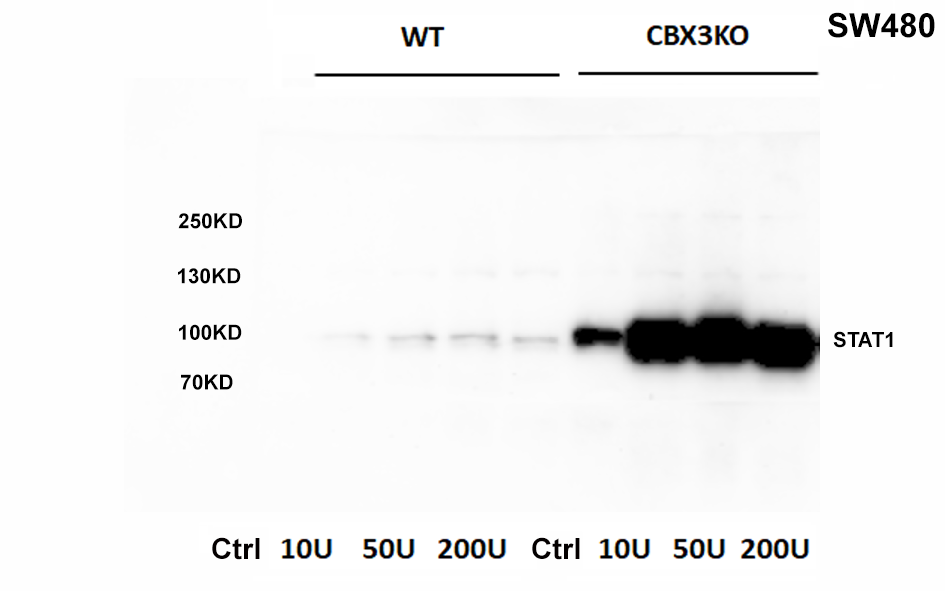

Supplement: Supplementary file 7 — Source data Fig. 5 [file 44321_2024_66_MOESM7_ESM.zip › Figure 5/5D/western/SW480 STAT1 WT KO.tif]

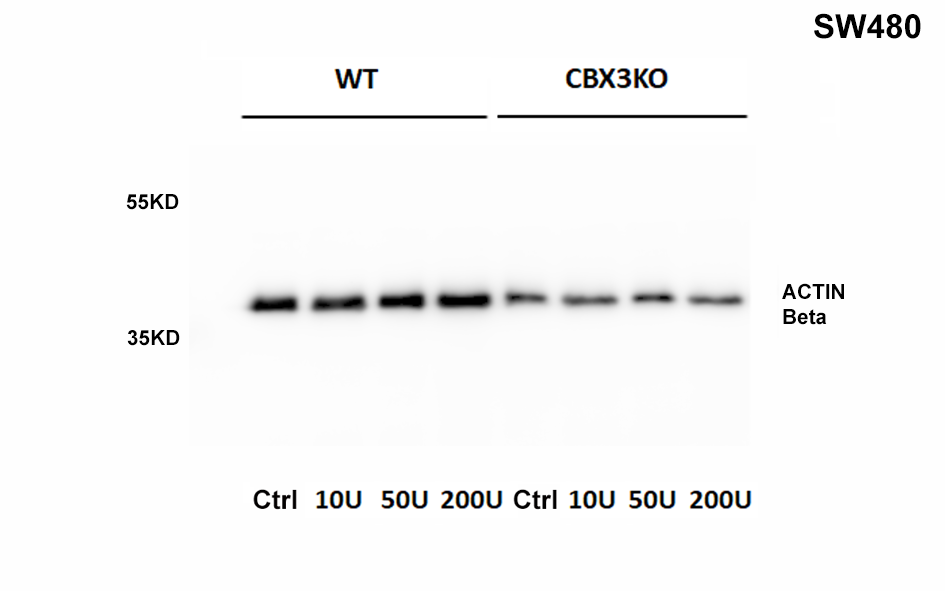

Supplement: Supplementary file 7 — Source data Fig. 5 [file 44321_2024_66_MOESM7_ESM.zip › Figure 5/5D/western/SW480 tubulinWT KO_1(Chemiluminescence).tif]

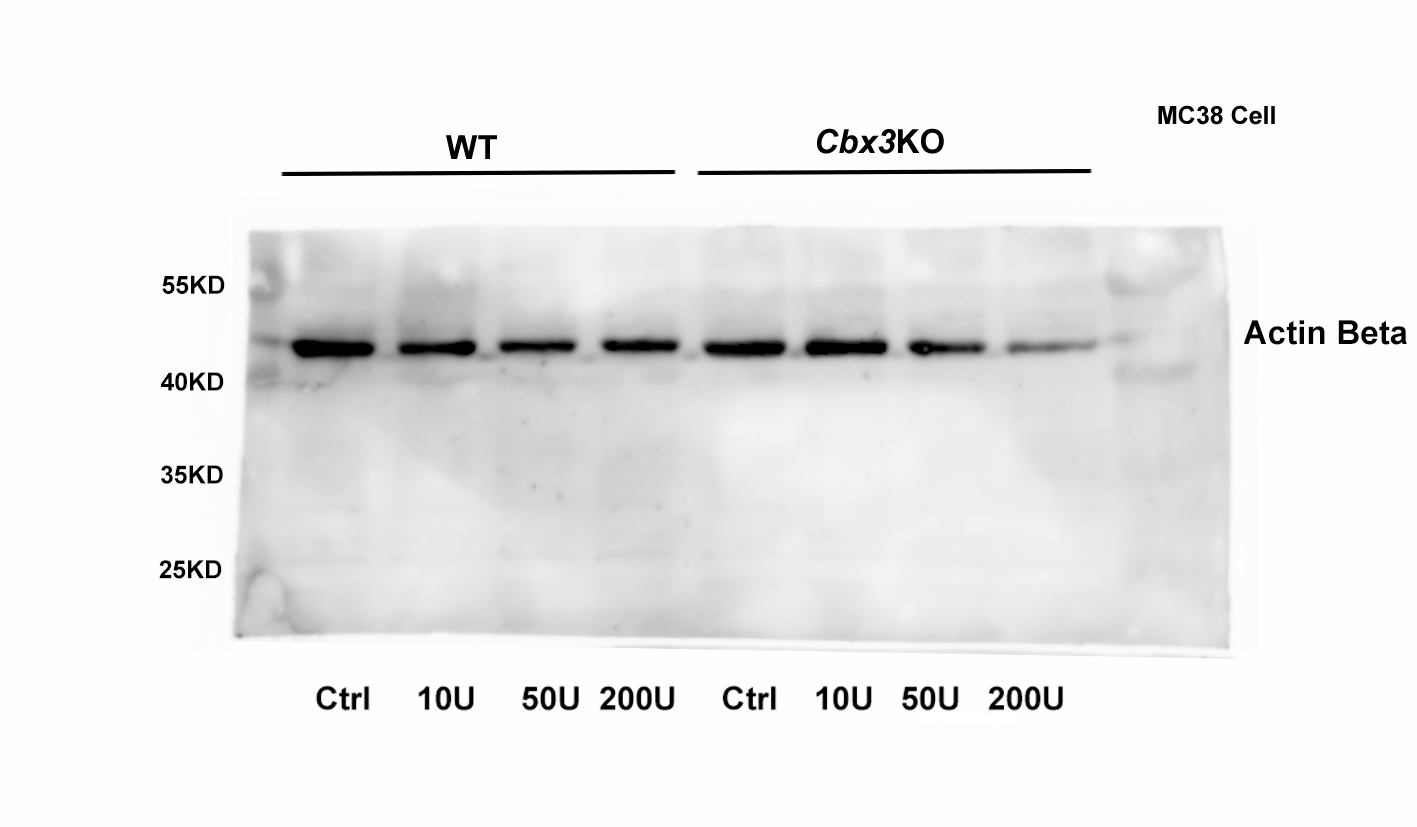

Supplement: Supplementary file 11 — Source data Fig. 9 [file 44321_2024_66_MOESM11_ESM.zip › Figure 9/9G/MC38 WT KO Actin beta.tif]

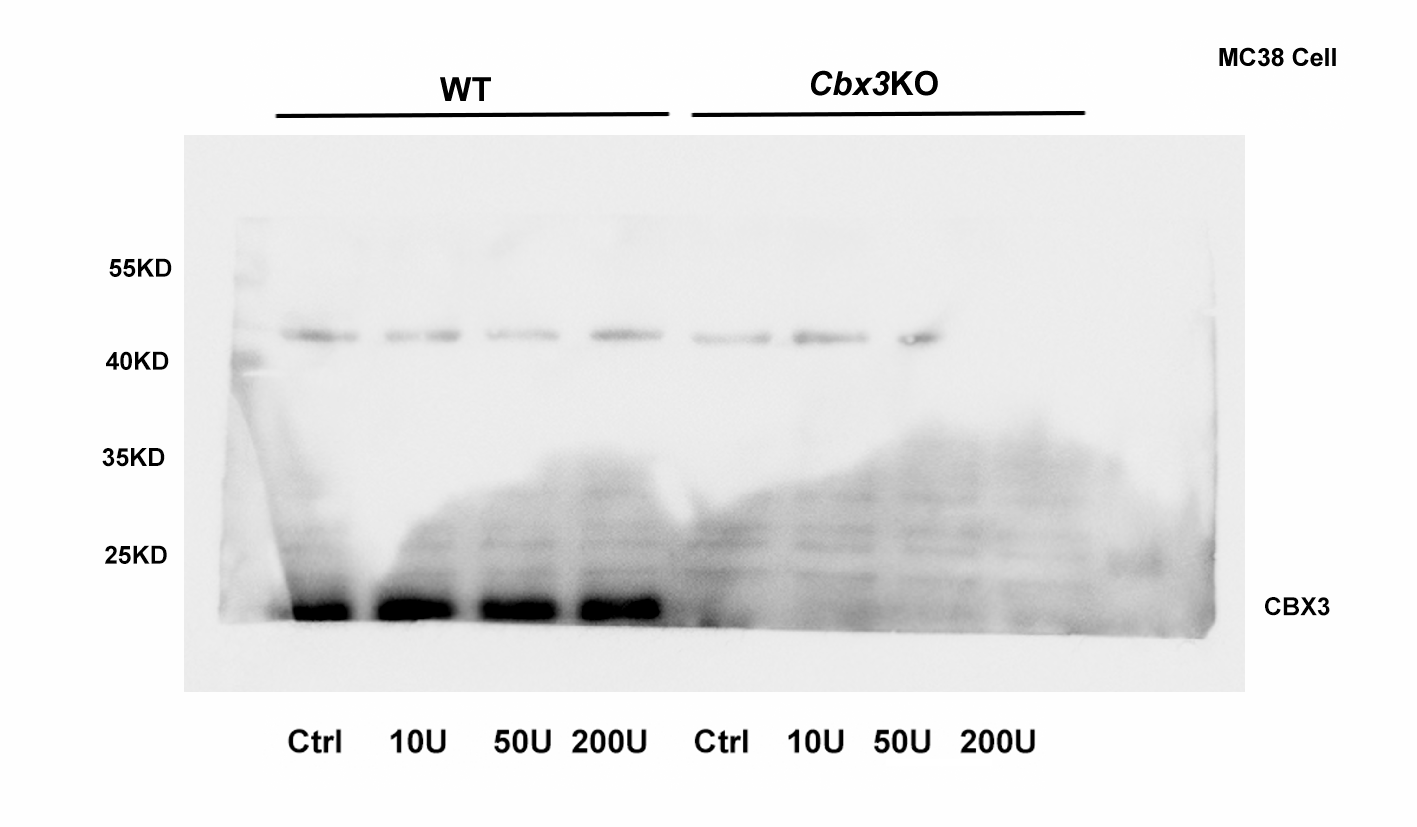

Supplement: Supplementary file 11 — Source data Fig. 9 [file 44321_2024_66_MOESM11_ESM.zip › Figure 9/9G/MC38 WT KO HP1gamma.tif]

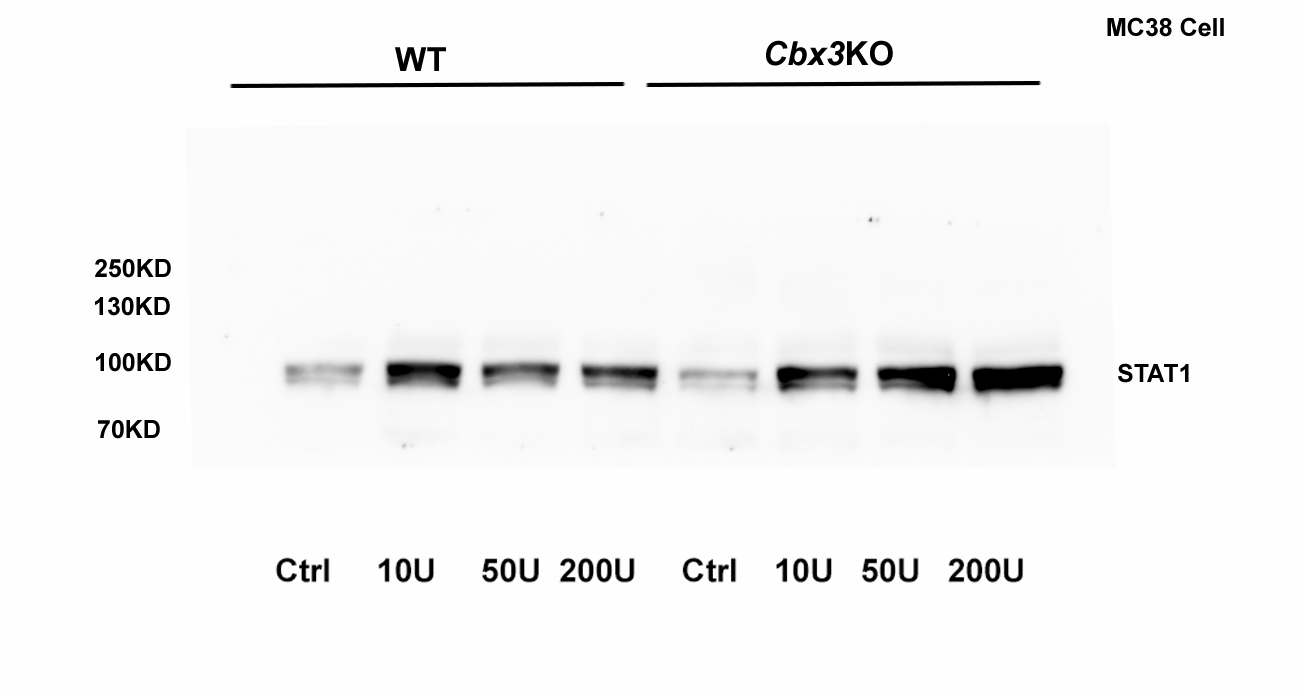

Supplement: Supplementary file 11 — Source data Fig. 9 [file 44321_2024_66_MOESM11_ESM.zip › Figure 9/9G/MC38 WT KO STAT1.tif]

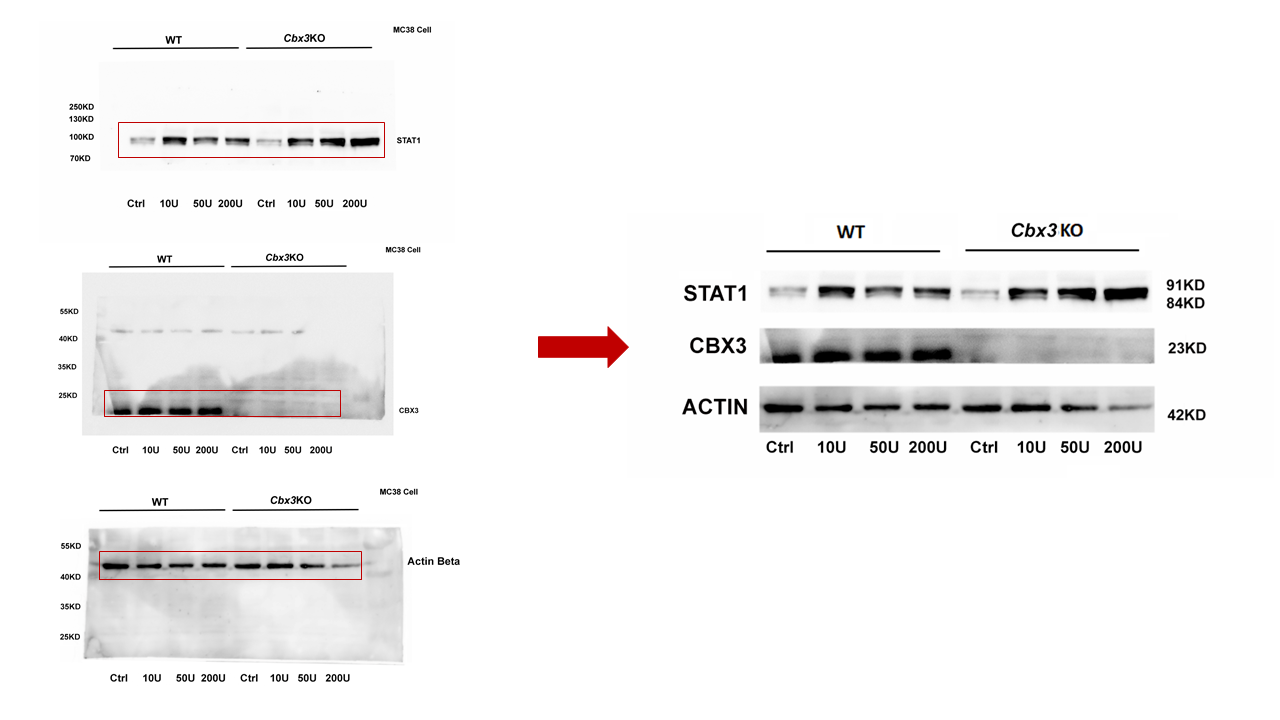

Supplement: Supplementary file 11 — Source data Fig. 9 [file 44321_2024_66_MOESM11_ESM.zip › Figure 9/9G/resume.tif]

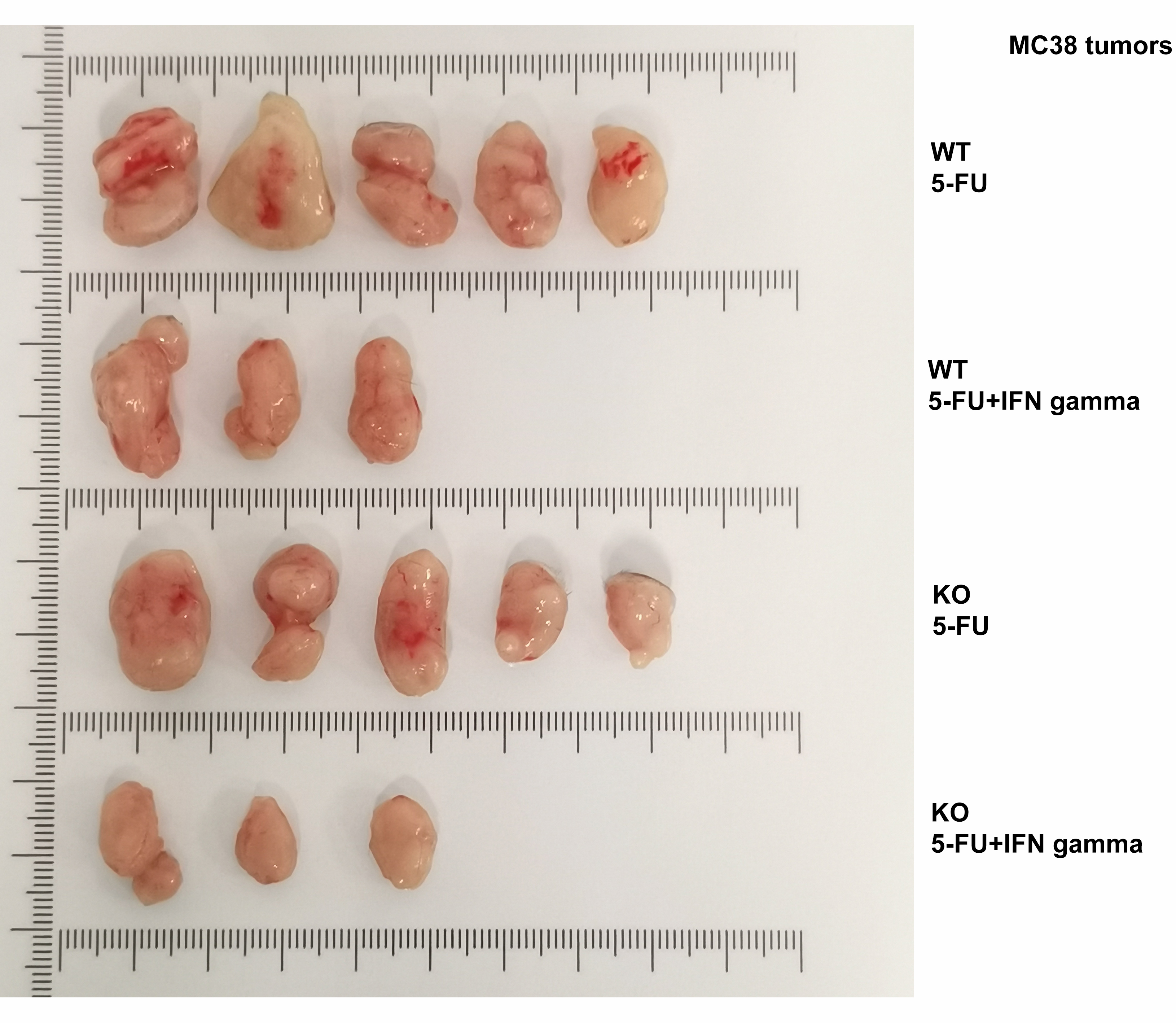

Supplement: Supplementary file 11 — Source data Fig. 9 [file 44321_2024_66_MOESM11_ESM.zip › Figure 9/9I/MC38 Tumors (2).jpg]

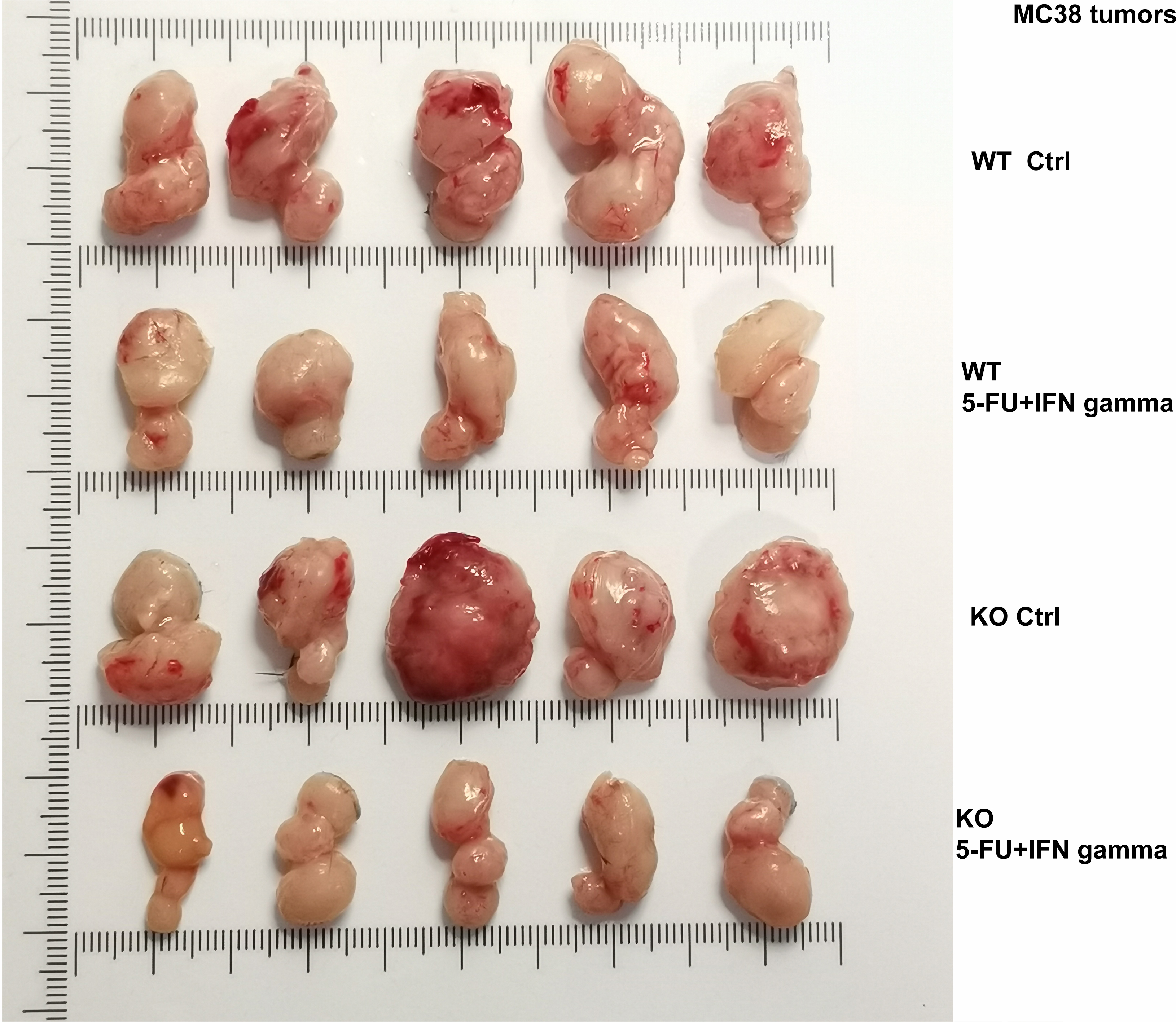

Supplement: Supplementary file 11 — Source data Fig. 9 [file 44321_2024_66_MOESM11_ESM.zip › Figure 9/9I/MC38 tumors(1).jpg]

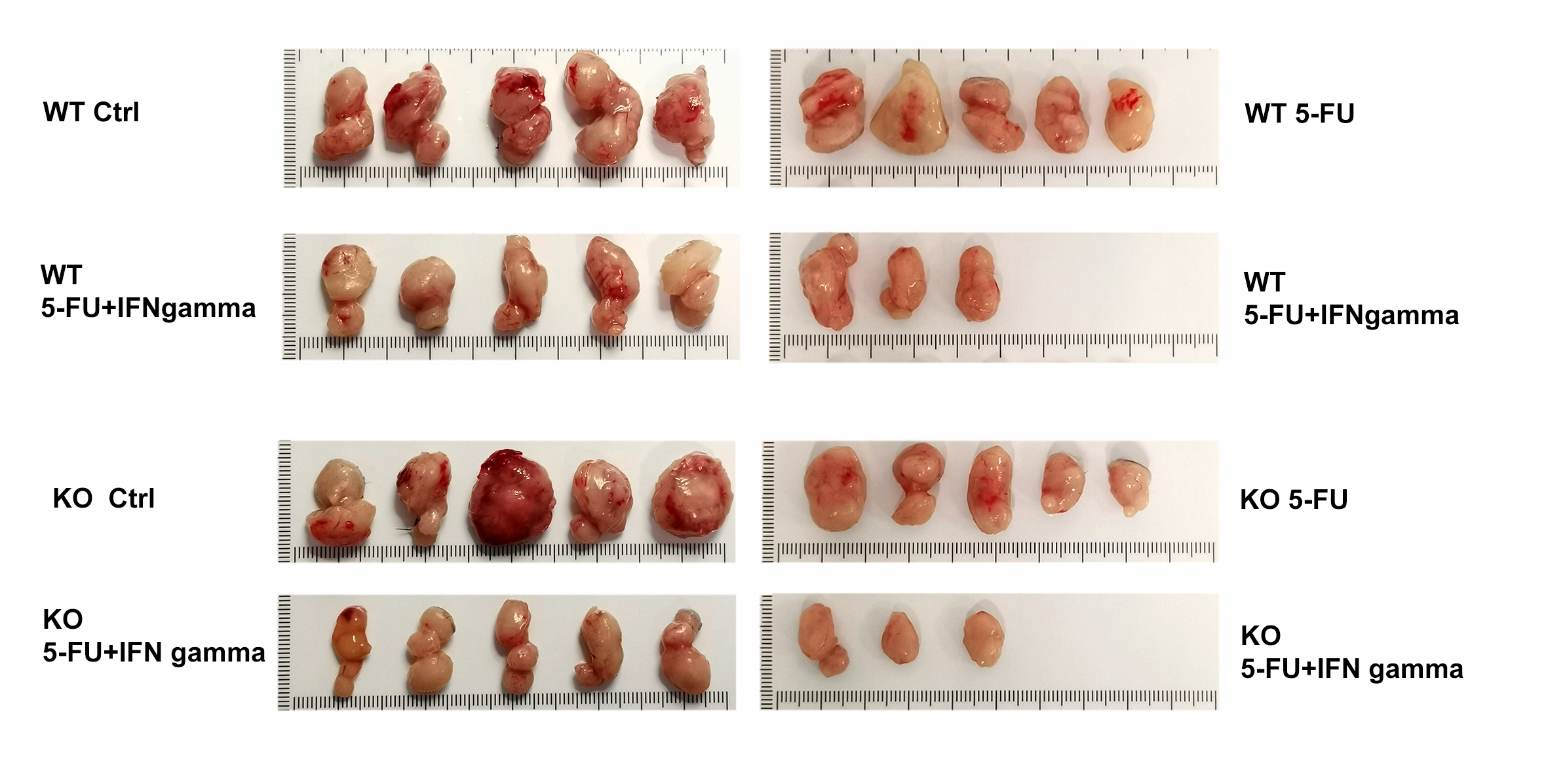

Supplement: Supplementary file 11 — Source data Fig. 9 [file 44321_2024_66_MOESM11_ESM.zip › Figure 9/9I/MC38 tumors.tif]
