# Supplementary material for: CBX3 antagonizes IFNγ/STAT1/PD-L1 axis to modulate colon inflammation and CRC chemosensitivity
Source: EMBO Mol Med. 2024 Apr 29;16(6):10. doi: 10.1038/s44321-024-00066-6 (PMC11178889; doi:10.1038/s44321-024-00066-6)
Supplement: Supplementary file 13 — Expanded View Figures [file 44321_2024_66_MOESM13_ESM.pdf]

Expanded View Figures

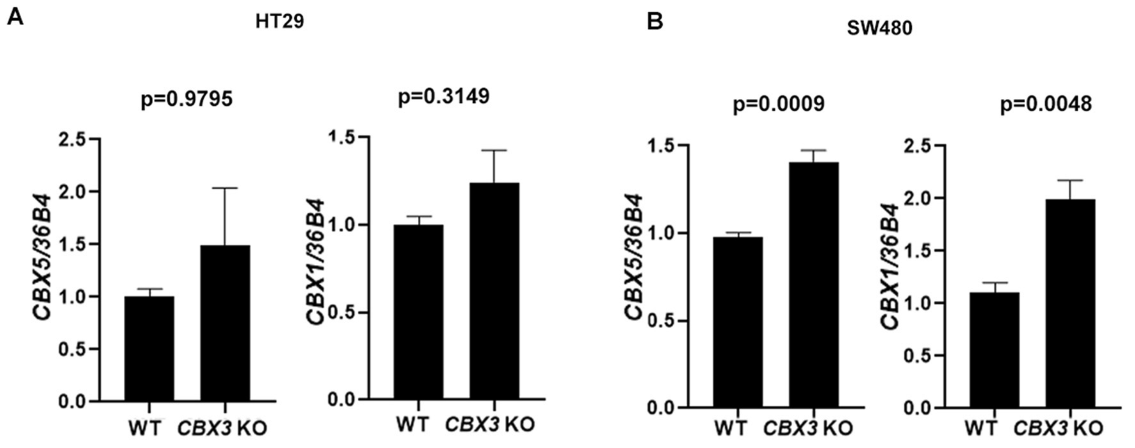

Figure EV1. Increased *CBX5* and *CBX1* mRNA levels were found in SW480 *CBX3* KO cells but not in HT29 *CBX3* KO cells.

(**A**) *CBX5* and *CBX1* mRNA levels are not significantly increased in HT29 CRISPR/Cas9 *CBX3* KO cells. (**B**) *CBX5* and *CBX1* mRNA levels are significantly increased in SW480 CRISPR/Cas9 *CBX3* KO cells. The error bar represented SEM. (4 independent experiments, two-sided t test).

© The Author(s)

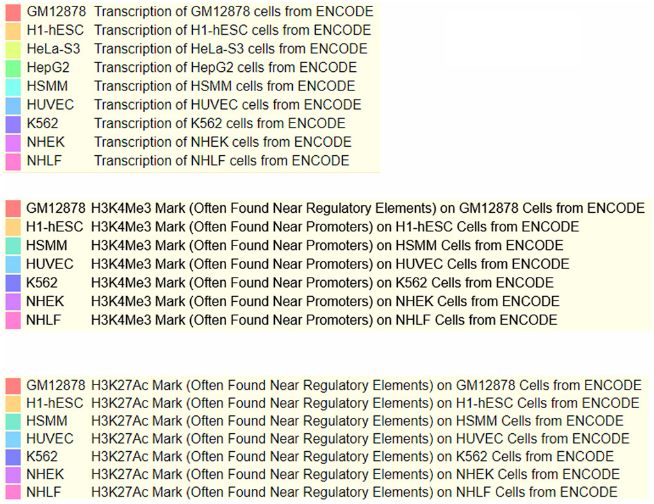

Figure EV2. The illustration of subtracks for transcription, H3K4Me3, and H3K27Ac Mark analysis from ENCODE.

 © The Author(s)

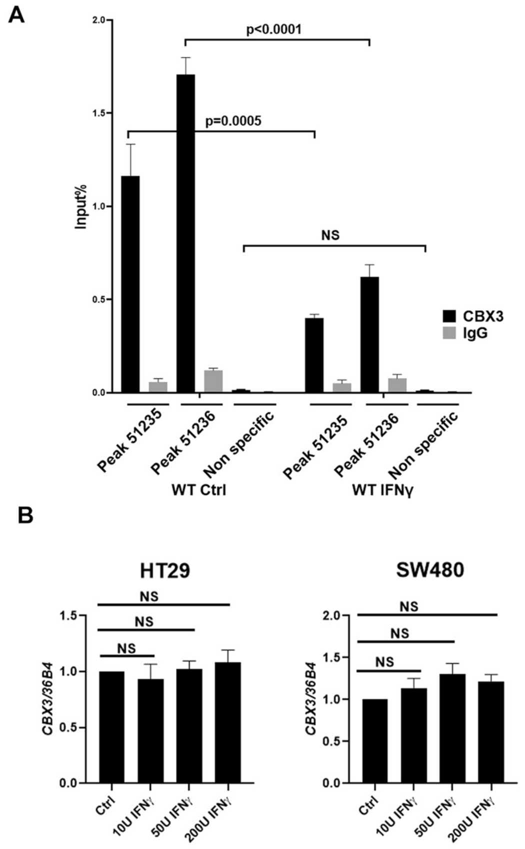

Figure EV3. ChIP-qPCR with specific primer compared to which with non-specific primers and *CBX3* mRNA level after 24h IFNγ stimulation.

(**A**) Q-PCR with specific primers to Peak 51235 and 51236 regions compared to Q-PCR with primer sets aside from the indicated CBX3 binding domain of *STAT1* gene. The error bar represented SEM (3 or 4 independent experiments, two-way ANOVA test). (**B**) 24 h IFNγ stimulation did not modified CBX3 expression in HT29 and SW480 cells at the mRNA level. The error bar represented SEM (4 independent experiments, one-way ANOVA test).

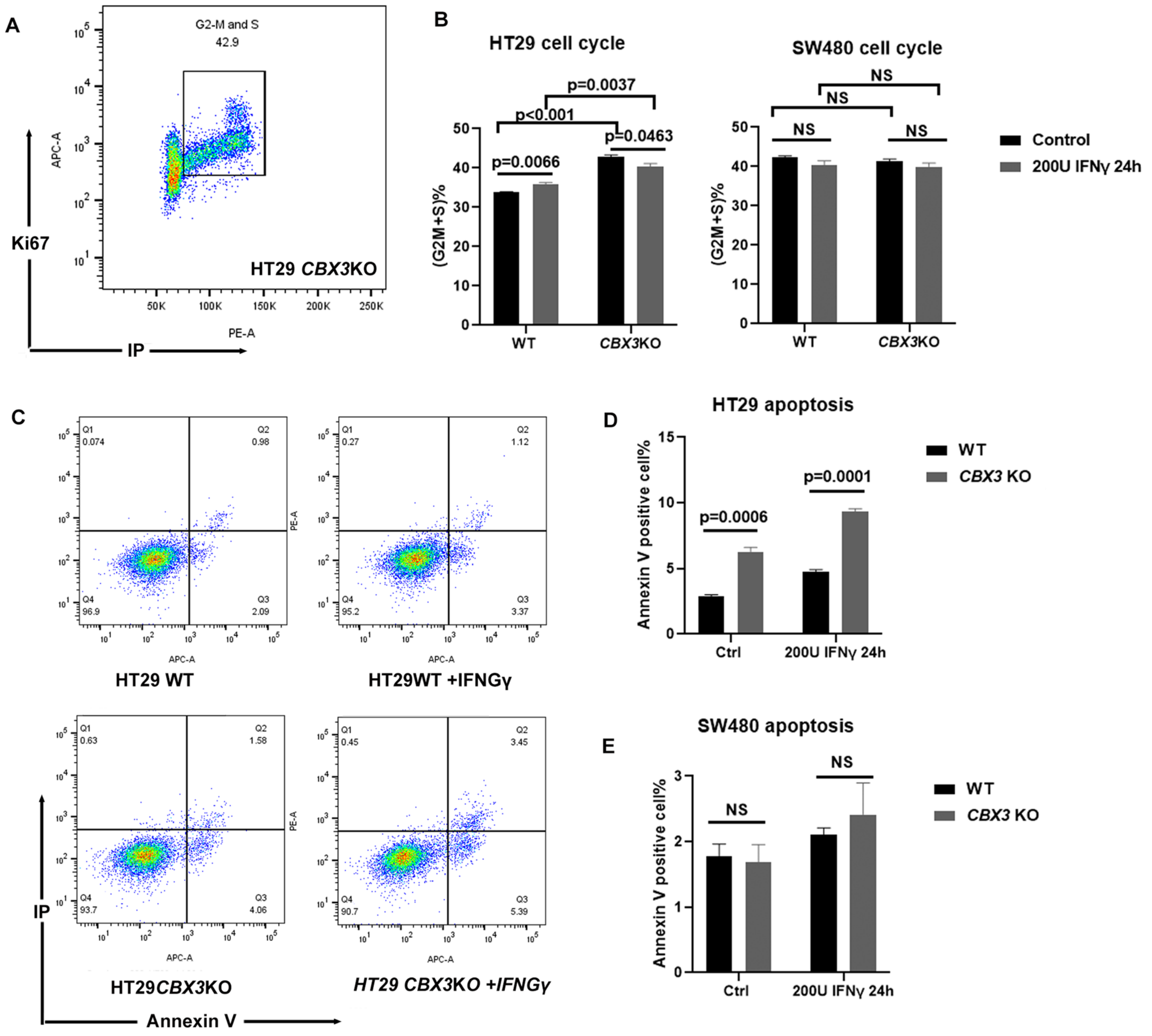

Figure EV4. The effect of CBX3 deletion and IFNγ stimulation on cell survival of HT29 and SW480 cells.

(**A**, **B**) Flow Cytometry with anti-Ki67 revealed that *CBX3* KO led to higher cycling cell in HT29 cells but 72 h of IFNγ incubation reversed this effect. These effects are not observed in SW480 cells. The error bar represented SEM (4 independent experiments, two-sided t test). (**C–E**) *CBX3* deletion makes HT29 but not SW480 cells more sensitive to IFNγ induced apoptosis. The error bar represented SEM (3 independent experiments, two-sided t test).

 © The Author(s)

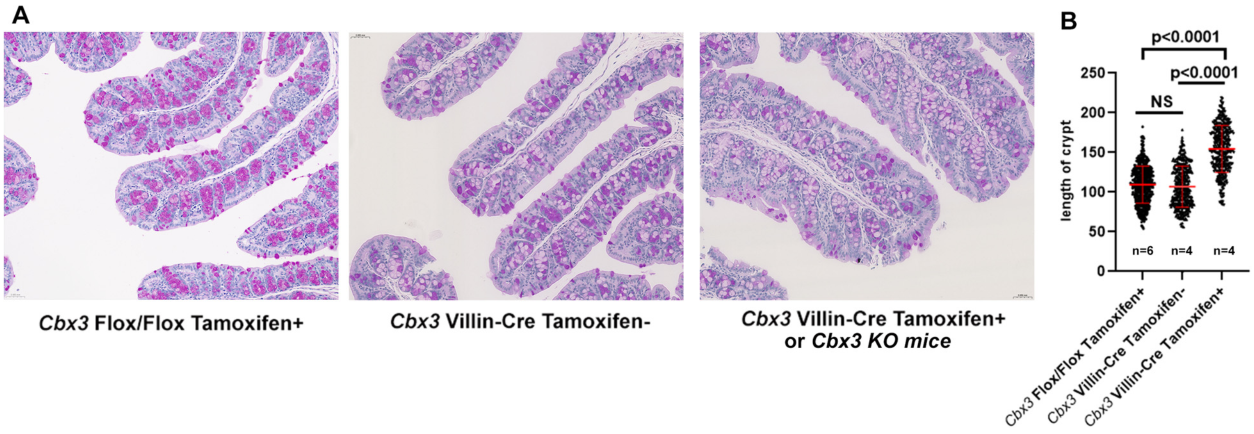

Figure EV5. Additional controls using *Cbx3*Flox/Flox mice treated with Tamoxifen.

(**A**) Crypt morphology and goblet cells density of *Cbx3*Flox/Flox mice treated with Tamoxifen show no colon inflammation criteria. (**B**) Crypt length exhibited no difference among *Cbx3* Villin-Cre mice treated with, without Tamoxifen and *Cbx3*Flox/Flox mice treated with Tamoxifen ($n = 4$ or 5, two-sided t test). The data are presented as means ± SD.
